# Multi-year data from satellite- and ground-based sensors show details and scale matter in assessing climate’s effects on wetland surface water, amphibians, and landscape conditions

**DOI:** 10.1371/journal.pone.0201951

**Published:** 2018-09-07

**Authors:** Walt Sadinski, Alisa L. Gallant, Mark Roth, Jesslyn Brown, Gabriel Senay, Wayne Brininger, Perry M. Jones, Jason Stoker

**Affiliations:** 1 U.S. Geological Survey, Upper Midwest Environmental Sciences Center, La Crosse, Wisconsin, United States of America; 2 U.S. Geological Survey, Earth Resources Observation and Science Center, Sioux Falls, South Dakota, United States of America; 3 U.S. Fish and Wildlife Service, Tamarac National Wildlife Refuge, Rochert, Minnesota, United States of America; 4 U.S. Geological Survey, Minnesota Water Science Center, Mounds View, Minnesota, United States of America; 5 U.S. Geological Survey, National Geospatial Program, Reston, Virginia, United States of America; Consejo Superior de Investigaciones Cientificas, SPAIN

## Abstract

Long-term, interdisciplinary studies of relations between climate and ecological conditions on wetland-upland landscapes have been lacking, especially studies integrated across scales meaningful for adaptive resource management. We collected data *in situ* at individual wetlands, and via satellite for surrounding 4-km^2^ landscape blocks, to assess relations between annual weather dynamics, snow duration, phenology, wetland surface-water availability, amphibian presence and calling activity, greenness, and evapotranspiration in four U.S. conservation areas from 2008 to 2012. Amid recent decades of relatively warm growing seasons, 2012 and 2010 were the first and second warmest seasons, respectively, dating back to 1895. Accordingly, we observed the earliest starts of springtime biological activity during those two years. In all years, early-season amphibians first called soon after daily mean air temperatures were ≥ 0°C and snow had mostly melted. Similarly, satellite-based indicators suggested seasonal leaf-out happened soon after snowmelt and temperature thresholds for plant growth had occurred. Daily fluctuations in weather and water levels were related to amphibian calling activity, including decoupling the timing of the onset of calling at the start of season from the onset of calling events later in the season. Within-season variation in temperature and precipitation also was related to vegetation greenness and evapotranspiration, but more at monthly and seasonal scales. Wetland water levels were moderately to strongly associated with precipitation and early or intermittent wetland drying likely reduced amphibian reproduction success in some years, even though *Pseudacris crucifer* occupied sites at consistently high levels. Notably, satellite-based indicators of landscape water availability did not suggest such consequential, intra-seasonal variability in wetland surface-water availability. Our cross-disciplinary data show how temperature and precipitation interacted to affect key ecological relations and outcomes on our study landscapes. These results demonstrate the value of multi-year studies and the importance of scale for understanding actual climate-related effects in these areas.

## 1. Introduction

Many lands managed for conservation in the north-central United States consist of interconnected wetlands and uplands [1]. These landscapes provide a range of ecosystem services critical to humans, including limiting flooding, filtering contaminants, recharging ground water, regulating climate, and providing food and recreation [1–3]. They also help sustain remarkable biodiversity, partly because wetlands support a greater number of species than suggested by the small proportion of the Earth’s surface they cover [2–4], but also because many wetland-dependent species additionally require nearby upland habitats to survive [4–6]. Amphibians are good examples of species that are dependent upon the larger landscape [6]. Some amphibian species largely or wholly are aquatic, whereas many others reproduce and perhaps overwinter in wetlands, but inhabit upland portions of the landscape seasonally while foraging, aestivating, or overwintering [7], [8]. Thus, changes in conditions throughout landscapes of interconnected wetlands and uplands can affect individual fitness and perturb amphibian populations [5], [6], [9], [10]. This is of great concern to resource managers and conservationists because many amphibian populations around the world already have declined in recent years, some precipitously, due to disease, land use, climate, and other global-change factors [10–12], and such declines could indicate effects on other species as well, including humans.

Climate change can alter the capacity of wetland-upland landscapes to provide ecosystem services and support biodiversity in various ways. These range from dramatic drought-induced reductions in surface-water availability to more subtle temperature-induced changes in trophic interactions [2], [3], [13–18]. However, actual changes in environmental conditions and ecological processes depend upon combinations of temperature and precipitation that can vary considerably within and across ecoregions over time [15], [19]. This variation, coupled with non-stationarity [20], [21] and a broad-scale lack of data from rigorous field studies of long-term climate effects in this region, contributes to widespread uncertainty regarding the nature and extent of actual climate-induced ecological changes [15], [22] at scales relevant for resource managers tasked with managing resources adaptively and effectively in the face of global change [2], [3], [23–28].

Data from long-term field research, designed to assess how temperature and precipitation are affecting key conditions, processes, and species on wetland-upland landscapes, are important for providing the information managers need regarding recent or current climate-related changes and for improving models to predict future changes with greater certainty [23], [24], [26–28]. The value of data obtained from rigorous long-term field studies is well-recognized for assessing the ecological impacts of global change (e.g., [24], [29–35]), yet such studies remain limited in the north-central United States and elsewhere.

Scientific capabilities and efficiencies for conducting long-term studies integrated across disciplines and scales have continued to improve with satellite, airborne, and ground-based sensors that allow commingled measurements of biotic and abiotic variables, including in locations that are remote or expensive to access [36–41]. As a result, collecting and analyzing data using combinations of novel and more established technologies present new opportunities to provide resource managers information they need regarding climate-driven ecological changes at meaningful scales.

In 2008, we began long-term field research to produce such information for four study areas across Minnesota and Wisconsin. This work was part of the U.S. Geological Survey’s Amphibian Research and Monitoring Initiative’s (ARMI) work to determine the statuses of amphibian populations, including assessing potential causes of any declines, in ARMI’s Midwest Region [42] and as part of the research focus on remote surveys of vegetation, water, and climate dynamics conducted at the U.S. Geological Survey’s Earth Resources Observation and Science Center (EROS). Our overarching goal was to assess annual relations between weather dynamics, key ecological conditions and processes, and the statuses of amphibian populations on wetland-upland landscapes in these areas, all within the context of recent historical climate dynamics, so we could characterize any important recent trends or perturbations and establish baseline data for assessing future changes. Our objectives were to 1) describe historical seasonal temperature and precipitation dynamics dating back to 1895 for each of those areas based upon weather-station data averaged across relevant climate divisions, 2) measure seasonal wetland water depths, and amphibian calling activity and site occupancy, via ground-based sensors deployed at a set of individual wetlands in each area from 2008 to 2012, 3) use remotely sensed data to estimate concurrent snow duration, water conditions, and photosynthetic activity across the broader landscape that encompassed each of those individual wetlands, and 4) assess relations of these ground- and satellite-based measures to temperature and precipitation data produced coincidentally at local weather stations. Our approach enabled us to examine relations among our targeted variables in novel ways, including at uniquely fine and more coarse scales, to better understand the often complex nuances of climate-related ecological changes in our study areas. This approach appears effective as a research framework for measuring actual changes happening currently and sequentially into the future, and for providing data and synthesized information local resource managers can use to improve adaptive management.

## 2. Materials and methods

### 2.1. Study areas

**Fig 1:**
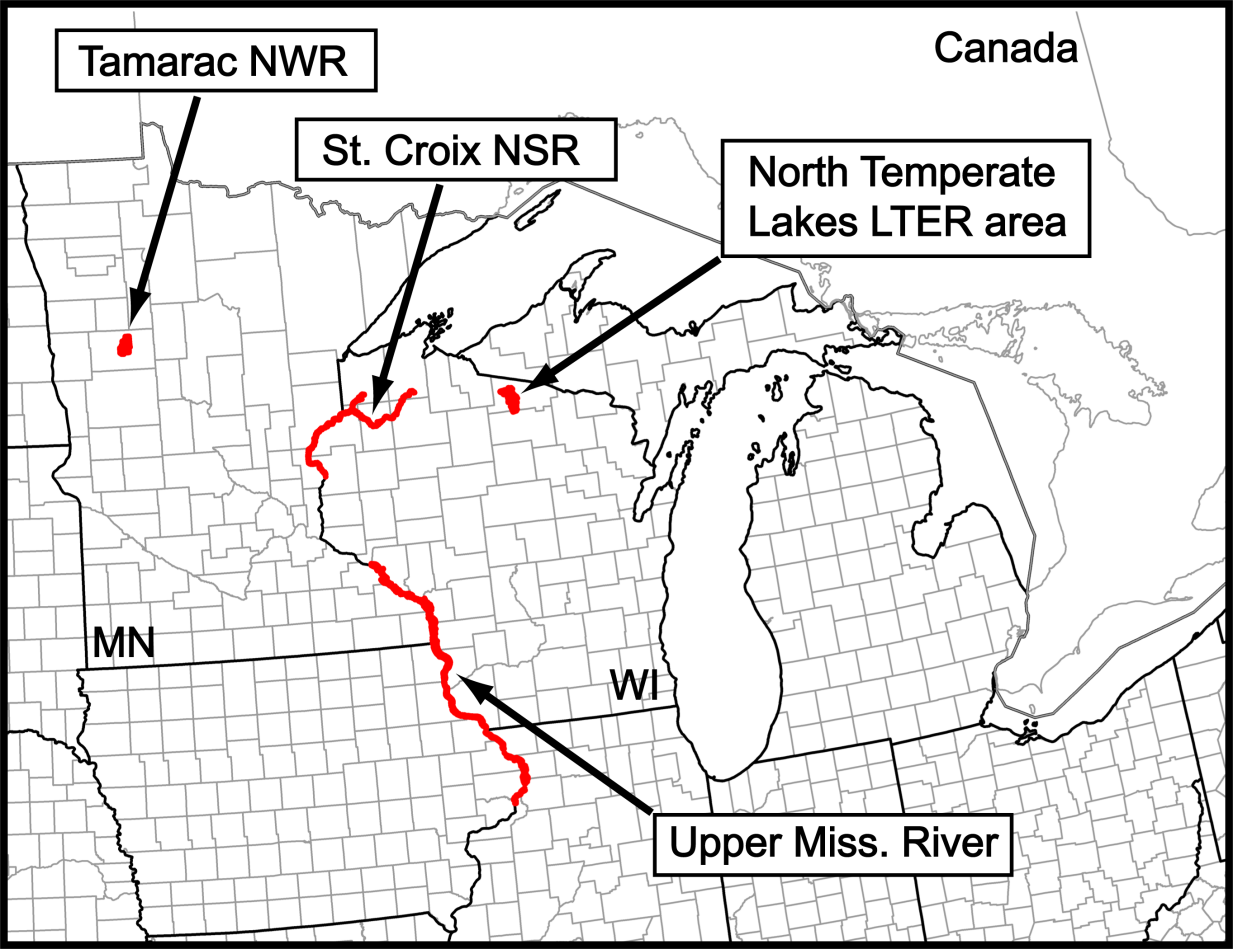
Our four study areas. NWR = National Wildlife Refuge. NSR = National Scenic Riverway. LTER = Long-term Ecological Research. Miss. = Mississippi. MN = Minnesota. WI = Wisconsin. Study-area polygons are not to scale.

We conducted field work in four study areas (Fig 1): the Tamarac National Wildlife Refuge (Tam; Minnesota, USA), the St. Croix National Scenic Riverway (SC; Wisconsin and Minnesota, USA), the North Temperate Lakes Long-term Ecological Research Area (NTL; Wisconsin, USA), and the Upper Mississippi River floodplain (UMR; Wisconsin, USA). We selected these study areas non-randomly based upon location, public ownership, previous research we conducted there, and partnerships. These four areas were located along broad east-west and north-south gradients of climate, land cover, and land use.

Our individual field sites within the NTL were in the Northern Highland-American Legion State Forest and those in the UMR were spread across the Upper Mississippi River National Wildlife and Fish Refuge, the Trempealeau National Wildlife Refuge, and Perrot State Park, the latter two of which abutted the former. The Tam and SC overlap two Level-III ecoregions [43], [44], the Northern Lakes and Forests and the North Central Hardwood Forests. The NTL is wholly within the Northern Lakes and Forests and the UMR overlaps three Level-III ecoregions, the Western Corn Belt Plains, Central Corn Belt Plains, and Driftless Area. The Tam and the two refuges in the UMR are public lands managed by the U.S. Fish and Wildlife Service. Our study sites in the SC were on public or easement lands administered by the U.S. National Park Service. Lands in the NTL and Perrot State Park of the UMR are public lands managed by the State of Wisconsin. We began this research in the SC and UMR in 2008 and in Tam and the NTL in 2009 and added the site in Perrot State Park to the UMR in 2010. The U.S. Fish and Wildlife Service, U.S. National Park Service, and State of Wisconsin provided the permits (to WS) necessary for us to work in these areas.

### 2.2. Sampling conducted from the ground

#### 2.2.1. Selection of study wetlands

We initially used a geographic information system to help structure our process for selecting individual study wetlands in each study area. This included placing a grid of square 25-ha cells over the area of inference in each study area, selecting cells randomly from the grid, and then surveying each selected cell on the ground to identify individual study wetlands. During these surveys, we selected our individual study wetland in each cell by choosing the first palustrine wetland we encountered that had the potential to be an amphibian breeding wetland based upon our experience in the region. We studied ten individual wetlands each in the Tam, SC, and NTL and five in the UMR during the years of this study.

#### 2.2.2. Measuring water depth via water-pressure loggers

We installed one water-pressure logger in each study wetland to measure water depth, except when a wetland was too deep to wade or we could not transport a kayak or canoe to it. We used a measuring pole to locate the deepest spot in each study wetland and drove a plastic pipe (anchor pipe) into the sediments to mark that location. We left the anchor pipe in place throughout the year. We installed one pressure logger (Global Water Model 14 and 15 [College Station, TX, USA] or Onset Computer Corporation Model U20-001-04 [Bourne, MA, USA], depending upon the year and site), suspended approximately 2.5 cm above the sediments, in the same spot each year alongside, and secured to, the anchor pipe soon after wetland conditions allowed. We also installed one pressure logger above the water’s surface at one or more study wetlands in each study area as necessary to measure barometric pressure for comparing with the submerged loggers’ readings. We set each of the loggers to measure pressure once per hour and used logger software supplied by Global Water and HOBOware Pro software (Onset Computer Corporation) to upload and convert data from the pressure loggers at the end of the season.

#### 2.2.3. Measuring air temperature with data loggers

During 2012, we installed one air-temperature logger per individual study wetland in all four study areas to help us determine if air temperatures within our study areas were comparable to those measured at the nearby weather station. We suspended one temperature logger (Onset Computer Corporation Model U22-001) approximately 2.5 cm below and parallel with the inner surface of a shield we manufactured to keep the logger out of direct sunlight (see S1 Appendix for more details regarding the sun shield) These loggers recorded temperature once per hour. The resultant data indicated that temperatures recorded at the nearby weather station were similar and we do not report further on those data here.

#### 2.2.4. Sampling daily precipitation totals with rain gauges

To help determine if precipitation data we obtained from the nearest automated weather stations were similar to rainfall that occurred within the boundaries of our study areas, we installed one tipping-bucket rain gauge (Onset Computer Corporation Model RG3 or Texas Electronics, Inc. [Dallas, Texas, USA] Model TR-525I) at each of two study wetlands in the Tam, SC, and NTL from 2010 to 2012. Bucket capacities were 4.73 ml and the built-in automated data loggers recorded quantities up to 5.1 cm/ h with +/- 1.0% accuracy. We calibrated each gauge according to the manufacturer’s recommendations. Data from these gauges and loggers indicated that daily precipitation totals were similar to those recorded at the nearby weather station and we do not report further on them here.

#### 2.2.5. Recording amphibian calls and other sounds with acoustic recorders

We monitored weather and snow conditions during late winter and early spring for our study areas so we could deploy acoustic recorders prior to when amphibian calling began each year (we stopped recording from late August through mid-October, depending upon the year and site). We installed one acoustic recorder (Wildlife Acoustics, Inc.; Maynard, MA, USA; Model SM1 or SM2) annually at each study wetland on the same tree at approximately two meters above the ground (see S2 Appendix for more details).

We powered each recorder with four alkaline D-cell batteries (Ray-O-Vac; Middleton, WI, USA) and preprogrammed recorders to record automatically according to the following standardized settings: five minutes at the top of every hour each day while sampling in stereo at 16000 Hz (2008 and early 2009) or 22050 Hz (2009–2012). Both sampling rates covered the frequency ranges of all amphibian calls in our study areas. See S3 Appendix for more details.

### 2.3. Sampling conducted via remote sensing

#### 2.3.1. Local landscape blocks

Using ground-based sensors to sample a set of variables only at the local scale of individual wetlands would have been limiting ecologically due to the many biotic and abiotic links between these wetlands and the broader landscape. One example of such links is that the amphibians that dwelled in our study wetlands during a season also used substantial portions of the larger surrounding landscape for various purposes over the course of a year [45], [46]. Thus, site occupancy of amphibian species at individual wetlands could reflect conditions at that wetland or across the larger landscape or both.

To try to link measurements we made at individual wetlands to ecological conditions and processes on the broader landscape, we applied a grid of square 4-km^2^ blocks covering the four study areas and then used remotely sensed data to survey each one of these blocks (local landscape block) that encompassed an individual study wetland. We decided to use 4-km^2^ landscape blocks based primarily on known life-history traits of the amphibian species we observed in our study wetlands [45], [46], but we also presumed this scale was relevant for hydrologic and other linkages as well. Within each local landscape block, we estimated the timing of snowmelt, availability of water, and levels of photosynthetic activity as indicators of key landscape conditions and processes that were linked directly and indirectly to water levels and amphibian calling activity, potential reproductive success, and site occupancy in our study wetlands. In two cases (both in SC), a single local landscape block included two individual study wetlands. Thus, we used remotely sensed data to characterize 33 local landscape blocks across all four study areas that encompassed 35 individual study wetlands in total.

#### 2.3.2. Snow presence

We acquired the Collection-5 MOD10A2 product derived with data from the Terra Moderate Resolution Imaging Spectroradiometer (MODIS) satellite sensor to determine when snow was no longer present in each local landscape block during the late winters of 2008 to 2012. This data set represented the maximum snow extent for each successive eight-day composite period from January through December at a spatial resolution of 500 m in a sinusoidal map projection [47], with pixels labeled as “snow” if snow was present for one or more days during a composite period. We defined the onset of continuous snow-free conditions as the first date of two contiguous, snow-free, eight-day composite intervals that occurred after February 15 (for the field-based analyses) or the end of February (for the remote-sensing analyses). We assumed the likelihood of amphibian calling activity or vegetation growth prior to these dates was zero across our study areas. We considered two contiguous snow-free intervals to be more relevant ecologically, given the daily variation in snowfall and snow cover that can occur during late winter and early spring in this region. For the remote-sensing analyses, we averaged the onset dates of snow-free conditions for each year across all 16 of the 500-m cells that comprised each complete 4-km^2^ block.

#### 2.3.3. Evapotranspiration

We compared rates of evapotranspiration (ET) across all local landscape blocks within each study area for 2008 to 2012 as an indicator of water availability (e.g., [48–50]) on the local landscape relative to weather conditions, the water depths we measured in our study wetlands, and to photosynthetic activity we monitored for the same blocks. We used a data set of estimated ET produced operationally at EROS as a seamless product for the conterminous United States. The data set consisted of eight-day totals of mm of ET generated with the Operational Simplified Surface Energy Balance model (SSEBop) in a geographic coordinate system at an approximate spatial resolution of 1-km^2^ [51]. This model estimated ET using several sources of data [52–56] (see S4 Appendix for more details).

#### 2.3.4. Photosynthetic activity

We used the Normalized Difference Vegetation Index (NDVI) as an indicator of photosynthetic activity. NDVI is based on the reflectance properties of chlorophyll and has been used widely to study relative quantities of green biomass (e.g., [57–60]). It is straightforward to calculate and interpret, extensively documented in the literature, and produced operationally. We used an NDVI product generated with data from “eMODIS,” a system developed at EROS to support operational use of MODIS data [61]. The eMODIS data were provided in a Lambert azimuthal equal-area projection [61] and processed using the same input data sources and the same atmospheric correction algorithms as the standard Terra MODIS MOD13Q1 Collection-5 product. However, the eMODIS process included an enhanced maximum-value compositing algorithm to filter input reflectance with bad quality, negative values, clouds, snow cover, or low view angles [61], [62]. The eMODIS NDVI product was derived from atmospherically corrected surface reflectance at a spatial resolution of 250 m and processed as a seven-day rolling composite created daily with the most recent seven days of acquisition (data are available through the EROS archive [63]). The NDVI time series was smoothed temporally using a weighted least-squares regression method [64] to reduce possible cloud contamination and atmospheric effects (S1 Fig). We used the smoothed eMODIS NDVI seven-day composites for 2008 to 2012 [65] (see S5 Appendix for more details).

### 2.4. Sources of climate data used and applications

#### 2.4.1. Data averaged across weather stations within individual climate divisions

For each season back to 1895, we downloaded the seasonal (March through August) mean daily temperature and the seasonal total precipitation from the National Oceanic and Atmospheric Administration’s (NOAA) climate dataset for the climate division that encompassed each of our study areas [66], [67]. These temperature means and precipitation totals were time-bias-corrected averages of data recorded across multiple individual weather stations within each climate division during each season [66], [67]. We used these data to describe seasonal temperature and precipitation dynamics for each of our study areas prior to and during the 2008–2012 span of this study. We relied on the quality-control measures used by the compilers and servers of these data sets and did not evaluate their quality further.

#### 2.4.2. Data from individual weather stations for relating to measurements from the ground

We downloaded daily data summaries for the individual appropriate weather stations that were nearest to each of our study wetlands and functional during 2008 to 2012 (S1 Table and S2 Table). We used these data to assess daily air temperatures and precipitation relative to the water depths and amphibian calling activity we measured during each season from 2008 to 2012. We examined all these weather-station data sets in detail to identify any missing or potentially incorrect data. See S6 Appendix for more details.

#### 2.4.3. Data from individual weather stations for relating to ET and NDVI

We obtained and prepared weather-station data for the remotely sensed analyses similar to procedures we used for our ground-based analyses. See S7 Appendix and S3 and S4 Tables for further details.

### 2.5. Analyses conducted

#### 2.5.1. Integrations for median daily temperatures across eight-day intervals in relation to measurements from the ground

Our evaluations of the timing of annual cyclo-seasonal, biotic and abiotic events linked to weather included assessing relations among air temperature, snow presence, the start of amphibian calling, and vegetation green-up for each individual study wetland/local landscape block. We described snow presence based upon satellite data collected over continuous eight-day intervals that began on January 1 of each year (§ 2.3.2). Given this time frame, we used individual weather-station data and Origin Pro software (Northampton, MA, USA) to calculate the area under the curve of mean daily temperatures for each eight-day interval and determine when the integrands first equaled or exceeded 0°C for each study area during each year from 2008 to 2012.

#### 2.5.2. Tests for relations between precipitation and water depth

To address the question of whether water depths in our study wetlands were related to rainfall, we tested the hypothesis that the number of days surface-water depths increased at individual study wetlands per year was associated with the number of days precipitation was recorded at the weather station nearest to each wetland (during the same period that we deployed depth loggers that year). We only included data from each site for those dates each year during which we had collected 24 hourly depth readings.

We tested whether the number of days wetland water depths increased was associated with the number of days of precipitation by comparing these variables 1) across all sites, study areas, and years as well as 2) only across sites within each study area across years. Based upon normality tests, we could not reject normality for the data from any of our study areas except for the UMR, where we only had instrumented two study wetlands with depth loggers. For the sake of reporting these results consistently via the same test, we used the Spearman’s rank test for all study areas, a test which does not assume normally distributed data. Our data satisfied all other assumptions. We conducted these tests using options for correlation tests in Origin Pro.

Note–we conducted exploratory analyses of relations between air temperature and water depth in our study wetlands. None were apparent and we do not report further on those analyses here.

#### 2.5.3. Acoustic data–general

We used Wildlife Acoustic’s Songscope software for various purposes in analyzing our acoustic data. Most straightforwardly, we used it to listen to individual recordings and view sounds graphically in terms of time and frequency while searching for, identifying, or confirming amphibian calls and other sounds. We also used the Songscape option of Songscope to summarize each season’s worth of recordings for each individual study wetland, which allowed us to plot these summaries and examine them visually for targeted calls or to use the data summaries to compare amphibian calling with other environmental variables we measured.

Using Songscape, we summarized each season’s batched acoustic files for each site in 100-Hz frequency steps to allow fine-scale resolution of the resultant data when graphed on contour plots. Songscape output files listed minimum, maximum, and mean dB levels (and the standard error for each mean) for each combination of recording date and time per each 100-Hz frequency step (from 0–11000 Hz).

We used the Songscape output files to create contour plots (DPlot; HydeSoft Computing, LLC; Vicksburg, MS, USA). Each plot illustrated one season's set of acoustic data for each study wetland in terms of date, recording time, sound frequency, and decibel (dB) levels, with a unique color assigned to each 4-dB interval from -88 dB (approximate level below which background noise of the recorder became problematic, as per consultation with Wildlife Acoustics) to 0 dB (the level where clipping or sound distortion occurred). We used these plots to visually identify calls of individual amphibian species and calling activity across hours and days of a season, based upon species-specific calling patterns (see next paragraph). We also used contour plots to identify when wind, rain, lightning, and other loud sounds occurred that could have obscured amphibian calls on the contour plots. The contour plots provided a remarkably informative visual summary of the biotic and abiotic sounds recorded over the course of a season at each study site, which one could view at various scales of time and frequency.

**Fig 2:**
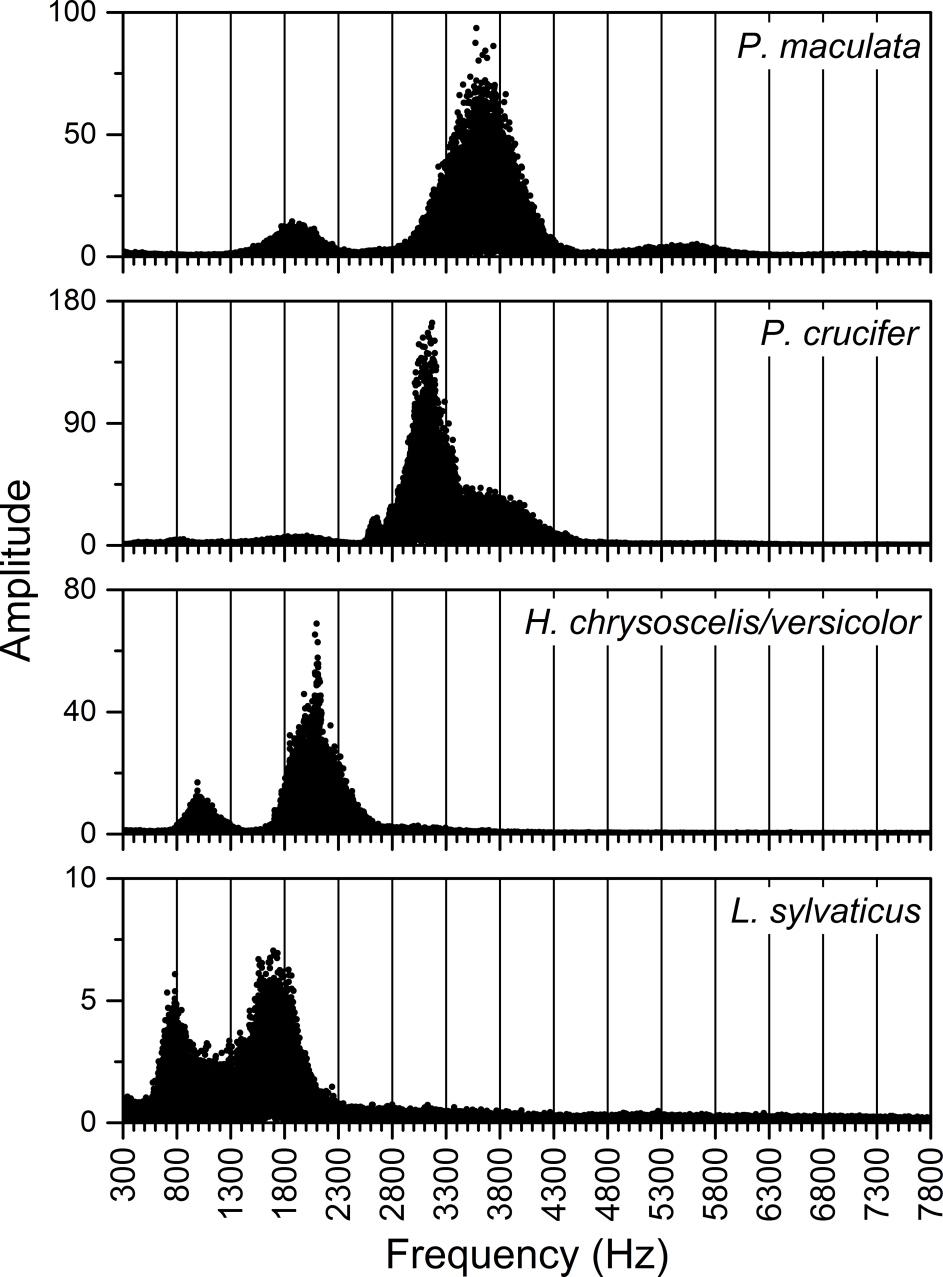
Examples of fast Fourier transforms of recordings of the calls of *Pseudacris maculata* (chorus frog), *P*. *crucifer* (spring peeper), *Hyla chyrsoscelis/versicolor* (eastern or Cope’s gray treefrog–we did not differentiate), and *Lithobates sylvaticus* (wood frog) from site SC4DB9 in the St. Croix National Scenic Riverway during 2008 (first three species) and 2009 (*L*. *sylvaticus*) showing the unique patterns of peaks in sound energy for each species.

**Fig 3:**
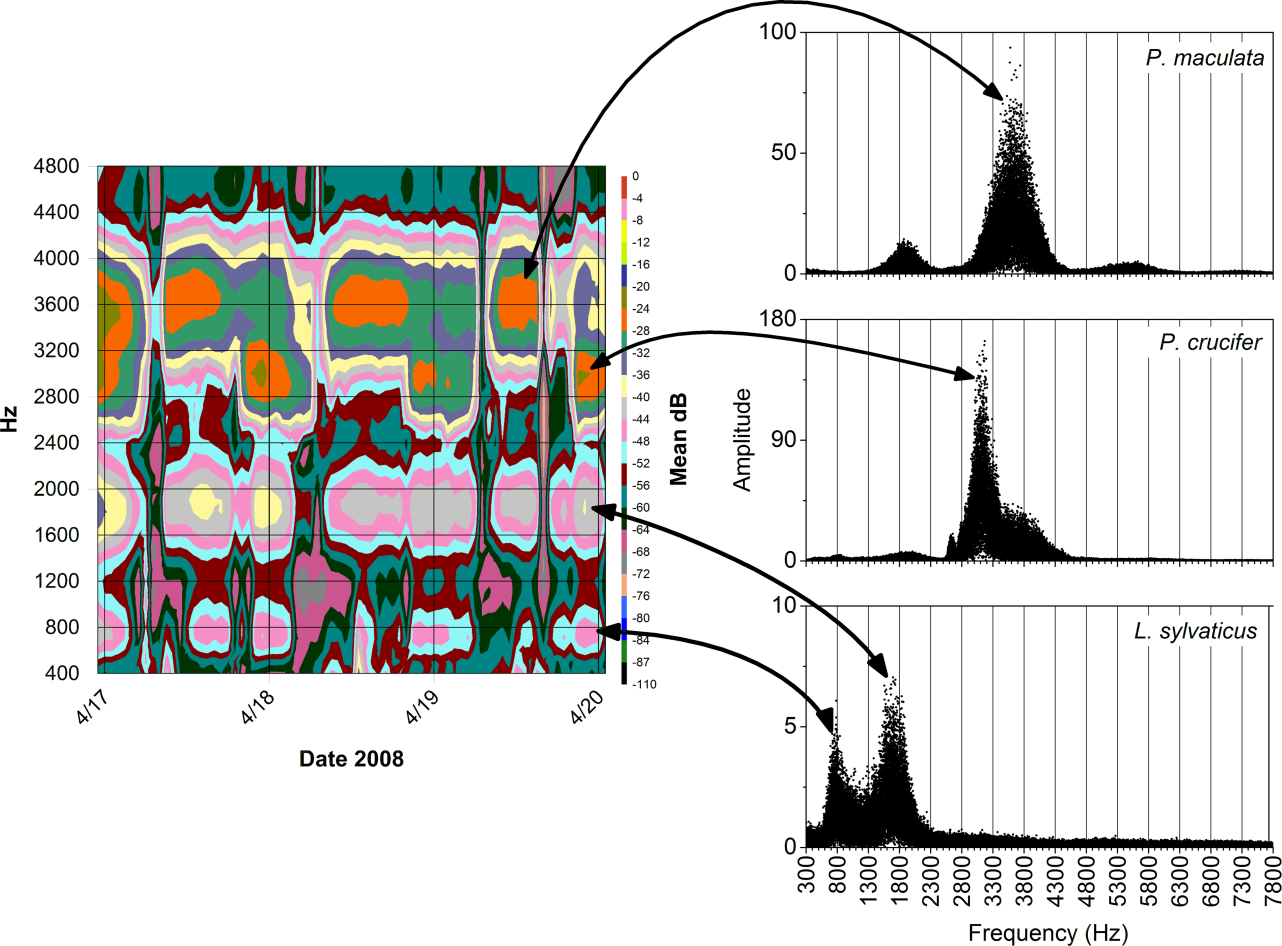
Contour plot of mean dB levels for each five-minute recording (24 recordings/day) at site SC4DB9 in the St. Croix National Scenic Riverway over three days compared with the results from fast Fourier transforms of recorded calls of *Pseudacris maculata* (chorus frog), *P*. *crucifer* (spring peeper), and *Lithobates sylvaticus* (wood frog). The unique distributions of peak sound energy in the calls of each species allowed for identification of calling activity by visually scanning summaries of batched recordings via such contour plots.

We used options available in Origin Pro to conduct fast Fourier transforms of recordings from our study wetlands and create line-graph profiles of calls made by *Pseudacris crucifer* (spring peepers), *P*. *maculata* (chorus frogs), *Hyla chrysoscelis*/*versicolor* (Cope’s gray treefrog/eastern gray treefrog–we did not differentiate between these two closely related species with very similar calls and overlapping breeding periods during our analyses), and *Lithobates sylvaticus* (wood frog) in terms of sound frequency (Hz) and intensity (dB). These profiles showed the unique harmonic series and frequency-dependent distribution of sound intensity for calls for a species (e.g., Fig 2). We then used these species-specific patterns of energy peaks to visually identify and separate species' calls straightforwardly on the contour plots described above (Fig 3). This method was highly accurate for identifying amphibian calls at our sites providing louder sounds did not obscure the species-specific patterns on the plots.

Note that WS and MR conducted all analyses of the acoustic data. Both studied amphibians in the north-central United States and elsewhere for many years, which included conducting formal call surveys and otherwise identifying regional amphibian species by their calls.

#### 2.5.4. Acoustic data–first call of the season

We used an approach we call “integrated acoustic analysis” to determine when amphibians of any species first called at each study wetland during each year. This approach involved using complementary information gleaned from our raw acoustic data in two ways. We first assessed amphibian calling activity for each site during each year via contour plots (§ 2.5.3) to determine when activity began. Whenever calls potentially were masked on a contour plot by louder sounds, we examined relevant individual five-minute recordings aurally and visually to confirm whether a call occurred (See S8 Appendix for more details).

Because we were assessing when winter conditions had diminished sufficiently to allow amphibians to become physiologically active, we considered any calling activity by any species to qualify as the start of amphibian calling activity for a site and season. One unequivocal note from one call qualified as the first call of the season, regardless of the number of calls during a recording or across recordings in a day, the number of days calling was sustained, or when peak calling activity occurred. See S9 Appendix for more details.

Due to the reliability of correctly identifying call signatures on the contour plots that were not masked, our procedure for analyzing individual recordings when calls were masked, and our field experience with the various sounds individuals of these species make, we essentially reduced any false positives and false negatives to zero or very close to it. This was a marked improvement over error rates inherent in developing and applying computer algorithms to automatically scan recordings and identify sounds by species (e.g., [36], [68], [69]).

We determined the first amphibian call of the season at each study wetland as part of our annual assessment of the timing of ecologically important cyclo-seasonal events. As described earlier (§ 2.3.2), we determined snow presence based upon satellite data collected over eight-day periods. As we did for integrations of daily mean temperatures, we described the timing of the first call of the season in terms of the running eight-day interval in which it occurred. Because of this, once we determined that an amphibian call had occurred during one eight-day interval near the beginning of the year, we did not necessarily evaluate the preceding days in that particular interval for additional calls, but proceeded to skip backwards to the last day of the previous eight-day interval to begin the sequential search for earlier calls. We evaluated all intervals or days in this manner, back to the first recording of the season. The only exceptions were occasions when weather-station data showed that air temperatures did not exceed 0°C on a specific day.

#### 2.5.5. Acoustic data–site occupancy

We used the site occupancy of *P*. *crucifer* at our individual study wetlands as a potential indicator of recent or concurrent changes in climate or other environmental conditions in our study areas. We considered *P*. *crucifer* well-suited for this purpose because 1) all our study areas were within its range, 2) each of our study wetlands provided suitable reproduction habitat for subpopulations of breeding individuals, 3) they dwell in upland habitats most of the year while foraging and overwintering, 4) they have short life spans of up to four years, 5) they are susceptible to changes in temperature, precipitation, and other environmental factors, and 6) males typically call at wetlands for a prolonged period of several weeks beginning soon after snow melts [45], [70]. These traits, in combination, suggested that changes in site occupancy for *P*. *crucifer* across seasons would reflect changes in wetland and/or upland conditions, changes potentially important to other amphibian species as well.

We used our acoustic analytic approach to determine presence/absence for *P*. *crucifer* at each of our study wetlands per year. The only difference in how we analyzed our acoustic samples for *P*. *crucifer* calls to measure presence/absence from how we analyzed our samples for the first call (any amphibian species) per season was in terms of the temporal extent to which we searched samples for calls of *P*. *crucifer*. For annual presence/absence, once we observed an unequivocal call signature of *P*. *crucifer* anywhere on a contour plot, we simply recorded the species as present for that site and year. When we did not observe such an unequivocal call signature, we visually and aurally surveyed all relevant five-minute recordings for which the contour plot suggested a possible call signature or when other sounds had possibly masked calls. We surveyed all such relevant recordings from when a recorder was first deployed and snow and weather conditions had reached suitable thresholds through the first week of June, which was when calling typically was complete for this species at our study wetlands. We also surveyed recordings made later in June if calling signatures were apparent or suggested on the contour plot. Such later calling was unusual, but did occur in 2010, for example.

We sampled the calling activity of *P*. *crucifer* and other sounds at each study wetland via the acoustic recorders for five minutes at the top of each hour each day (§ 2.2.5) throughout the entire calling season and beyond. Our method for assessing presence/absence for *P*. *crucifer* across these extensive data sets resulted in detection rates at or near 100% and false positives and false negatives at or near zero. Thus, we did not model occupancy *per se* and report the results here as the median number of sites occupied per study area per year.

#### 2.5.6. Acoustic data–seasonal calling dynamics relative to environmental conditions

To better understand relations between the daily calling activity of *P*. *crucifer*, weather conditions, and water depth, we compared these dynamics for site SC4DAI2 in the SC as a case study from 2008 to 2012. From the summary acoustic data sets we generated annually for SC4DAI2, we extracted the hourly median dB levels from 2900 to 3200 Hz and the hours from 2100 to 2300 for each day of the period from the date *P*. *crucifer* first called to the date when they last called and considered any day in this range as one during which *P*. *crucifer* potentially could call. Because calling activity could vary from one hour to the next, we used Origin Pro to integrate the area under the curve for the daily median dB levels from 2900 to 3200 Hz during 2100 to 2300 h and used the integrand to represent the relative sound intensity for *P*. *crucifer* across those hours for each date. Similar to using unique call signatures on contour plots to identify individual species, this enabled us to describe relative *P*. *crucifer* calling activity by focusing on the unique narrow frequency range that captured the highest energy band, i.e., the most heavily accentuated harmonic (*sensu* [71]), in the calls of *P*. *crucifer* (Fig 2) and during the hours over which *P*. *crucifer* typically called most often and most intensively at our study wetlands. See S10 Appendix for more details.

#### 2.5.7. Acoustic data–calling peaks and phenophases

We selected six sites in the SC as case studies to address the question of whether phenophases, the period from the first to the last call, and the median of the peak calling events varied for *P*. *crucifer* in relation to the start of season from 2008 to 2012 and, similarly, whether the first call for *H*. *chrysoscelis/versicolor* (which breed later in the season) varied annually in relation to the start of each season. We limited this analysis to six sites because it was labor-intensive and chose these sites because we began collecting data for most of them in 2008, acoustic recorders operated relatively reliably at these sites across years, and these sites were most consistent among the SC sites in containing surface water and having these species call across years. We used our acoustic analytic approach (§ 2.5.4) to determine the specific first and/or last call dates for each species. We identified the top three peak calling dates for *P*. *crucifer* (after we had filtered dates with overlapping sounds) based upon the integrands we calculated for the bandwidth most specific to this species and during the daily times when they typically called the most (§ 2.5.6) and then used the median of those three dates as the peak for each season.

#### 2.5.8. Relations between remotely sensed data and weather conditions

In contrast with sampling via sensors on the ground, during which we controlled the specific wetlands being sampled and placement of sensors, sampling schedules, and sample rates (for acoustic sampling), we had no control over the Terra MODIS sensor regarding locations sampled, spatial resolution, repeat sampling cycles, or the energy bandwidths sampled. Furthermore, we used available MODIS-derived products for snow, ET, and NDVI that were developed at different temporal and spatial resolutions and in different map projections (§ 2.3.2–2.3.4). We standardized spatial and temporal characteristics across these data sets to obtain a consistent framework for comparing relations between ET, NDVI, and intra- and inter-annual changes in air temperature, precipitation, and the timing of snow-off.

We converted all remotely sensed data to the Albers equal-area conic projection using parameter settings commonly applied by the U.S. Geological Survey for conterminous U.S. products (e.g., see [72]). We used weeks (seven-day intervals beginning on January 1 of each year) as our standard time interval for analyses. We cross-walked the (approximately) 52 annual time steps of NDVI data to a standard weekly schedule and did the same with the 46 annual time steps of the ET data (see S5 Table). As a result of this approach, we did not have ET data that corresponded with weeks 5, 13, 21, and 29, but we preferred this solution to the alternative, which would have caused us to discard NDVI data to standardize to eight-day intervals.

#### 2.5.9. Relations between ET and weather conditions

We tested whether ET was associated with precipitation and temperature measured at nearby weather stations for each local landscape block across our study areas. We summarized temperature data in terms of growing degree units (also known as growing degree days). Growing degree units (GDU) equal the difference (in degrees) between the average daily temperature and a baseline temperature necessary for photosynthesis (and, thus, transpiration) to occur during a 24-hour period and generally are reported as accumulated GDU over the growing season. The concept typically has been used to estimate seasonal conditions for different crop types [73] and the baseline has been selected accordingly [74], [75]. Various researchers have applied GDU successfully in studies reliant upon remotely sensed data (e.g., [74–78]). We calculated GDU following “Method 2” in McMaster and Wilhelm [73] and calibrated the units to the regions in which our study sites were located, using 10 °C as a baseline temperature and setting an upper limit of 30 °C, above which we expected vegetation growth rates would slow to reduce water loss (e.g., [79]). We tested the extent, if any, to which ET was associated with GDU and precipitation via the Spearman’s rank coefficient using the CORR procedure in SAS® software (v. 9.3, SAS Institute Inc., Cary, North Carolina, USA) at three time scales: weekly, running four-week (incremented weekly), and cumulatively from January to August (to-date accumulation) incremented weekly. We presumed these scales would encapsulate recent weather, growing conditions ameliorated by moisture reserves in the soil profile, and the overall seasonal trajectory of growing conditions, respectively.

We constructed simple climatographs (*sensu* [80]) for data from each weather station based upon a constant four-week interval, incremented weekly, to indicate when precipitation inputs may have been inadequate to meet ET demand, given air temperatures. We assumed that a constant ET-equivalency threshold of 20 mm of total precipitation and an average daily temperature of 10 °C per month provided a coarse, yet plausible, estimate of the threshold between water surpluses and water deficits [81] for our study areas, despite not incorporating any information on soils, vegetation, photoperiod, or other environmental factors known to influence ET. We recognized that these graphs might not reflect all water stress/deficits adequately, but we simply used them as rough guides to help interpret seasonal ET (and NDVI) dynamics in terms of the timing of the onset of ET activity, its early-season magnitude, and total seasonal ET.

#### 2.5.10. Relations between photosynthetic activity and weather conditions

We calculated the average NDVI within each local landscape block. As with the ET data, we summarized total NDVI at weekly, four-week running, and seasonal to-date time scales and tested for associations between these individual metrics and GDU and precipitation using the Spearman’s rank coefficient.

We manually determined the timing of vegetation green-up in each of our local landscape blocks because automated algorithms can be influenced by false green-up, resulting from intermittent snow cover, particularly in areas with evergreen vegetation (e.g., see S2 Fig). We examined the phenological response curves from 2008 to 2012 for each block and subjectively determined a winter (background) NDVI level for that block across all five years. We then averaged the winter NDVI levels across all blocks in each study area (Tam = 0.34, SC = 0.45, NTL = 0.51, UMR = 0.31) and determined that 0.60 was a conservative threshold above which greenness exceeded winter background levels [82]. Vegetation start-of-season (green-up) for each block then was the first post-February week when NDVI values rose above this level. We compared the vegetation start-of-season with the timing of snow-free conditions and the onset of GDU and also assessed the number of weeks for which NDVI values within each block were > 0.60 through August.

## 3. Results

### 3.1. 2008 to 2012 seasonal weather data in historical context

#### 3.1.1. Temperature

**Fig 4:**
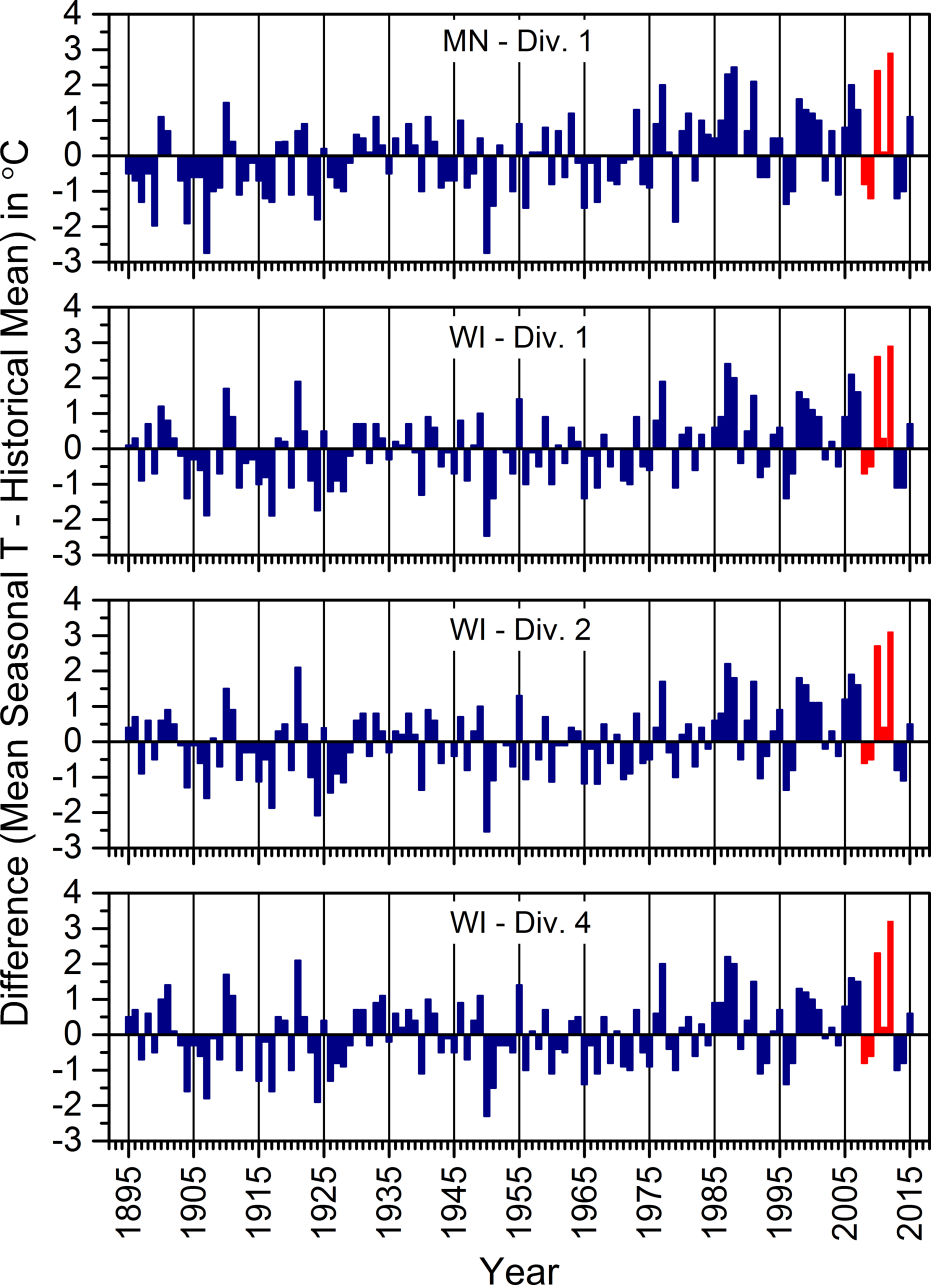
The difference between the average of mean daily temperatures across March to August of each year and the overall mean of such seasonal averages from 1895 to 2015. Seasonal averages for March to August were based upon data corrected for time bias and averaged across weather stations in each climate division identified on each graph. The Tamarac National Wildlife Refuge, St. Croix National Scenic Riverway, North Temperate Lakes Long-term Ecological Research area, and Upper Mississippi River were in MN-Div. 1, WI-Div. 1, WI-Div. 2, and WI-Div. 4, respectively. Red bars indicate the years of our field study (2008 to 2012). See S1 Table for information regarding the source of the climate data.

Prior to the mid-1970s, the means of daily temperatures from March through August mostly were lower than the historical (1895 to 2015) mean of such temperatures across all four climate divisions encompassing our study areas, except during the relatively warmer 1930s (Fig 4). Since the 1970s, such temperatures mostly were warmer than the historical mean across all four climate divisions (Fig 4).

**Fig 5:**
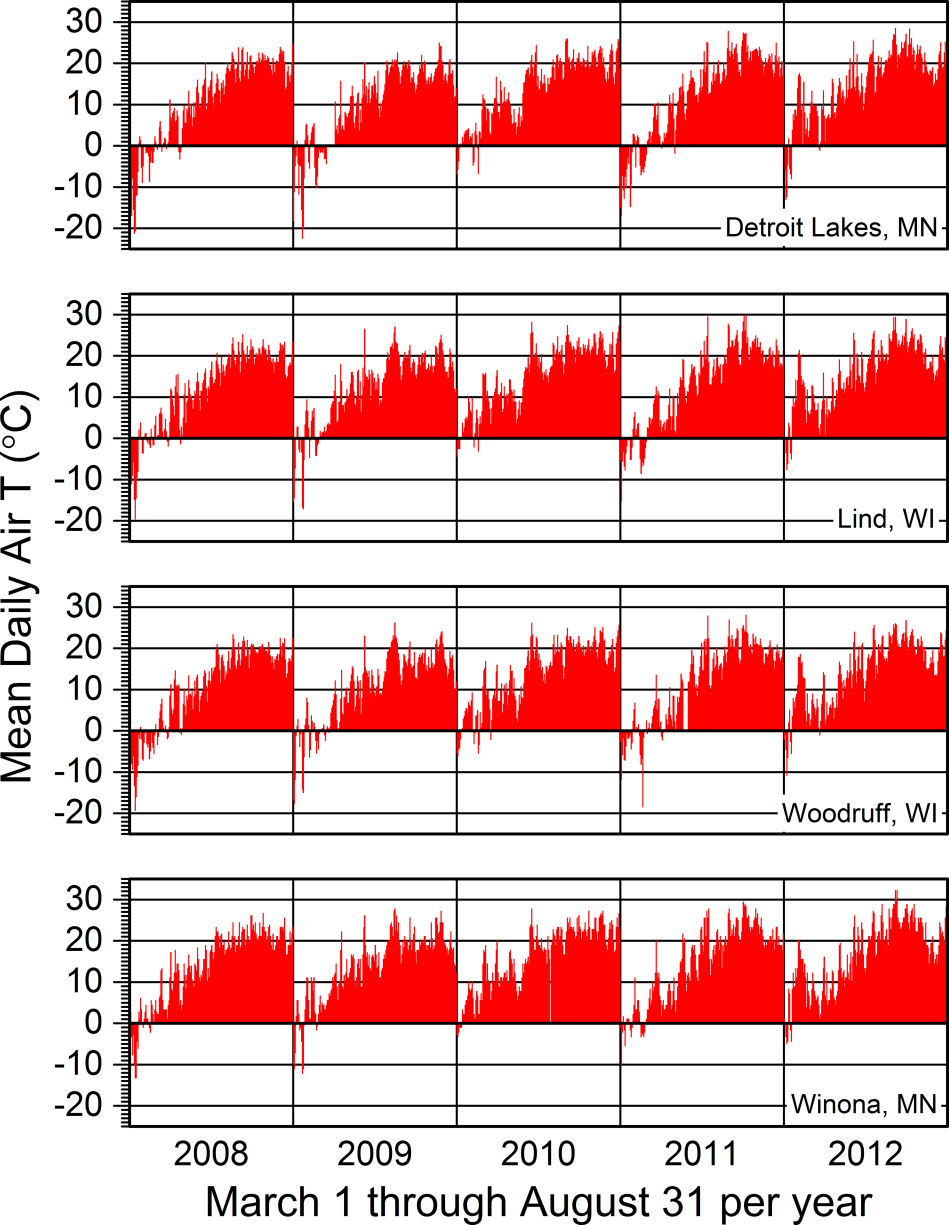
Mean daily air temperatures for each season across years. Detroit Lakes, MN = data from the weather station we used for the Tamarac National Wildlife Refuge. Lind, WI = data from the weather station we used for sites near the approximate middle of the St. Croix National Scenic Riverway. Woodruff, WI = data from the weather station we used for sites in the North Temperate Lakes Long-term Ecological Research area. Winona, MN = data from the weather station we used for sites near the approximate middle of the Upper Mississippi River. Missing data for Detroit Lakes in 2009 and 2012, Woodruff in 2011, and Winona in 2010 are due to missing data, not 0 values. Otherwise, 0 values are due to mean daily air temperatures of 0 °C. See S1 Table and S2 Table for information regarding the specific weather stations.

**Fig 6:**
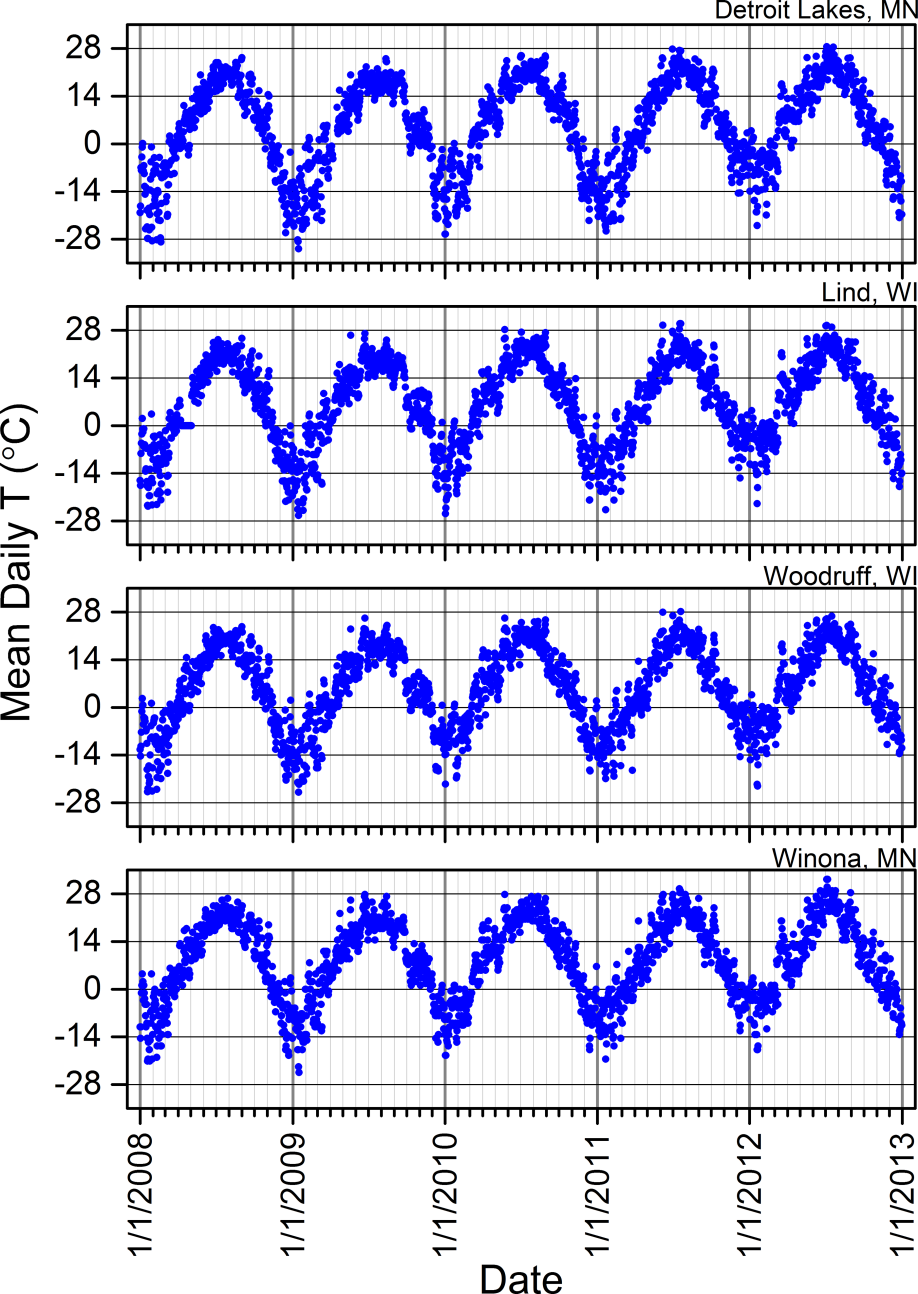
Mean daily air temperatures across the years 2008 to 2012 based upon data from individual weather stations near our study sites. Detroit Lakes, MN = data from the weather station we used for all sites in the Tamarac National Wildlife Refuge. Lind, WI = data from the weather station we used for four sites in the St. Croix National Scenic Riverway. Woodruff, WI = data from the weather station we used for all sites in the North Temperate Lakes Long-term Ecological Research area. Winona, MN = data from the weather station we used for three sites in the Upper Mississippi River. Temperature data were missing from weather-station data sets for some dates. See S1 Table and S2 Table for information regarding the specific weather stations.

Seasonal averages of mean daily air temperatures for 2010, 2011, and 2012 were greater than the historical mean across all four climate divisions (Fig 4). Two-thousand-and-twelve ranked as the warmest season on record dating back to 1895 for all four regions, whereas 2010 ranked as the second warmest in each division except the one that included Tam (Fig 4), where it was the third warmest. Thus, average seasonal temperatures for 2010 and 2012 were exceptionally warm in these climate divisions on this time scale. Daily temperatures measured at individual weather stations closest to our study wetlands showed that the relatively warmer seasons in 2010 and 2012 largely were due to higher temperatures in March or early April (Fig 5). They also suggested that although summer and winter temperatures generally appeared to be warming annually across all four study areas, warmer winter temperatures in 2010 and 2012 likely facilitated the relatively early snowmelt and warmer temperatures we observed in March of both years (Fig 6). In contrast, our work during 2008 and 2009 occurred when seasonal average temperatures were cooler than the historical mean, whereas the 2011 season was just above the mean (Fig 4). Thus, our observations of weather-related ecological relations during this study occurred over a range of seasonal temperatures.

#### 3.1.2. Precipitation

**Fig 7:**
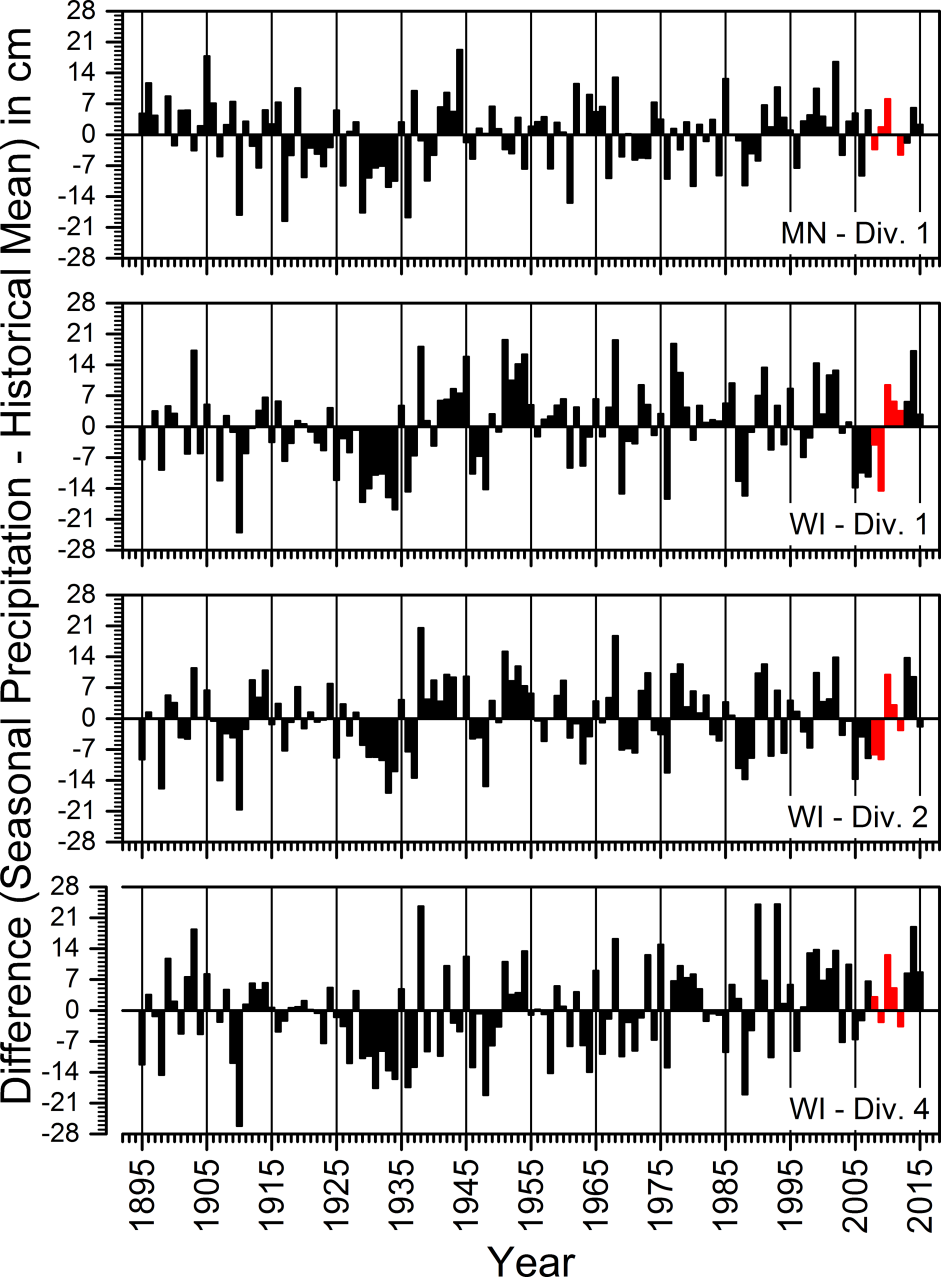
The difference between the total precipitation for March to August of each year and the overall mean of such seasonal precipitation from 1895 to 2015. Seasonal totals for March to August were based upon data corrected for time bias and averaged across weather stations in each climate division identified on each graph. The Tamarac National Wildlife Refuge, St. Croix National Scenic Riverway, North Temperate Lakes Long-term Ecological Research area, and Upper Mississippi River were in MN-Div. 1, WI-Div. 1, WI-Div. 2, and WI-Div. 4, respectively. Red bars indicate the years of our field study (2008 to 2012). See S1 Table for information regarding the source of the climate data.

**Fig 8:**
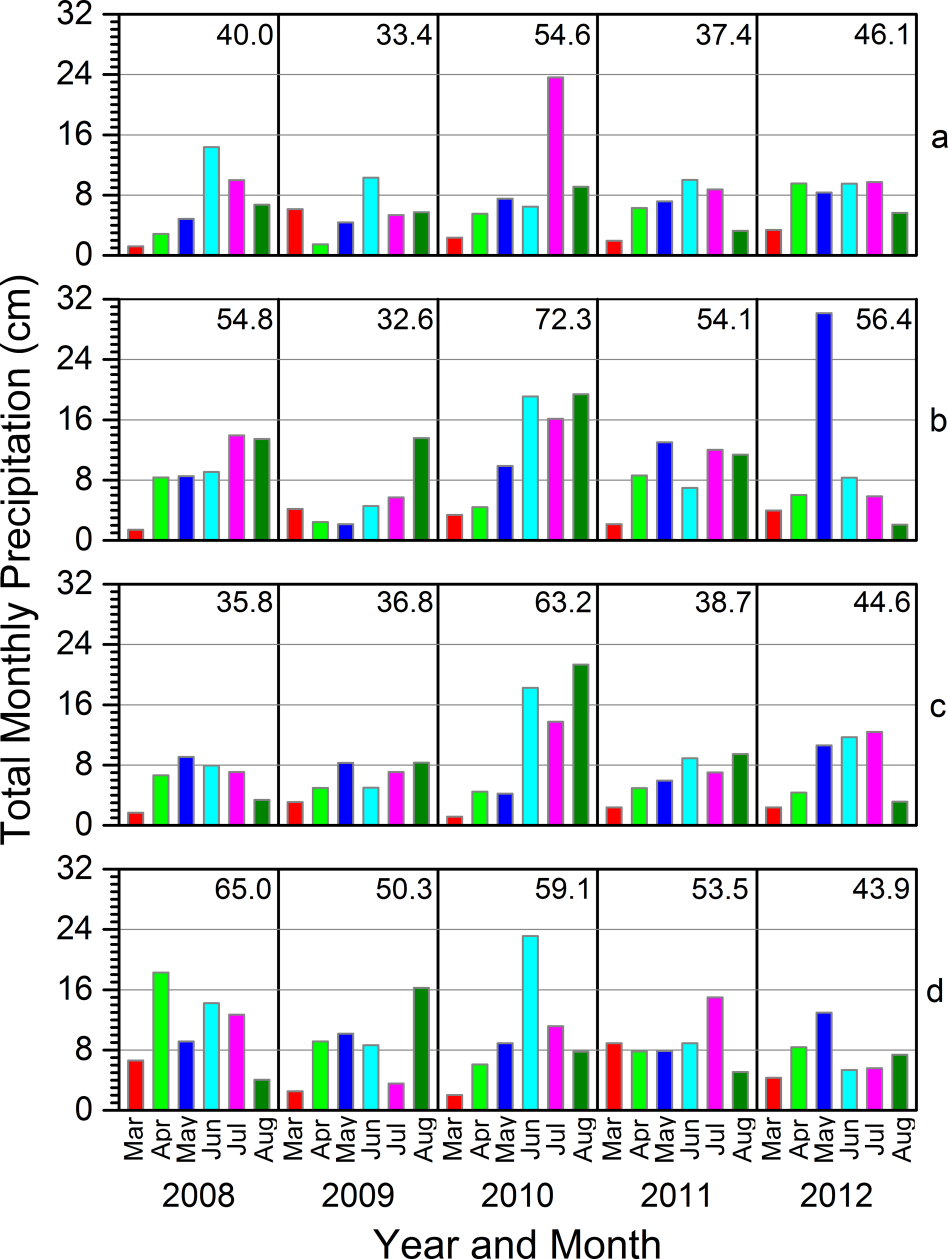
Total monthly precipitation by year based upon data collected at individual weather stations near our study sites in each study area. The number in the upper section of each graph = the total precipitation for March through August of that year. Data from weather stations in Detroit Lakes, MN (Tamarac National Wildlife Refuge), Lind, WI (mid-St. Croix National Scenic Riverway), Woodruff, WI (North Temperate Lakes Long-term Ecological Research area), and Trempealeau, WI (mid-Upper Mississippi River), are represented by graph sections a, b, c, and d, respectively. See S1 Table for additional information regarding the sources of the climate data.

Unlike temperature, daily total precipitation averaged across March through August did not show consistent recent trends across all the climate divisions encompassing our study areas. Wetter seasons did dominate in the regions containing the Tam and UMR since about 1990 and groups of wetter and drier years alternated similarly in the regions containing SC and NTL since about the late 1980s (Fig 7). During the years of this study, seasonal average daily precipitation in 2009 was near the historical mean in the climate divisions containing the Tam and UMR, but relatively low in the divisions encompassing the SC and NTL, whereas 2010 seasonal averages were relatively high across all four divisions (Fig 7). Monthly and seasonal total precipitation, based upon data from individual weather stations nearest to our study wetlands in each study area, varied substantially within and across study areas and seasons from 2008 to 2012 and illustrated important precipitation dynamics that were masked by seasonal averages (e.g., Fig 8), similar to how important temperature dynamics were masked at the coarser spatial scale of climate divisions. As with temperature, our observations of weather-related ecological relations from 2008 to 2012 occurred over a range of precipitation conditions.

### 3.2. Timing of annual cyclo-seasonal events

**Fig 9:**
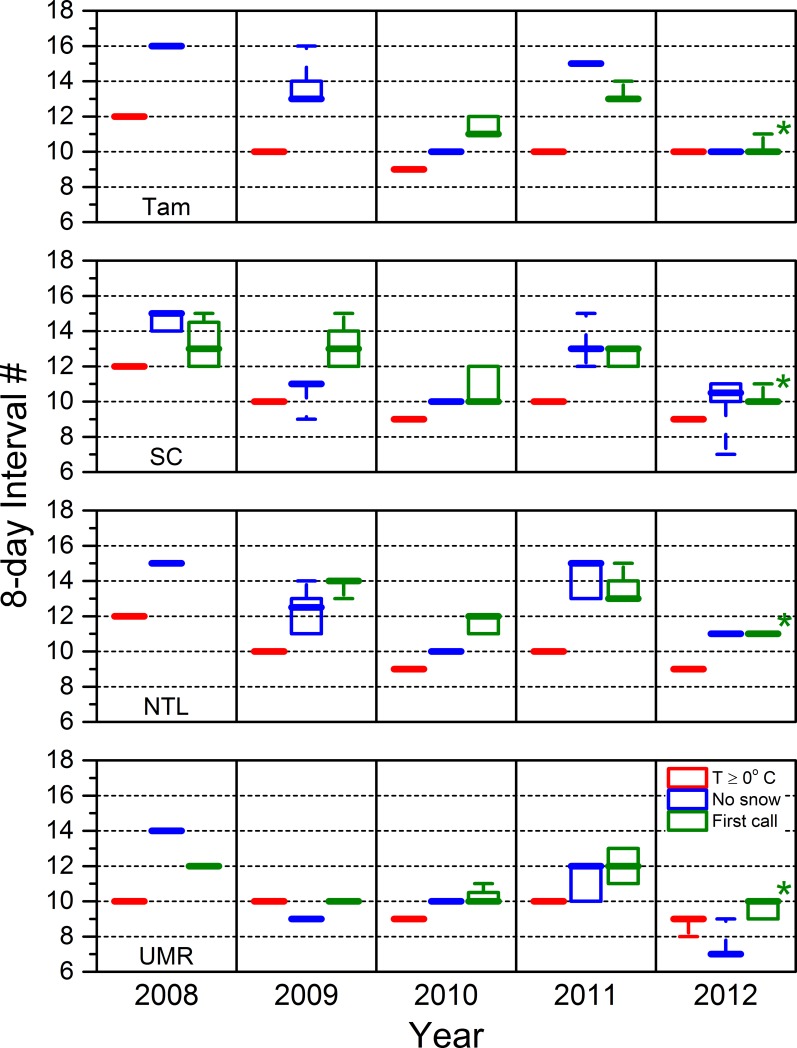
The first eight-day intervals during which the mean daily air temperature was ≥ 0 °C, snow was absent for at least two consecutive weeks, and the first amphibians called. The range of these boxplots includes the middle 50% of the data (25 to 75%). The whiskers extend out to the minimum and maximum values. The median value is represented by the extra thick line. An asterisk indicates that calling already had begun by the date we deployed recorders and almost certainly had begun one interval earlier. Tam = Tamarac National Wildlife Refuge. SC = St. Croix National Scenic Riverway. NTL = North Temperate Lakes Long-term Ecological Research area. UMR = Upper Mississippi River.

**Fig 10:**
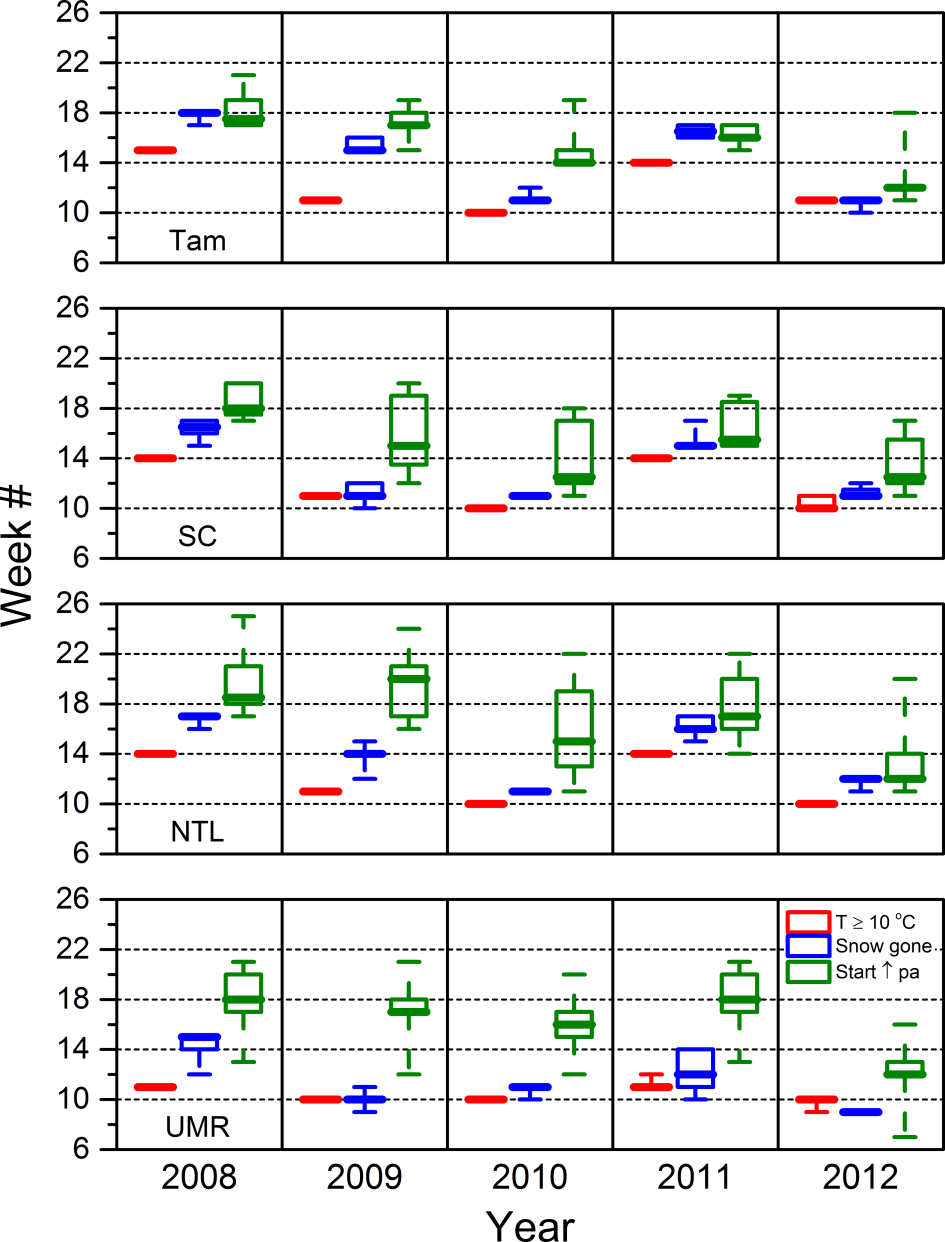
The first week during which the mean air temperature was ≥ 10 ºC (the threshold necessary for photosynthetic activity to occur), snow was absent, and vegetation green-up began. The range of these boxplots includes the middle 50% of the data (25 to 75%). The whiskers extend out to the minimum and maximum values. The median value is represented by the extra thick line. Tam = Tamarac National Wildlife Refuge. SC = St. Croix National Scenic Riverway. NTL = North Temperate Lakes Long-term Ecological Research area. UMR = Upper Mississippi River. pa = photosynthetic activity.

Median air temperatures ≥ 0 ºC (over an eight-day interval) occurred earliest in 2010 and 2012 in all our study areas except Tam, where they occurred during the same interval in 2012 as they did in 2009 and 2011 (Fig 9). Median air temperatures ≥ 0 ºC occurred latest in 2008 in all study areas except the most southerly UMR, where they occurred during the same interval in 2008 as they did in 2009 and 2011 (Fig 9). Apparent snow-free conditions occasionally occurred earlier than or within the same eight-day time interval as when median temperatures reached 0 ºC, but occurred more often during a subsequent interval up to five intervals later (Fig 9). Air temperatures suitable for vegetation growth (weekly median temperature ≥10 ºC occurred earliest in 2010 followed by 2012 and 2009 and later in 2008 and 2011. Such growing conditions almost always occurred within a week of when median air temperatures rose above freezing, except in 2011, when growing conditions occurred three weeks later in all study areas except the UMR (Fig 10).

We did not observe the first amphibian calls of the season at any of our study wetlands before median eight-day interval temperatures were ≥ 0 ºC (Fig 9). We did, however, occasionally observe first calls while snow still was present on the surrounding landscape (Fig 9). Calling began notably earlier in 2010 and 2012 across all study areas, whereas the start of calling was more similar in 2008, 2009, and 2011 in study areas for which we had data for all five years. The start of calling in the UMR during 2009 was an exception to such similarities, as calling in that study area and season began about the same time as it did in 2010 and 2012 (Fig 9).

**Fig 11:**
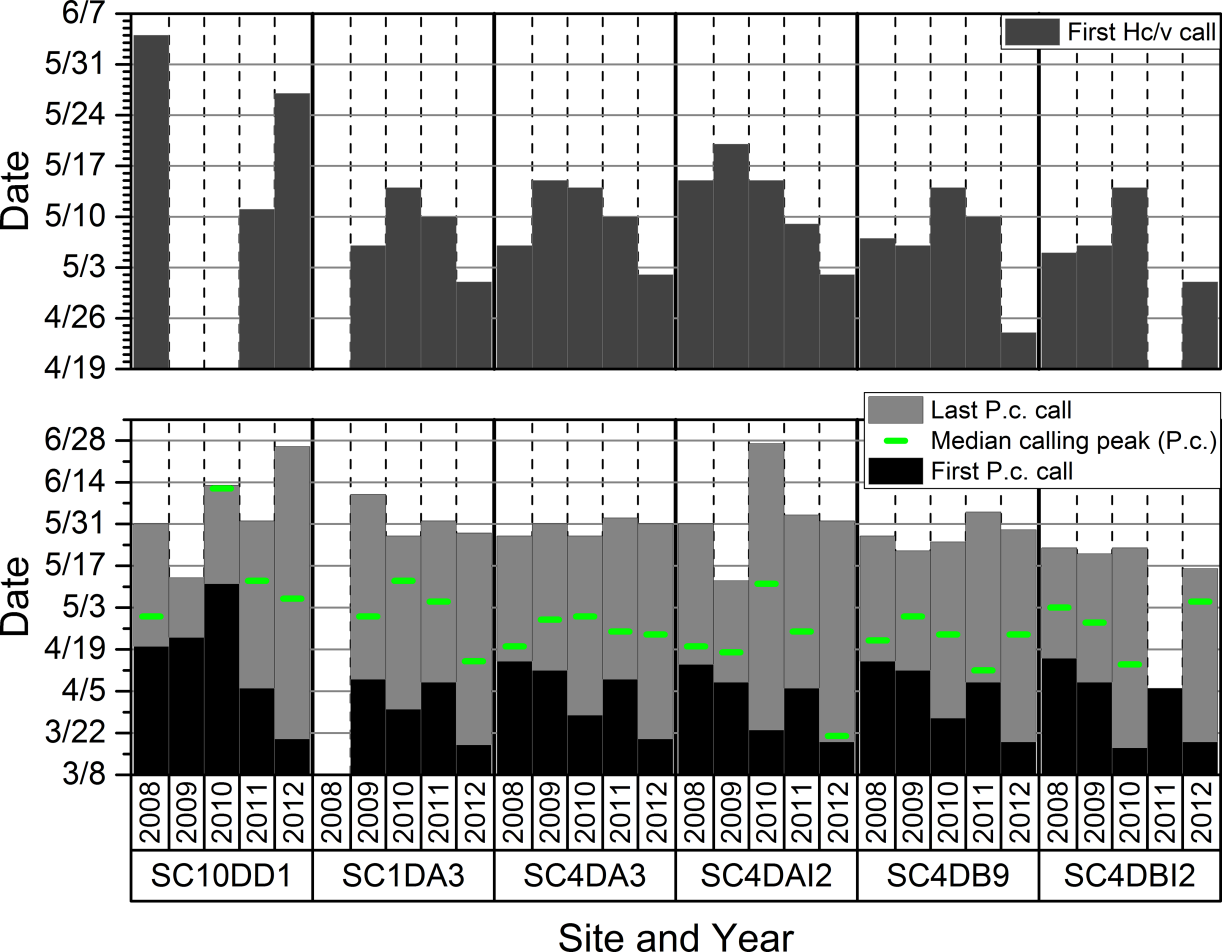
The timing of the first and last calls and median calling peaks for *Pseudacris crucifer*, and the first calls of *Hyla chrysoscelis/versicolor*, across seasons at six study wetlands in the St. Croix National Scenic Riverway. Some site data are missing in certain years because we did not install an acoustic recorder (SC1DA3 in 2008), the recorder malfunctioned (SC4DBI2 in 2011), or limited surface water affected calling activity (SC10DD1).

Phenophases and median calling peaks for *P*. *crucifer* were not related consistently to when the season began for the six select study wetlands we evaluated in the SC (Fig 11). First calls of the season for *H*. *chrysoscelis/versicolor*, which typically began calling in May, were earliest in 2012 across four of the five sites, but not earlier in 2010 relative to the other years (Fig 11).

**Fig 12:**
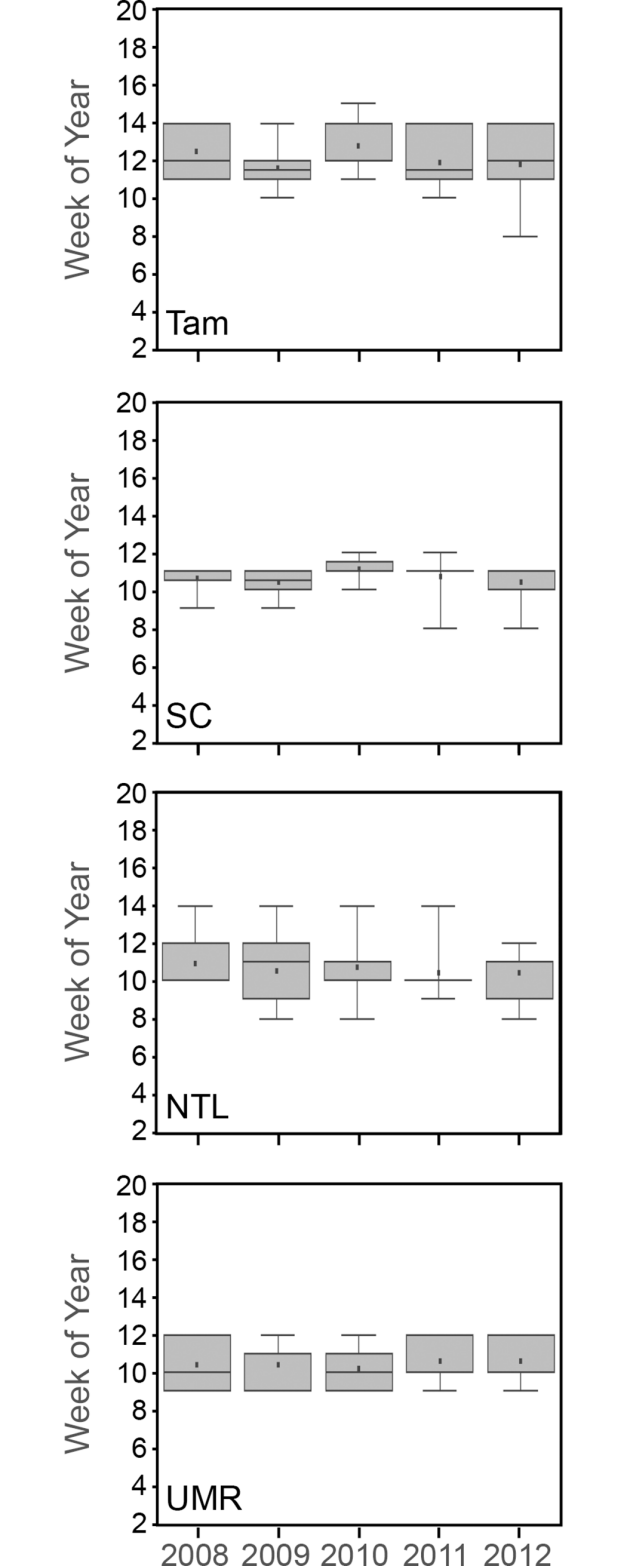
The first week that estimated total actual evapotranspiration was greater than 1 mm. Tam = Tamarac National Wildlife Refuge. SC = St. Croix National Scenic Riverway. NTL = North Temperate Lakes Long-term Ecological Research area. UMR = Upper Mississippi River.

**Fig 13:**
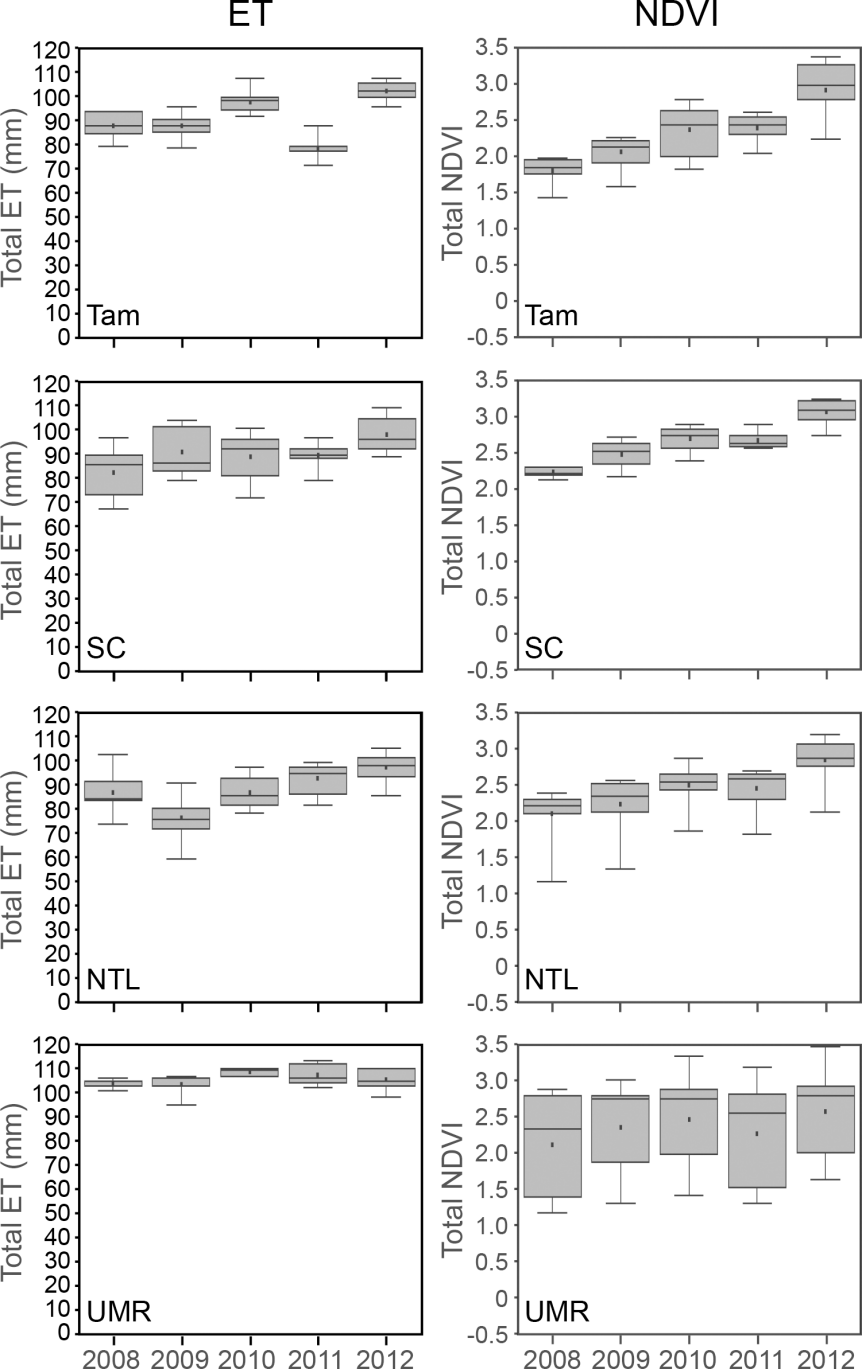
Total estimated actual evapotranspiration (ET) (left panel) and normalized difference vegetation index (NDVI) (right panel) for May. Tam = Tamarac National Wildlife Refuge. SC = St. Croix National Scenic Riverway. NTL = North Temperate Lakes Long-term Ecological Research area. UMR = Upper Mississippi River.

The onset of ET response did not vary annually with the occurrence of the minimum temperature threshold for plant growth (10 ºC) (Fig 12). In fact, the overlap of boxplots along the y-axis in Fig 12 shows little variation in the week of an initial ET response from year to year. However, the total amount of ET we estimated for May revealed early seasonal differences among study areas and years (Fig 13). Total millimeters of ET for May of 2010 in Tam, and May of 2012 in Tam, SC, and NTL, all corresponded well with early pulses of warm air temperatures in those years (Fig 5). The local landscape blocks in the UMR included a large proportion of surface water, which potentially masked any similar temperature-related differences in ET in that area.

Vegetation green-up generally occurred earlier in 2010 and 2012 and later in 2008 and 2011 for local landscape blocks in all four study areas. Green-up typically occurred after snow-off, which usually followed the start of growing conditions. However, time intervals separating these events varied among the four study areas (Fig 10). Green-up closely followed snow-off in the SC blocks, which often was within one or two weeks after temperatures reached the minimum threshold for plant growth. Green-up across SC blocks occurred over a month or more during years when green-up began earlier (2012 was earliest, followed by 2010 and 2009), but was synchronized more closely when minimum temperature thresholds did not occur until April (week 14), as in 2008 and 2011 (Fig 10). Lag times between the occurrences of minimum temperature thresholds, snow-off, and photosynthetic activity varied more across Tam landscape blocks than SC blocks, although green-up was more synchronized across Tam blocks (Fig 10). The timing of when minimum temperature thresholds were met was similar in the SC and NTL study areas, but snow-off and green-up occurred later in the NTL blocks than in the SC blocks (Fig 10). Minimum temperature thresholds typically occurred earlier in the UMR than in the other more northerly study areas, but we often detected the start of green-up later in the UMR (Fig 10). Total NDVI for May generally suggested a trend of increasing production of early green biomass across 2008 to 2012 in the Tam, SC, and NTL (Fig 13).

### 3.3. Water depths and relations to precipitation and amphibian calling and reproductive success

**Fig 14:**
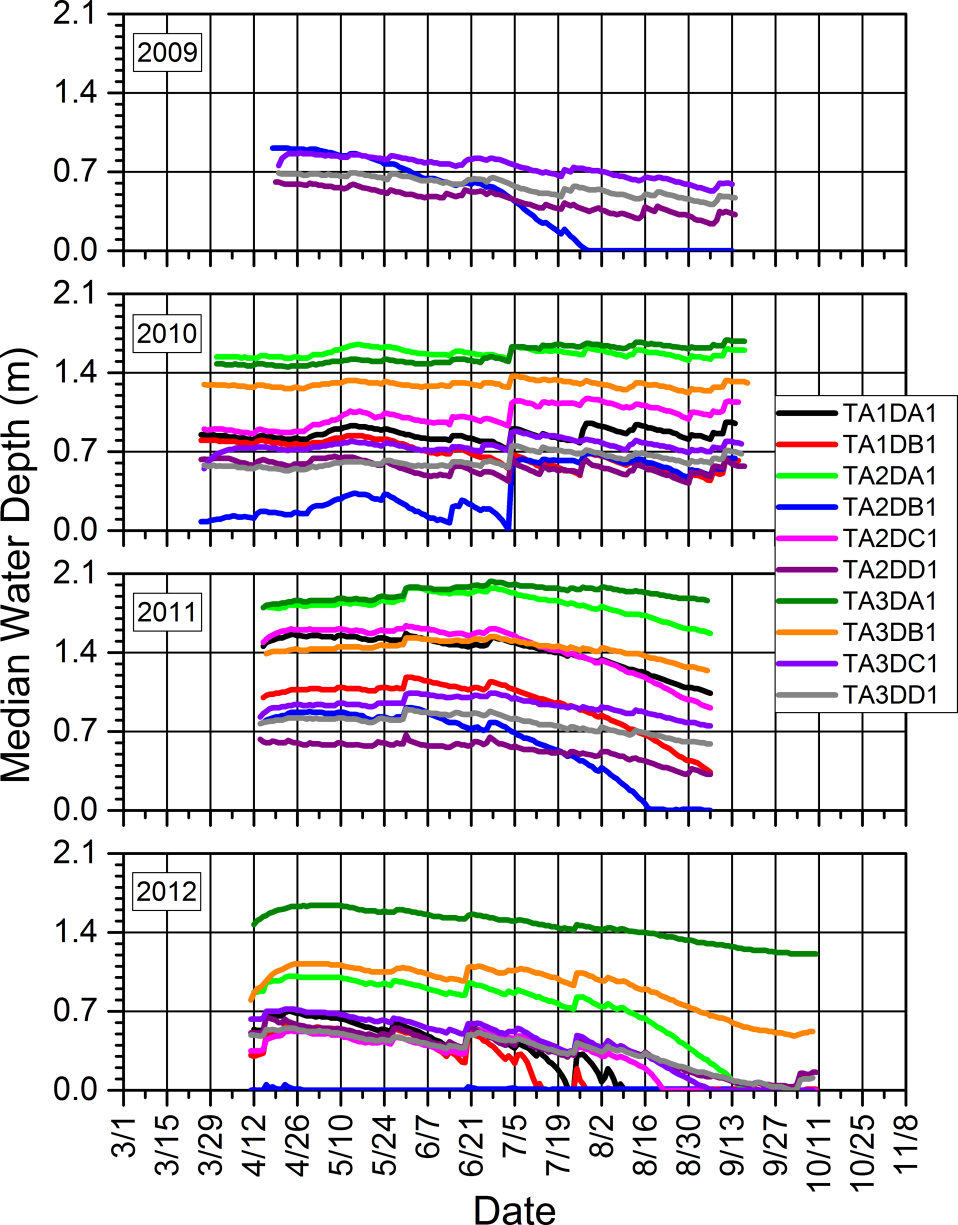
Seasonal median daily water depths for each study wetland in the Tamarac National Wildlife Refuge in which we installed pressure loggers from 2009 to 2012.

**Fig 15:**
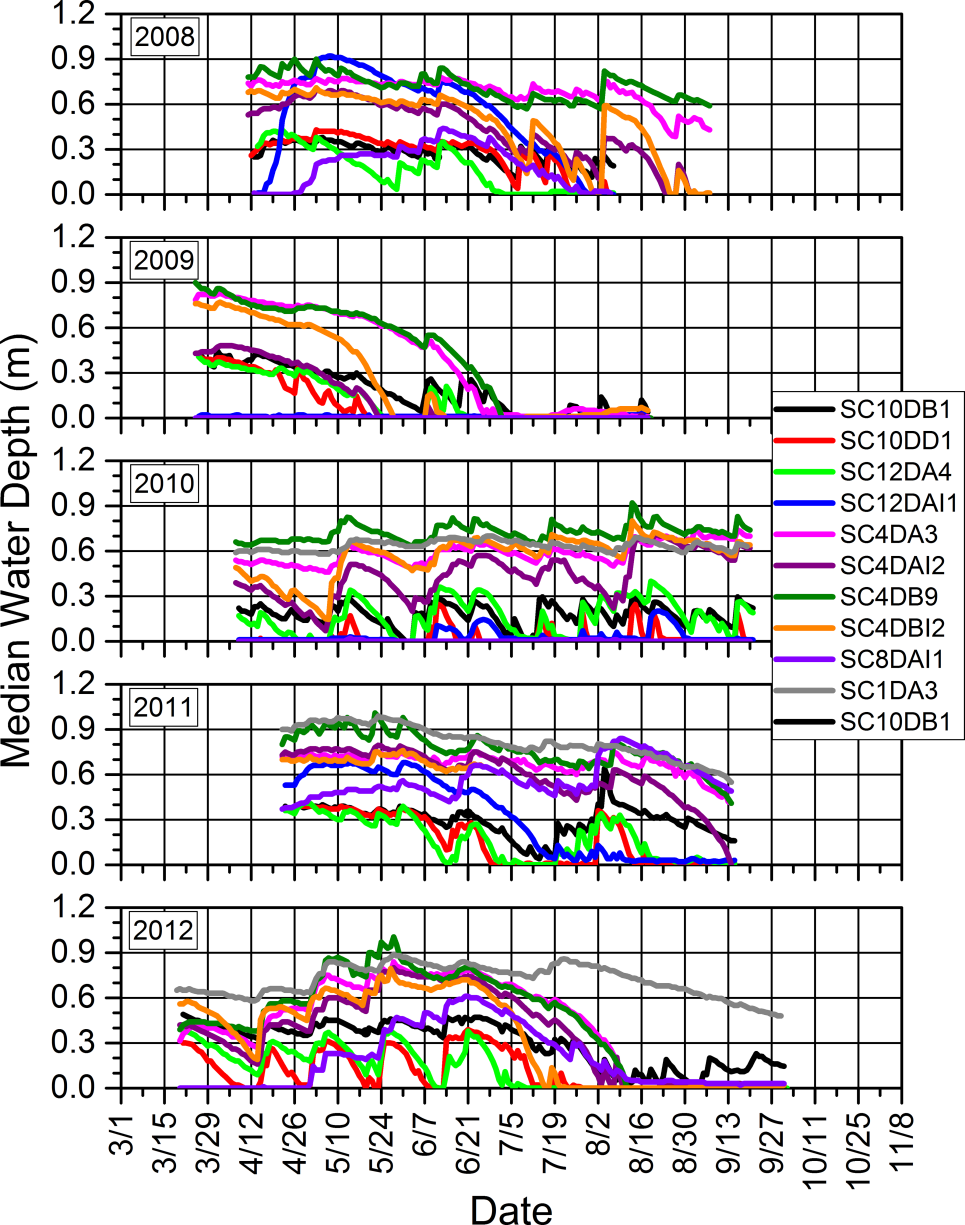
Seasonal median daily water depths for each study wetland in the St. Croix National Scenic Riverway in which we installed pressure loggers from 2008 to 2012. We did not install a logger in SC1DA3 until 2010. The logger at SC12DAI1 failed to record data during 2012.

**Fig 16:**
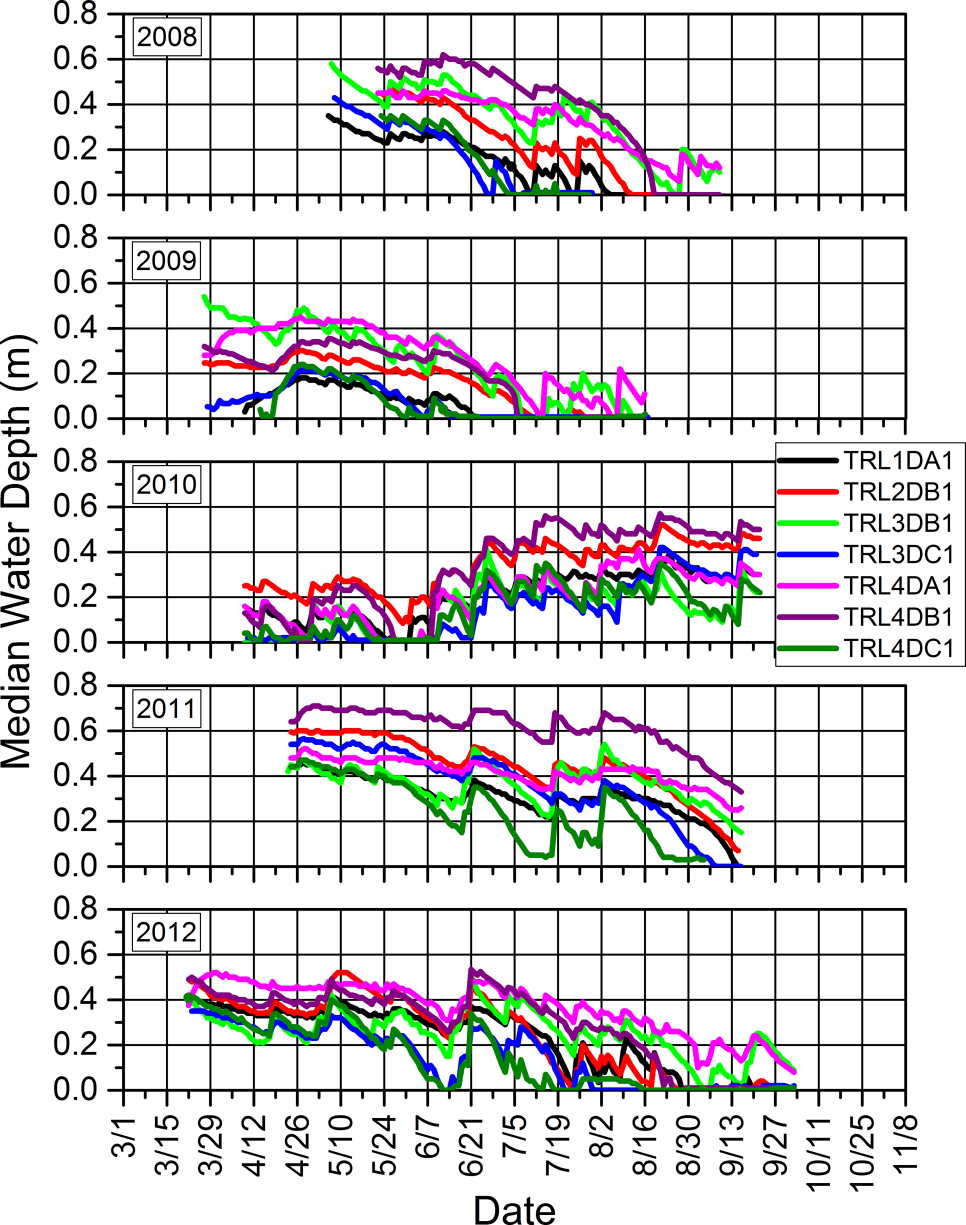
Seasonal median daily water depths for each study wetland in the North Temperate Lakes Long-term Ecological Research area in which we installed pressure loggers from 2008 to 2012.

**Fig 17:**
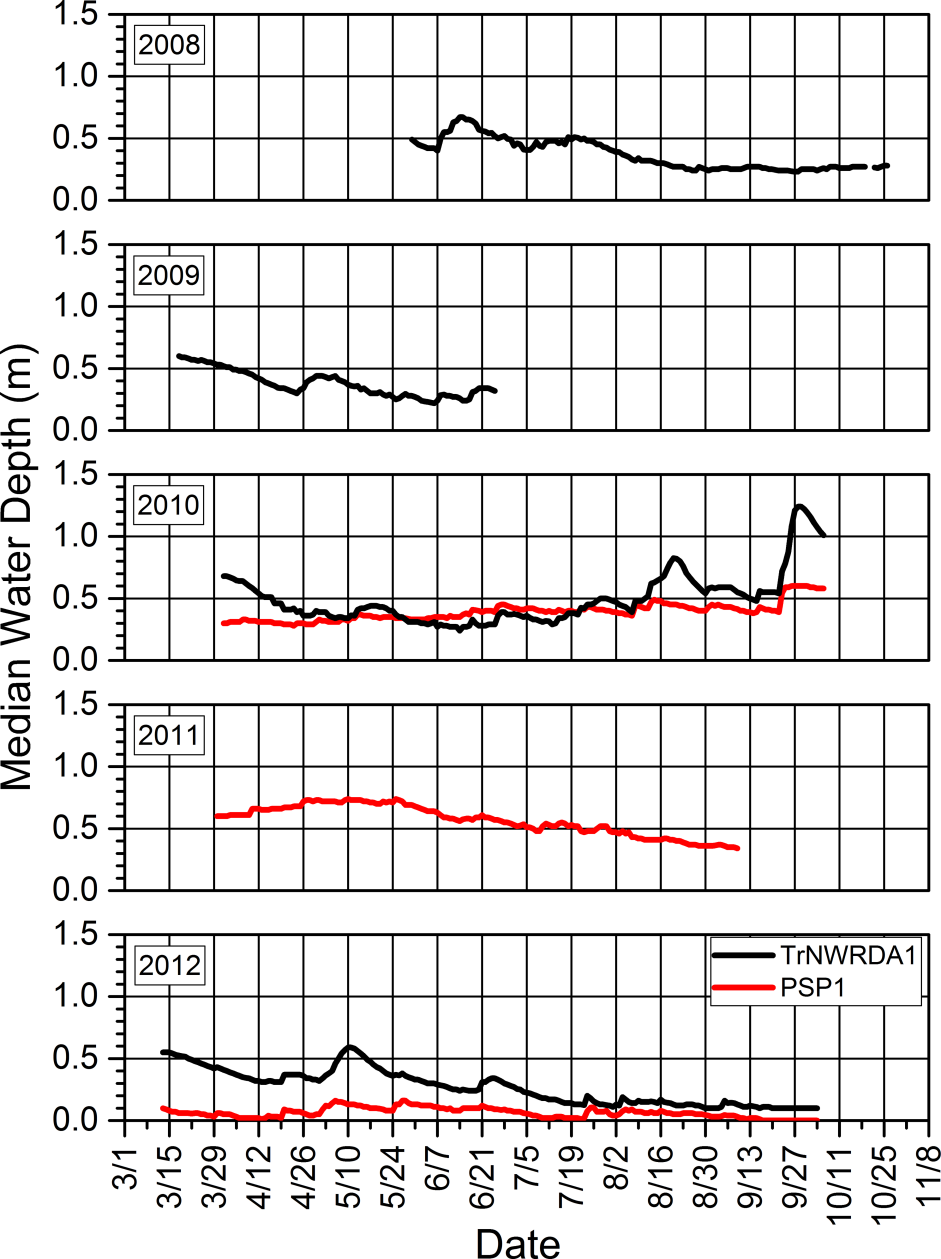
Seasonal median daily water depths for our two study wetlands in the Upper Mississippi River study area in which we installed pressure loggers from 2008 to 2012. We did not install a logger in site PSP1 until 2010. We installed the logger at TrNWRDA1 relatively late in 2008 and removed it relatively early in 2009. It failed to record during the 2011 season.

Seasonal water depths in our study wetlands were dynamic within and across years (Figs 14–17). Our study wetlands in the Tam contained surface water more consistently within and across seasons than those in the other study areas (Fig 14). Several sites in the SC and NTL dried relatively early in 2009 and 2012 and did not rehydrate appreciably, especially in 2009. Some of those sites dried or had low water levels early in 2010 before eventually refilling and then containing surface water longer than during the other years (Figs 15 and 16).

Most daily precipitation totals were low across study areas and years (e.g., S3 Fig). Similarly, most increases in surface-water depth on successive days at SC4DAI2 were small, < 0.05 m, and such daily increases occurred less frequently than daily rainfall events (S3 and S4 Figs).

The numbers of days that daily water depths increased at individual study wetlands across all study areas and years were associated moderately (Spearman’s rank coefficient [SRC] = 0.512) with the numbers of days precipitation was recorded at the nearest weather stations across all areas and years (Table 1; S5 Fig). Similarly, such increases were associated moderately with precipitation at Tam (SRC = 0.498) and the SC sites (SRC = 0.444) when we considered these two study areas separately (Table 1; S6 Fig), whereas associations were stronger for the NTL (SRC = 0.902) and UMR sites (SRC = 0.962; Table 1; S6 Fig).

**Table 1:** Results from Spearman’s rank tests of association (across all years) between the number of days water depths increased at individual study wetlands within years and the number of days when precipitation was recorded at the nearest weather station during the same time period. Tam, SC, NTL, and UMR = the Tamarac National Wildlife Refuge, St. Croix National Scenic Riverway, the North Temperate Lakes Long-term Ecological Research site, and Upper Mississippi River, respectively.

Study Areas	Number of Sites Across Years (Years Data Were Collected)*	Spearman’s Rank Coefficient	P-value
Tam, SC, NTL, and UMR collectively	123 (2008−2012)	0.512	1.61^−9^
Tam	34 (2009−2012)	0.498	2.74^−3^
SC	47 (2008−2012)	0.444	1.75^−3^
NTL	35 (2008−2012)	0.902	1.44^−13^
UMR	7 (2008−2012)	0.962	5.15^−4^

* The years we deployed depth loggers at individual study wetlands varied within study areas.

Variability in the number of days that water depths increased was most pronounced among study wetlands in the SC and least pronounced in the NTL (S7 Fig). Also, some individual wetlands in Tam, the SC, and the UMR stood out as being particularly variable across seasons relative to other wetlands within the same study area (S7 Fig).

**Fig 18:**
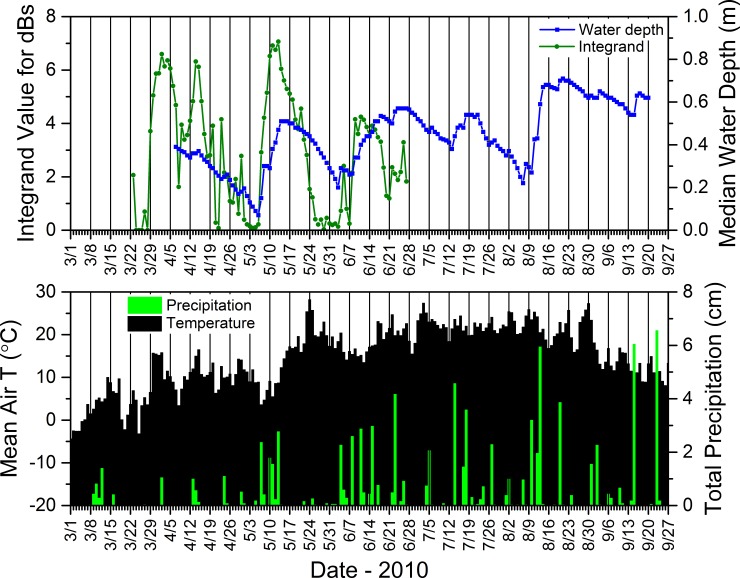
Mean daily air temperature, total precipitation, calling activity for *Pseudacris crucifer* (represented by the integrand value), and median water depth at site SC4DAI2 in the St. Croix National Scenic Riverway during 2010. See S1 Table for information regarding the source of the weather data.

These results, based upon a simple method we used to compare changes in depth to precipitation, generally supported our consistent observations that water-depth dynamics in our study wetlands typically reflected the quantity and distribution of daily rainfall over the course of a season, as evidenced in data for site SC4DAI2 in the SC from 2010 (Fig 18). This example shows that regular, substantial rainfall coincided with more consistent water levels (and, in turn, more consistent calling by *P*. *crucifer*) and ultimately a long hydroperiod (Figs 15 and 18). Also, relatively voluminous rain events often were associated with reversals of depth trajectories that had been descending towards site desiccation (Fig 18). In contrast, small daily rainfall totals did not necessarily result in increases in water depths.

### 3.4. Site occupancy

Calling males of *P*. *crucifer* occupied our study wetlands at consistently high levels. Median occupancy frequencies were 1, 0.8, 1, and 1 across individual study sites in the Tam, SC, NTL, and UMR, respectively, for 2008 to 2012 (Table 2). Sites where we did not detect them in a given year all were either dry, nearly dry, or had intermittent and very limited surface water during this species’ calling period.

**Table 2:** The proportion of study sites with *Pseudacris crucifer* present per year, and the median relative frequency of sites with *P*. *crucifer* present across all years, for each study area. Sites in the St. Croix National Scenic Riverway where *P*. *crucifer* was not present in a given year also contained little or no surface water that year during the typical breeding interval for *P*. *crucifer*.

Study Area	Year	Proportion of study sites with *P*. *crucifer* present
Tamarac National Wildlife Refuge	2009	10/10
	2010	10/10
	2011	10/10
	2012	8/9
	Median relative frequency 2009 to 2012 = 1	
St. Croix National Scenic Riverway	2008	7/9
	2009	8/10
	2010	8/10
	2011	9/10
	2012	10/10
	Median relative frequency 2008 to 2012 = 0.8	
North Temperate Lakes Long-term Ecological Research area	2008	10/10
	2009	10/10
	2010	10/10
	2011	10/10
	2012	10/10
	Median relative frequency 2008 to 2012 = 1	
Upper Mississippi River	2008	1/1
	2009	2/2
	2010	6/6
	2011	6/6
	2012	6/6
	Median relative frequency 2008 to 2012 = 1	

### 3.5. Evapotranspiration

Our ET estimates were strongly associated with GDU (growing degree units) and moderately to strongly associated with precipitation in all four study areas, and the strength of these associations increased with longer time intervals (cumulative to-date > four-week > weekly) (Table 3). These associations were highly dependent upon the temporal autocorrelation inherent in the sequential weekly observations. We tested this via a correlation analysis between ET and the amount of change in weekly GDU (rather than the weekly GDU values themselves), or change in weekly precipitation, and found no evidence of meaningful correlations (Spearman correlation = 0.1566 for GDU and 0.0193 for precipitation). This makes sense because the relative influences of changes in temperature and precipitation on plant growth are not constant throughout the growing season, given changes in soil conditions, leaf area, plant metabolic activity, and day length, among other factors. We therefore did not attempt to statistically minimize temporal autocorrelation in our analyses and acknowledge its role in relation to ET.

**Table 3:** Correlation of estimated evapotranspiration (ET) and Normalized Difference Vegetation Index (NDVI) with growing degree units (GDU) and precipitation. We calculated GDU and precipitation from weather-station data and ET and NDVI from landscape blocks containing field sites. “Weekly total” time interval is the response value for ET or NDVI for each week and the corresponding weekly total precipitation and total GDU. “Four-week total” is the sum of the weekly values over a four-week period incremented a week at time. “To-date total” is a running total that is cumulated each week over the growing season each year. The number of weekly observations available (n) for the correlation analyses is the product of the number of field sites, number of years of observation, and number of composite periods from January through August (34 weeks for NDVI or 30 weeks for ET). All correlations have p < 0.0001. Tam = Tamarac National Wildlife Refuge. SC = St. Croix National Scenic Riverway. NTL = North Temperate Lakes Long-term Ecological Research area. UMR = Upper Mississippi River.

			Correlation with comparable time interval of growing degree units	Correlation with comparable time interval of total precipitation
Node	Variable	Time interval	Spearman’s rank sum	Spearman’s rank sum
Data pooled across nodes	ET	Weekly total (n = 5400)	0.92	0.50
		Four-week total	0.93	0.76
		To-date total	0.96	0.95
	NDVI	weekly total (n = 6120)	0.87	0.46
		Four-week total	0.89	0.69
		To-date total	0.96	0.92
Tam	ET	Weekly total (n = 1500)	0.92	0.54
		Four-week total	0.93	0.81
		To-date total	0.96	0.96
	NDVI	Weekly total (n = 1700)	0.92	0.48
		Four-week total	0.94	0.77
		To-date total	0.95	0.93
SC	ET	Weekly total (n = 1500)	0.93	0.52
		Four-week total	0.94	0.76
		To-date total	0.97	0.95
	NDVI	Weekly total (n = 1700)	0.90	0.51
		Four-week total	0.93	0.73
		To-date total	0.95	0.92
NTL	ET	Weekly total (n = 1500)	0.92	0.54
		Four-week total	0.93	0.78
		To-date total	0.97	0.96
	NDVI	Weekly total (n = 1700)	0.87	0.49
		Four-week total	0.90	0.70
		To-date total	0.97	0.93
UMR	ET	Weekly total (n = 900)	0.92	0.37
		Four-week total	0.93	0.65
		To-date total	0.97	0.96
	NDVI	Weekly total (n = 1020)	0.87	0.38
		Four-week total	0.89	0.59
		To-date total	0.97	0.92

**Fig 19:**
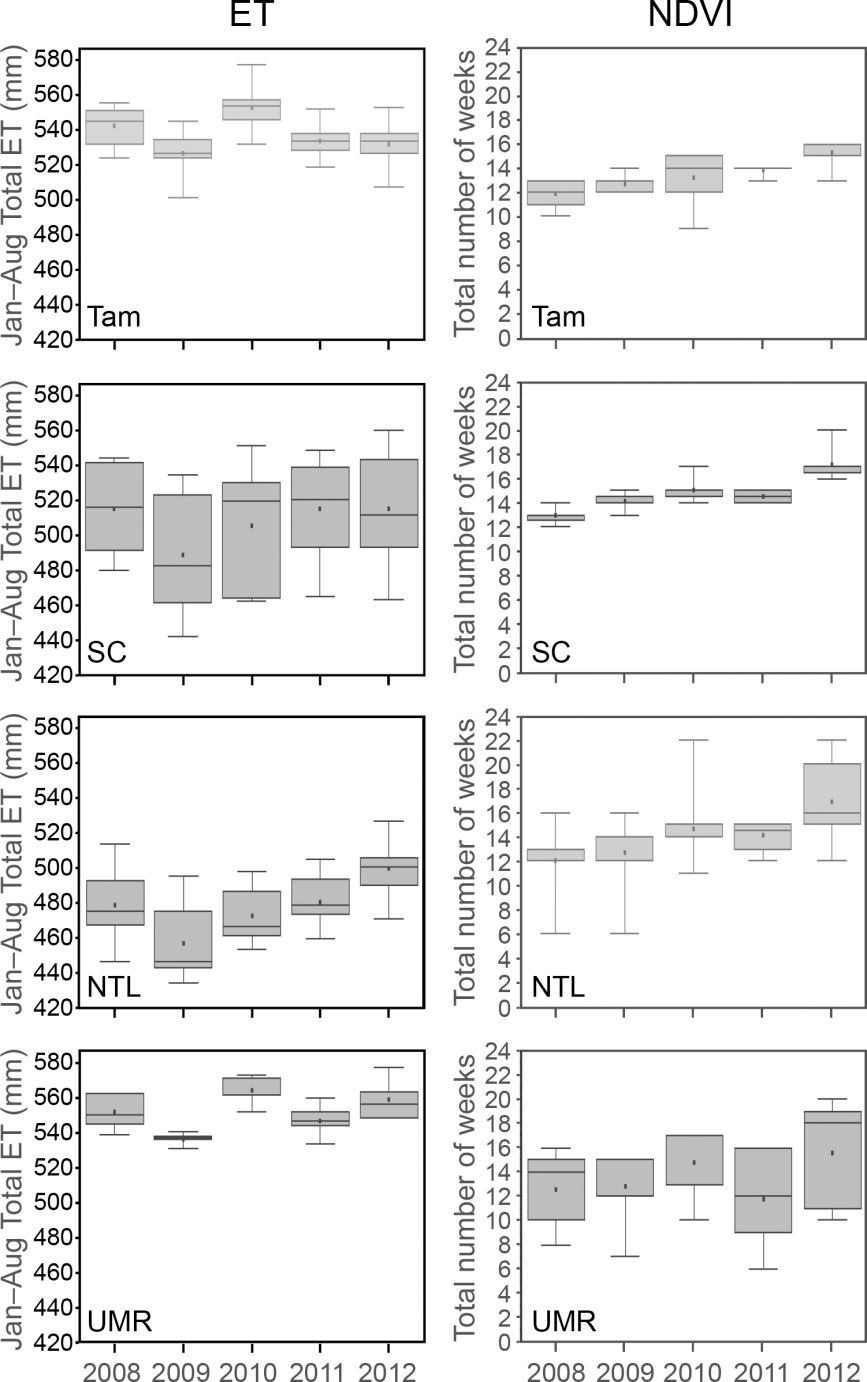
Total seasonal estimated actual evapotranspiration (ET) (left panel) and number of weeks the normalized difference vegetation index (NDVI) (right panel) was above 0.60. Tam = Tamarac National Wildlife Refuge. SC = St. Croix National Scenic Riverway. NTL = North Temperate Lakes Long-term Ecological Research area. UMR = Upper Mississippi River.

According to the climatographs we constructed for Tam (S8 Fig), water was most available during the 2008 and 2010 growing seasons, whereas it was more limited at times during the other seasons. Our results for total estimated ET (Fig 19) reflected these conditions. The early warm temperatures in 2012 overlapped with a period of low water availability, likely moderating total ET for that season. The lowest total ET occurred in 2009, when air temperatures were the coolest among the five years and water availability was limited (S8 Fig).

Total-season ET varied the most across local landscape blocks in the SC compared with other study areas (Fig 19), but so did weather conditions (S9–S13 Figs) recorded at the various weather stations near these landscape blocks that were scattered throughout this long, linear study area (Fig 1). All SC climatographs showed cool, dry conditions during 2009, however, which were reflected in the estimated ET for SC that year (Fig 19). Differences in total ET across NTL blocks were more distinct than for the SC blocks across years and were the lowest among our study areas. The drier, cooler conditions during 2009 (S14 and S15 Figs) also corresponded with the lowest seasonal ET rates in the NTL. Total NTL ET was highest in 2012 (Fig 19), the season with the warmest air temperatures and precipitation near the long-term average (Fig 7). Total-season ET estimates for the UMR varied across years, with the highest rates occurring in 2010, the wettest year, and the lowest rates again occurring during the relatively cool, dry season of 2009 (Figs 7 and 19 and S16–S19 Figs).

### 3.6. NDVI

Our estimates for NDVI also were strongly associated with GDU and precipitation in all four study areas, and the strength of these associations increased with longer time intervals (cumulative to-date > four-week > weekly) (Table 3). As with ET (§ 3.5), these associations were dependent on the temporal autocorrelation inherent in the sequential values. We found no meaningful correlations between NDVI and GDU or precipitation when we controlled for temporal autocorrelation by analyzing only the amount of weekly change in these variables (Spearman correlation = 0.1442 for weekly change in GDU and 0.0150 for weekly change in precipitation). This makes sense for similar reasons we explained for ET and therefore we did not attempt to statistically minimize temporal autocorrelation in our analyses and acknowledge its role in relation to NDVI.

During 2010 and 2012, growing conditions began earlier due to warmer air temperatures, which enabled leaf-out over longer portions of each growing season (Fig 19). Conversely, colder temperatures and later snowmelt in the other years, especially in 2008 (Fig 10), delayed the onset of growing conditions and reduced the number of weeks for leaf-out (Fig 19). The number of weeks when NDVI values were ≥ 0.60 (the winter background level) within each year varied the most across local landscape blocks in the UMR and the least among blocks in the SC (Fig 19). In contrast, UMR blocks varied the least in total-season ET, whereas SC blocks varied the most (Fig 19).

## 4. Discussion

We collected data from satellite- and ground-based sensors and integrated them across spatial and temporal scales for this assessment. Our results describe the nature of historical and recent temperature and precipitation dynamics, the extent to which recent dynamics were related to variability in key ecological conditions and processes across interconnected wetlands and uplands, and potential evidence for or against meaningful climate-related changes in the ecological variables we measured in our four study areas.

### 4.1. Temperature

#### 4.1.1. Relevance of historical and recent dynamics

Since the mid-1970s, mean daily temperatures for March through August mostly were warmer than the historical means in the climate divisions encompassing each of our study areas (Fig 4). This set of mostly warmer seasons was similar to what Winkler et al. [83] described for the midwestern United States, although they also described warming dating back to about 1900. Included among the warmer seasons since the mid-1970s were several individual seasons that ranked among the warmest since 1895, including extremes in 2010 and 2012 (Fig 4). The frequency and magnitude of these warmer seasons overall suggests that temperature-induced ecological changes might have been occurring in our study areas over a prolonged period prior to when we began this research in 2008.

These seasonal temperature averages were useful as coarse indicators of relative seasonal temperatures and variation across the years of this study and, considered alone, suggested the potential for different ecological dynamics across seasons. However, these coarse indicators masked finer-scaled temperature differences within and across seasons that were important to understand in terms of the ecological relations we assessed. This was evident when we used daily temperature data obtained from individual weather stations closest to our study wetlands to assess within-season temperature dynamics in relation to the ecological variables we measured daily or over weekly or eight-day intervals from 2008 to 2012. These within-season dynamics illustrated the nature and extent of temperature variation and the ways it related to variation in our measures of the key ecological indicators we targeted. For example, mean daily temperatures throughout the 2010, 2011, and 2012 seasons overall were higher than they were during the 2008 and 2009 seasons in each study area (Fig 5). Furthermore, mean daily temperatures were greater during the earliest days of the 2010 and 2012 seasons than for the same time period in 2008, 2009, and 2011 (Fig 5). Notably, these early-season temperatures were the most apparent differences in the temperature profile for the 2011 season relative to the profiles for the 2010 and 2012 seasons (Fig 5). Such differences, evident among these intra-seasonal profiles, show what Ault et al. [84] described as the false spring of 2012 across North America, in that abnormally early warm temperatures were not continuous after the initial surge. Thus, the consistency and timing of warmer daily temperatures played nuanced roles in differences among seasons, with warmer days early in the season contributing substantially to the historically warm rankings of the 2010 and 2012 seasonal averages. In turn, these varying, nuanced temperature dynamics were associated with ecological differences we observed within and across seasons, such as those pertaining to phenological responses (Figs 9 and 10).

#### 4.1.2. Relations of the timing of annual cyclo-seasonal events to temperature from 2008 to 2012

**Fig 20:**
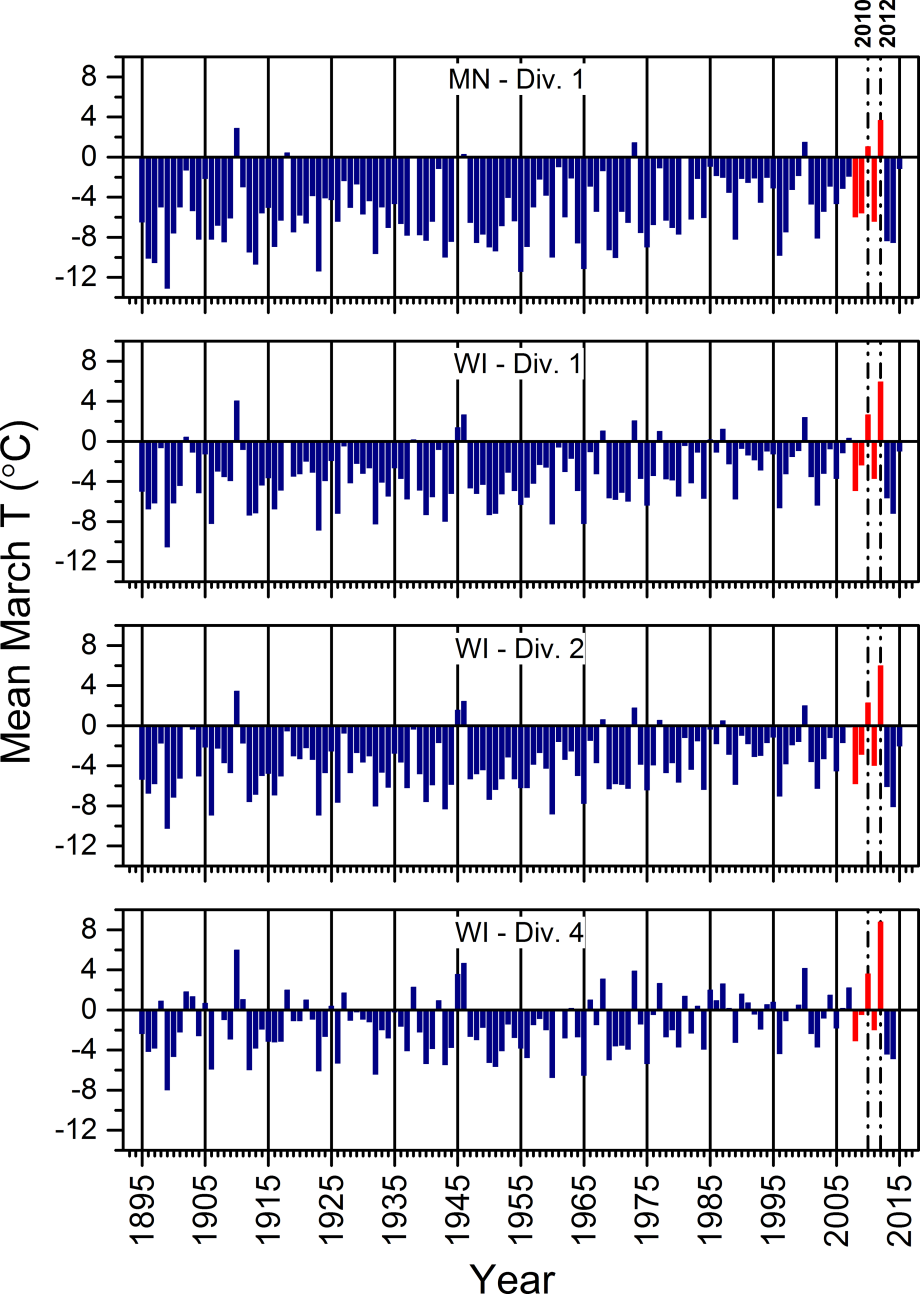
Mean March temperatures for the climate divisions that included our study areas. The Tamarac National Wildlife Refuge, St. Croix National Scenic Riverway, North Temperate Lakes Long-term Ecological Research area, and Upper Mississippi River were in MN-Div. 1, WI-Div. 1, WI-Div. 2, and WI-Div. 4, respectively. Red bars indicate the years of our field study (2008 to 2012). See S1 Table for information regarding the source of the climate data.

Our observations of temperatures in 2010 and 2012 suggested that other relatively warm seasons prior to 2008 (e.g., 2006 − Fig 4) also could have included relatively early and rapid transitions from winter to spring conditions. However, historical mean March temperatures across these four climate divisions suggest otherwise, as such temperatures generally were warmer after about 1980, but largely were well below the mean for March of 2012 and, to a lesser extent for March of 2010 (except somewhat in the UMR; Fig 20). This further illustrates how rare the 2010 and 2012 seasons were in our study areas, even among seasons that typically have been warmer in recent decades, how intra-seasonal temperature dynamics varied across years, and how evaluating or predicting the effects of temperature on ecological conditions and processes based upon temperature conditions summarized at broad temporal scales alone, even only at the seasonal scale, could be misleading.

Finer-scaled information also was valuable regarding the onset of amphibian calling relative to temperature conditions each year. Our intensive daily sampling schedule using acoustic recorders (five-minute recordings at the top of every hour) and our analytic approach provided uniquely detailed information regarding the first amphibian calls of the season. This information was more reliable for assessing the first amphibian calls of the season relative to environmental conditions than information we could have obtained via less intensive daily sampling schedules or human aural surveys that were more limited temporally or in frequency (e.g., [85], [86]). This was true even if we had used other sampling methods or schedules from before any calling began, because first calls often occurred during only one or a limited number of samples during a 24-h period and often during daytime hours. Using the acoustic recorders proved to be highly effective and efficient.

We did not observe amphibian calls at any study wetland before the average air temperature for an eight-day interval was at least 0 ºC (Fig 9). Thus, the integrands of mean daily temperatures we derived for such intervals were useful to indicate when temperatures had warmed sufficiently to initiate transitions from winter to more spring-like conditions. In contrast, snow was not necessarily gone from local landscape blocks before amphibians first began calling (Fig 9). This shows how early breeding species in the north-central United States can overwinter at or near breeding wetlands and/or go around or across patches of snow when moving overland to nearby breeding wetlands and that these wetlands can contain ice-free water despite snow remaining on the surrounding landscape (pers. obs.). It also suggests that snow cover alone might not be a useful indicator for when to begin call surveys designed to assess calling phenology or for modeling past phenology based upon historical climate records. We are not aware of other similarly detailed assessments of relations between temperature, snow cover, and amphibian calling activity, but our results based upon these measurements indicate their potential value for long-term studies to determine how future changes in early season temperatures and/or late-winter snowfall could affect the timing of the start of biological activity in our study areas.

Not surprisingly, the dramatic temperature increases in early March of 2010 and, especially, 2012 (Fig 5) largely resulted in snow melting, amphibians calling, and photosynthetic activity occurring noticeably earlier than during 2008, 2009, and 2011 in all four study areas (Fig 9). Notably, amphibians (*P*. *crucifer*, *P*. *maculata*, and/or *L*. *sylvaticus*, depending upon the site) began calling in large numbers early and quickly at individual wetlands in each study area in 2012 in particular, as rising temperatures quickly melted snow and ice from wetlands and surrounding uplands and exceeded requisite physiological thresholds for these species to become fully active (Fig 9). This rapid physical transformation and onset of intense amphibian activity was remarkably unusual in our experience. Furthermore, these events were abrupt and rapid across study areas. As a result, we observed considerably less temporal separation between these events among study areas than was more typical based upon the locations of these areas along latitudinal and longitudinal climate gradients (Figs 1 and 9).

Although results from this five-year interval of collecting field data did not allow us to assess any long-term trends in amphibian-calling phenology, our observations in general were similar to other reports that earlier or later amphibian calling/breeding activity was related to warmer seasonal temperatures (e.g., summarized in [10], [85–87]). Based upon our aforementioned assessment of historical seasonal temperatures that indicated mostly warmer seasons since the mid-1970s or mid-1980s (Fig 4), one might speculate that amphibians called earlier more frequently in these areas after the mid-1970s or mid-1980s than before. However, March temperatures for the same period largely were below the historical mean (Fig 20), which suggests otherwise.

Similar to the start of amphibian calling at individual wetlands, we observed the onset of photosynthesis in local landscape blocks earliest in 2012, followed by 2010 (Fig 10). Our observations in 2012 corroborated those of Ault et al. [84], who reported widespread early leaf-out in this and other regions of the country in relation to abnormally high late-winter temperatures. We detected photosynthetic activity during the same week across all four of our study areas in 2012 (Fig 10), which, similar to the start of amphibian calling, reflected the pervasiveness of abnormally high temperatures throughout the region. The ET product we used showed little sensitivity to the specific timing of the start of the growing season (likely because these data originally were developed to estimate consumptive water use and drought on agricultural lands [51], [88] and our sites were in non-agricultural, humid temperate landscapes, suggesting we might need a different parametrization of the SSEBop model [51]). Early season monthly ET totals, however, did reflect the warmer temperatures in 2010 and 2012 in the Tam blocks, but interpreting ET responses for 2010 relative to other years was less straightforward for the other study areas (Fig 13). This might have been related to insufficient availability of surface water to meet ET demands. For example, the onset of ET in the SC and NTL blocks did not appear to be related to early warm temperatures in 2010 (Fig 13), but early season precipitation was low in those areas during 2010 (S9–S15 Figs). Limited precipitation during 2009 also could have contributed to dry conditions at the beginning of the 2010 season (Figs 14 and 15 and S9–S15 Figs). Thus, the interplay of temperature, water availability, and the scale at which we took measurements was important for evaluating when biological activity increased beyond winter baselines in our study areas.

Notably, several continent-wide analyses of satellite-derived NDVI data, extending as far back as 1981 and through the years prior to 2008, did not identify any trends in vegetation phenology near our study areas [89–92], with the possible exception of a study by Zhang et al. [93] that suggested a slight delay in the start of the season from 1982 to 2005, based upon coarse spatial-resolution data. These continental studies assessing trends are informative, but outcomes clearly can mask the potential importance of inter- and intra-annual variation in the timing of cyclo-seasonal events, which can indicate impacts on ecological processes and conditions on the ground, from amphibian reproduction in individual wetlands to primary productivity and water availability across the broader wetland-upland landscape matrix.

Several phenomena could make it difficult to identify the onset of photosynthetic activity based upon remotely sensed data. For example, late-winter NDVI values can increase for individual pixels when melting snow exposes evergreen foliage [94], which can result in multiple false green-up events in association with recurring cycles of snowfall and melting [95]. Conversely, the presence of surface water can depress NDVI values (water absorbs energy in the wavebands used to derive NDVI) even when leaf-out is underway. Many approaches have been developed to capture the start of the growing season using remotely sensed data, but no single approach has been shown to be effective for all environmental settings [96]. Given this, and that projected climate trends indicate warmer winters for the midwestern United States [97], detecting the true onset of photosynthetic activity could be increasingly difficult if the frequency of late-winter snowfall/snowmelt cycles increases.

Our weekly ET and NDVI data, averaged across 4-km^2^ local landscape blocks, were spatially and temporally coarse relative to the acoustic and water-level data we collected hourly at individual wetlands within those blocks. Considered together, however, all these data provided important complementary ecological information for our assessment that was notably richer than the information we obtained more simply at one scale or the other.

**4.1.2.1. Disconnectedness between the timing of the start of season and the timing of events later in the season.** Results from numerous studies of various taxa have described earlier seasonal starts to biological activity due to warmer temperatures (e.g., [10], [35], [98–100]), including for the north-central United States [15]. Not surprisingly, determining the actual ecological ramifications of earlier starts of biological activity remains more challenging (e.g., [99], [100]). Hypothetically, the relatively early starts of the 2010 and 2012 seasons we observed could have affected the individual fitness of amphibians and other species in our study areas differently than later starts did in 2008, 2009, and 2011. For example, increased temperatures could have affected individuals physiologically and altered breeding behavior or development rates, resulting in changes in the timing of trophic interactions or other cascading effects (e.g., [35], [99]).

We did not attempt to measure any such direct or indirect effects. However, our results from analyzing data from a subset of sites as a case study showed that phenophases and the timing of within-season peaks in calling activity for *P*. *crucifer* in 2010 and 2012 were not related consistently to the timing of *P*. *crucifer’s* first calls in those seasons (Fig 11). These results suggest that assuming or predicting ecological outcomes later in a season based upon the timing of the start of season alone could be problematic. Furthermore, the first calls of *H*. *chrysoscelis*/*versicolor*, which typically occurred in May, were earlier in 2012 at four of the five sites we analyzed, but did not appear related to the timing of the relatively early start of the season in 2010 (Fig 11).

**Fig 21:**
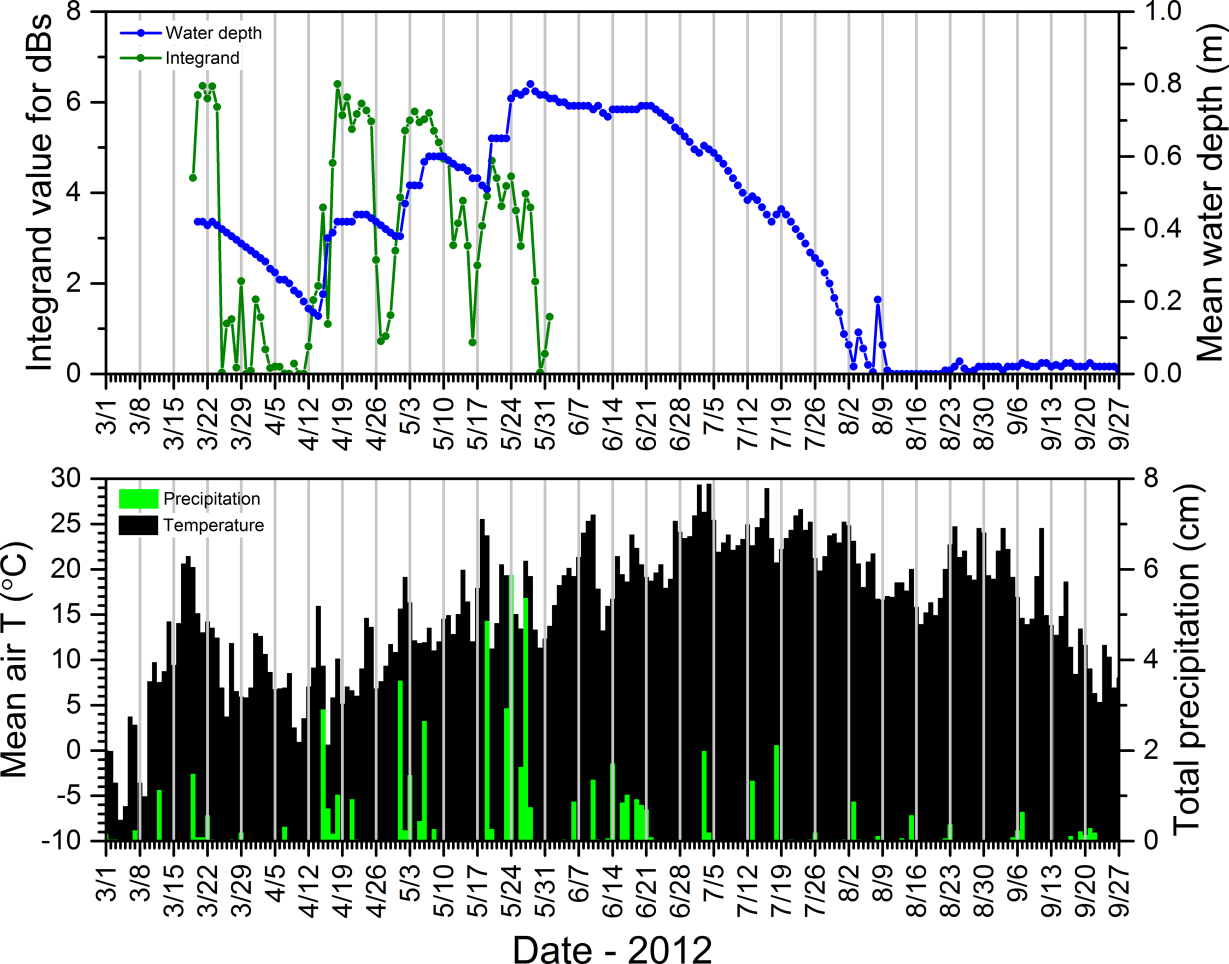
Mean daily air temperature, total precipitation, calling activity for *Pseudacris crucifer* (represented by the integrand value), and median water depth at site SC4DAI2 in the St. Croix National Scenic Riverway during 2012. See S2 Table for information regarding the source of the weather data.

Intra-seasonal temperature and precipitation dynamics appeared to have played roles in these differences. For example, temperatures turned colder after the initial warm surges in March of 2010 (less so in the UMR) and 2012, before increasing again more similarly to rates of increases that occurred in 2008, 2009, and 2011 (Fig 5). These early-season temperature swings co-occurred with changes in calling activity for *P*. *crucifer* after calling had begun (e.g., Figs 18 and 21) and, thus, disrupted continuity in calling activity over the course of the season. In addition, precipitation and related surface-water availability also fluctuated after calling had begun at some sites and were associated with fluctuating calling activity, including in 2010 and 2012 (e.g., Figs 18 and 21). In effect, temperature and precipitation dynamics appear to have strong potential to decouple the timing of calling activity later in a season from when calling first begins for that season, either within or among species. Similarly, the start of photosynthetic activity was associated with early-season temperatures (Fig 10), but subsequent changes in NDVI and ET were related to the interplay of temperature and precipitation over the season. Overall, these observations illustrate the potential complexity of determining if and how observed phenological changes result in effects on individual fitness, populations, or communities, as others have suggested (e.g., [15], [35], [99], [100]) and the ongoing need for research on such relations, including long-term field studies [35]. Our results also suggest the importance of assessing impacts of fluctuating abiotic conditions on related ecological processes at fine spatial and temporal scales within and across seasons.

### 4.2. Precipitation

#### 4.2.1. Historical and recent dynamics and temporal scale

Results from models predicting future precipitation patterns for the north-central United States describe considerable uncertainty [83]. These results project drier conditions in the northwestern part of the region, which includes Tam, but mostly increased, variable precipitation elsewhere with changes in seasonal patterns and more frequent intense events [83]. Seasonal precipitation varied considerably across our study areas from 2008 to 2012, including some marked contrasts between wet and dry seasons (Fig 7). Similar to seasonal averaged daily temperatures, seasonal averaged precipitation totals were useful as broad indicators of the precipitation dynamics across these five seasons relative to precipitation patterns across previous years. Perhaps even more so than with the temperature data, however, our analyses of the intra-seasonal monthly and daily precipitation data, we obtained from the automated weather stations nearest our study sites, complemented the coarser information contained in the seasonal averages in important ways. This primarily was because intra-seasonal distributions of precipitation differentially drove wetland surface-water availability and amphibian calling activity and potential reproductive success at individual wetlands, as well as evapotranspiration and primary productivity across landscape blocks.

The distributions of monthly rainfall totals collected at the weather station nearest to four of our SC study wetlands illustrate this point (Fig 8B), although variations on this theme were apparent for all our study areas (Fig 8A–8D). The averaged seasonal precipitation totals, for weather stations within the climate division containing the SC, indicated the 2009 and 2010 seasons were dry and wet, respectively, relative to the other years of this study and to the historical mean (Fig 7). Importantly, however, individual monthly precipitation totals from the nearest weather station during the drier 2009 season were relatively low until August, whereas monthly totals also were relatively low for March and April of the wetter 2010 season before increasing from May through August, resulting in a greater seasonal total than for 2009 (Fig 8B). Similar to low monthly precipitation totals during 2009, totals during March and April of 2010 also had implications for surface-water availability at some sites and, in turn, amphibian calling activity and reproductive success (Figs 15 and 18; § 4.2.2). However, the averaged seasonal total alone did not suggest precipitation might have been limiting for any wetlands during the 2010 season (Fig 7).

Seasonal precipitation totals (data from the nearest automated weather station) for these same four sites were similar for the 2008, 2011, and 2012 seasons (Fig 8B), suggesting that ecological conditions perhaps were similar across these seasons. However, the totals for individual months were more moderate consistently throughout the 2008 and 2011 seasons, whereas they mostly were lower during 2012, except for May, which had the highest total of any month across all five seasons (Fig 8B). Thus, similar to the seasonal totals averaged across climate-division weather stations described above, seasonal precipitation totals from the individual weather station were only a coarse indicator of potential ecological conditions and effects within a season (Fig 15; § 4.2.2). As with temperature data, using daily data enabled us to evaluate ecological responses to weather dynamics and, by extension, climate in ways that were more informative because they occurred within biologically meaningful time frames, such as those that affect amphibian reproductive activity and success.

These comparisons of the informative value of precipitation data across scales suggest the potential for verisimilitude regarding the effects of climate change on species [101] when using broad-scale indicators of weather or climate conditions as variables in models, as some have reported for amphibians [102–106], and support concerns that finer-scale data could be important [107], as Bateman et al. [108] suggested for evaluating shifts in bird distributions.

#### 4.2.2. Relations of precipitation to surface-water availability and amphibian calling and reproductive success

**Fig 22:**
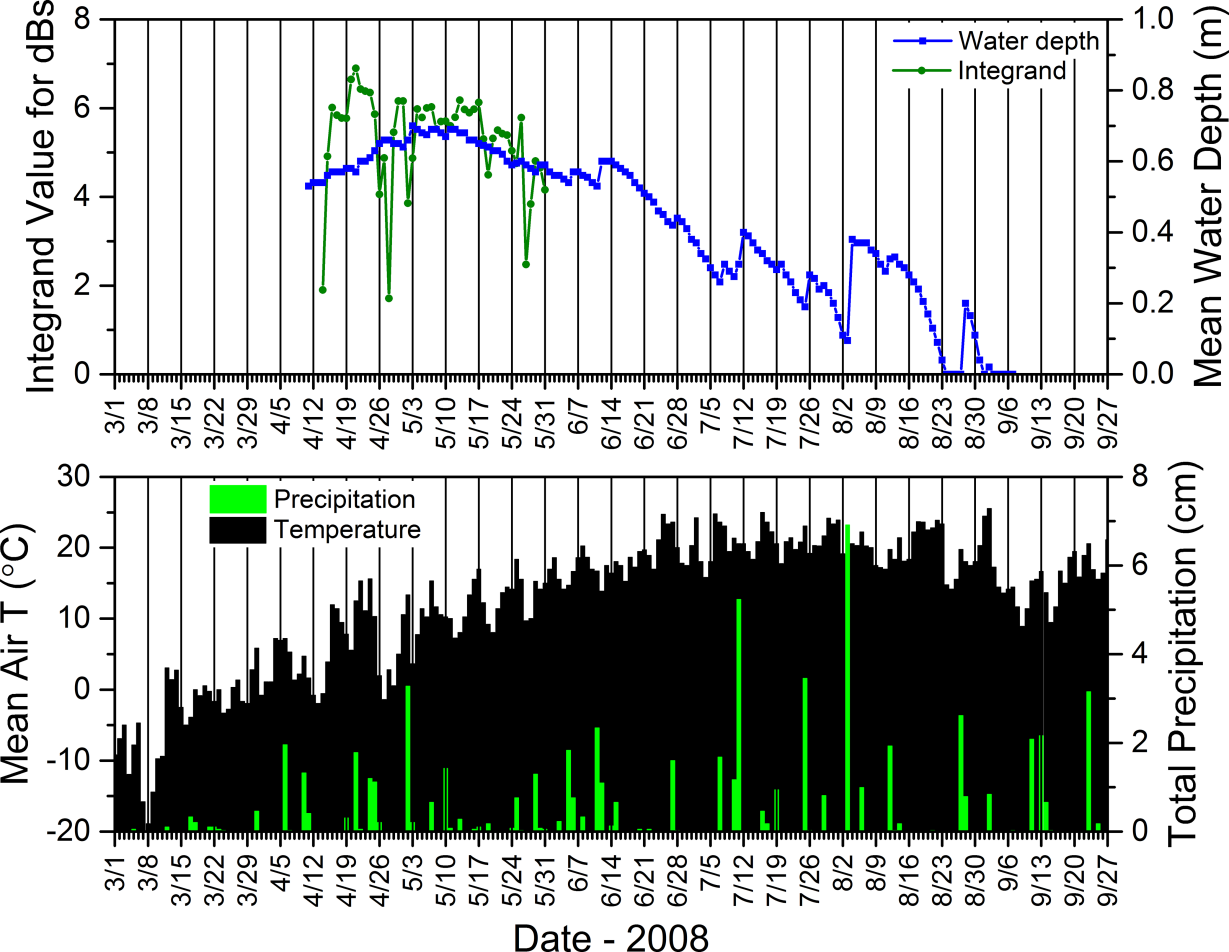
Mean daily air temperature, total precipitation, calling activity for *Pseudacris crucifer* (represented by the integrand value), and median water depth at site SC4DAI2 in the St. Croix National Scenic Riverway during 2008. See S1 Table for information regarding the source of the weather data.

**Fig 23:**
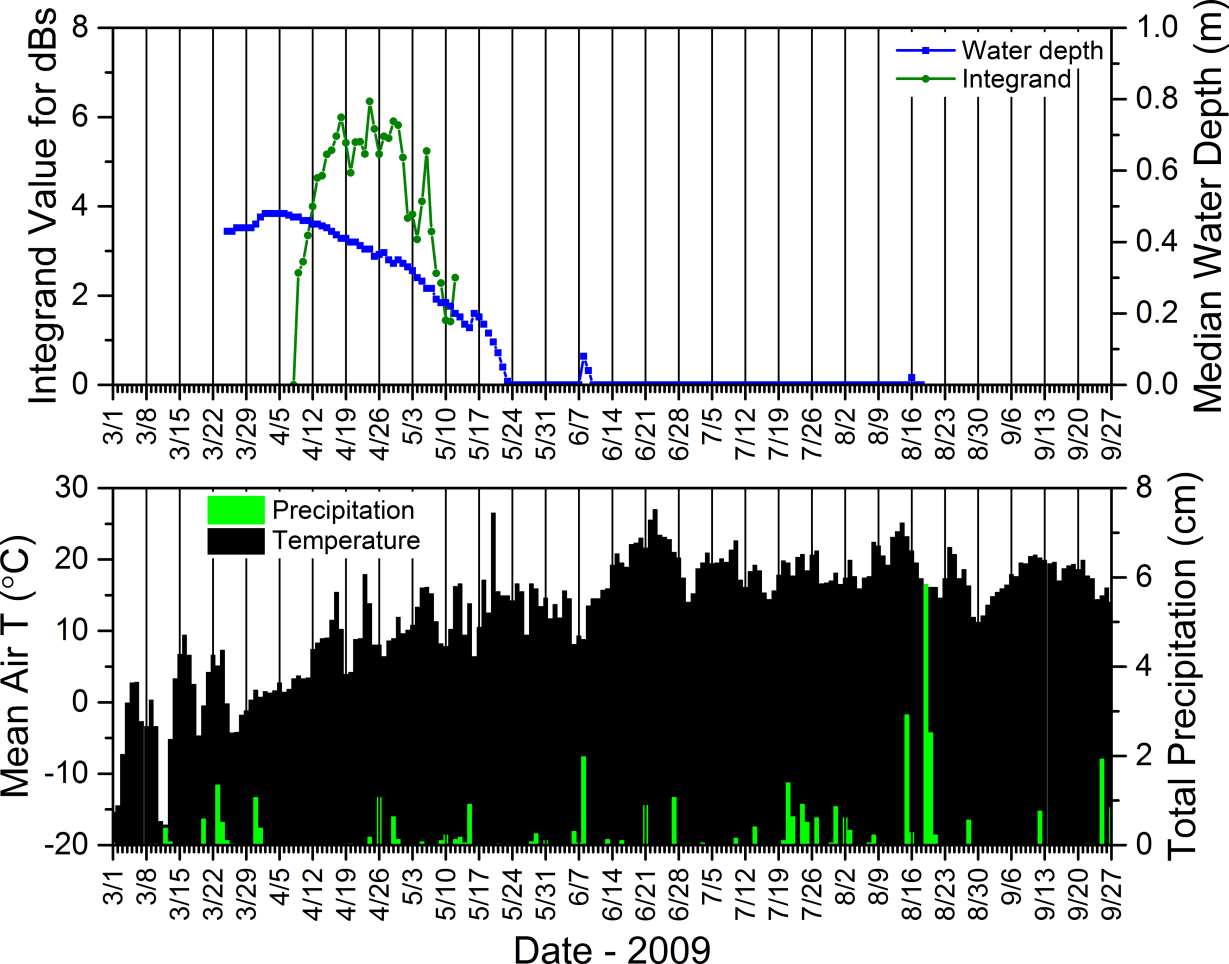
Mean daily air temperature, total precipitation, calling activity for *Pseudacris crucifer* (represented by the integrand value), and median water depth at site SC4DAI2 in the St. Croix National Scenic Riverway during 2009. See S1 Table for information regarding the source of the weather data.

**Fig 24:**
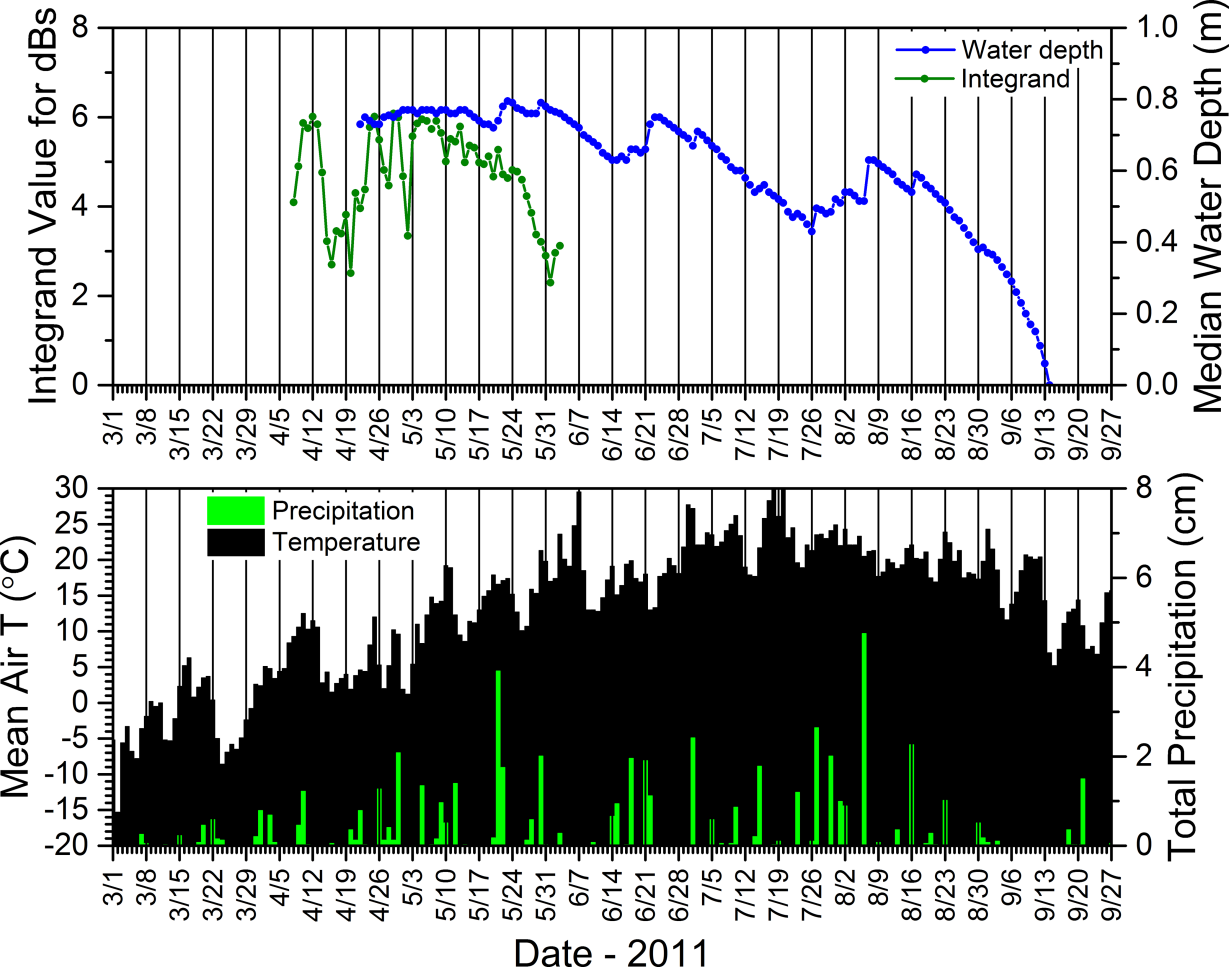
Mean daily air temperature, total precipitation, calling activity for *Pseudacris crucifer* (represented by the integrand value), and median water depth at site SC4DAI2 in the St. Croix National Scenic Riverway during 2011. See S1 Table for information regarding the source of the weather data.

Relations between precipitation and the availability of surface water in palustrine wetlands (*sensu* [109]) can be relatively unambiguous under drought conditions (e.g., [102]; 2009 in Figs 15 and 16). However, a limited number of studies have described what can be more tangled relations between precipitation and wetland surface-water availability under more variable climate conditions (e.g., summaries in [13], [110], [111]). Intra-seasonal rainfall was associated moderately to strongly with increases in concurrent wetland water depth across our study areas (§3.3; Table 1). We did not assess any of the morphologic, edaphic, and vegetation factors, among others [110], [111], in the catchment of each wetland or across the landscape [1], [112] that affect how much, or when, rainfall ends up as surface water in a wetland basin. Similarly, we did not consider the influence of the snowpack, pre-winter wetland water levels, or ground water on water depths at the start of or throughout the season. Thus, the moderate statistical associations we observed in TAM and the SC via our simple comparisons are not surprising. Furthermore, the variability among sites suggested that one or two sites could weigh heavily in affecting the Spearman rank coefficient for a particular study area (S7 Fig). However, our results, based upon a simple method we used to compare changes in depth to precipitation events, supported our general field observations that water-depth dynamics in our study wetlands typically reflected the quantity and distribution of daily rainfall over the course of a season. In addition, the influence of rainfall on water depth and hydroperiod in our study wetlands was unambiguous when we examined fine-scaled temporal data from individual sites in individual years (e.g., Figs 18 and 21–24) and could visually examine intra-seasonal relations in more detail. Regular, substantial rainfall coincided with more consistent water levels. Relatively voluminous rain events often were associated with reversals of depth trajectories that had been descending towards site desiccation, whereas the much more common (S3 Fig) small daily rainfall totals did not necessarily result in measurable increases in water depths (e.g., Figs 18 and 21–24).

In terms of effects on amphibian populations, our data provide details regarding how variable precipitation, water depth, and hydroperiods related to the calling activity of *P*. *crucifer* (as a proxy for reproductive activity) and potential reproductive success of *P*. *crucifer* and other species across seasons. These details demonstrate how variation in the frequency, quantity, and intra-seasonal distribution of precipitation could or did affect fitness among individuals within breeding subpopulations of this species in our study areas. Having such specific information also enables us to draw general conclusions regarding potential reproductive success for *P*. *crucifer* and other amphibian species in these areas under the weather conditions we observed.

As an example of this, water levels and hydroperiods in site SC4DAI2 varied considerably within and across seasons from 2008 to 2012 (Figs 22, 23, 18, 24, and 21, respectively). Water levels were more consistent and hydroperiods were longer when rain fell more frequently and/or in larger quantities, such as during 2008, 2011, and the latter and middle portions of 2010 and 2012 (Figs 22, 24, 18, and 21, respectively). This variation affected *P*. *crucifer* and, presumably, other resident amphibian species (*Anaxyrus americanus*, *H*. *chrysoscelis/versicolor*, *P*. *maculata*, *and L*. *sylvaticus*) in important ways because they require sufficient water levels and hydroperiods to complete the seasonal reproductive sequence of mating, embryonic and larval development, and, ultimately, metamorphosis. Hatching to metamorphosis for *P*. *crucifer* can require up to three months in northern climates depending upon conditions [45], of which the availability of sufficient surface water is most essential. Based upon this requirement, and the timing and consistency of calling activity (during which mating occurred) and the water levels and hydroperiods we observed, the overall potential for reproductive success for *P*. *crucifer* was high at this site during 2008 and 2011, whereas early wetland drying ensured no reproductive success for *P*. *crucifer* (or any other amphibian species) during 2009 (Figs 22, 24, and 23, respectively). In contrast, low water levels (the depth logger was at the deepest location we could find in each wetland) during late April and early May of 2010 (Fig 18) likely reduced survival of embryos or larvae that resulted from any mating that occurred during the initial wave of calling activity. However, water levels and the hydroperiod after rainfall rehydrated the wetland in early May likely were sufficient to allow embryos or larvae present then or afterwards to mature to metamorphosis (Fig 18), without considering other factors. The dynamics in 2012 were similar to those in 2010, except that the lowest water level occurred earlier in 2012 and likely affected fewer, if any, embryos or larvae than in 2010 because calling activity had occurred over fewer days to that point in 2012 and any actual mating almost certainly was more limited (Fig 21). Thus, our detailed data indicate that the variable intra-seasonal precipitation and surface-water availability we observed across years at SC4DAI2 were sufficient to allow *P*. *crucifer* to reproduce successfully to varying degrees during four of the five years of this study. Yet, these data also demonstrate how amphibians and other wetland-dependent species at such sites are vulnerable to shifts in precipitation frequencies, quantities, or timing due to climate or other factors, as has been reported previously (e.g., [13], [104], [107]).

Water levels and hydroperiods in several of our study wetlands in the SC and in the NTL were dynamic in ways similar to those we observed for SC4DAI2, whereas they fluctuated less at other, typically deeper, sites (Figs 15 and 16). This suggests the relative vulnerabilities of these wetlands and their breeding subpopulations of amphibians to any future droughts, similar to disparities among sites others have described [113], [114]. In contrast, our study wetlands in the Tam contained surface water more consistently (Fig 14), despite receiving less precipitation in general (Fig 8). This appeared to be due to Tam wetlands being deeper and to suspected inputs from ground water (e.g., [115]), making wetlands and associated breeding amphibian subpopulations in this area potentially less vulnerable to low precipitation within and across seasons. Thus, based simply upon the availability of wetland surface water, reproductive success for *P*. *crucifer* and other amphibian species likely was consistently higher in Tam than in our other study areas during this study. Some models predict drier conditions in the future for the area around Tam [83], which could change this ranking.

The relatively high-value information we obtained from our daily measurements of precipitation, water levels, and amphibian calling activity via automated weather stations, data loggers, and acoustic recorders suggests that a daily scale could be important to consider for assessing impacts of climate change on wetlands and wetland communities, as others have submitted [107], [116]. Our data also included information on the occurrence of extreme or intense precipitation events (≥ 5 cm of rainfall in 24 h [83]) that further reinforces the potential importance of a daily time scale. Extreme precipitation events were relatively uncommon during this study. However, they played an important role along with other substantial rainfall events in increasing diminishing water levels and extending hydroperiods (e.g., Figs 18 and 21–24), to the potential benefit of amphibians and other organisms dependent upon these wetlands. The influence of extreme precipitation events on ecological processes and conditions will be important to understand moving forward, given the frequency of such events is projected to increase in this region because warmer air will contain more water [83].

### 4.3. Site occupancy *P*. *crucifer*

We did not measure relative abundance for *P*. *crucifer* during this study. However, the consistently high site occupancy (Table 2) and the substantial calling activity we observed for *P*. *crucifer* across most sites in each study area were informative. They indicate that the temperature and precipitation dynamics, wetland surface-water availability, and landscape productivity and water conditions we observed from 2008 to 2012, as well as those that occurred immediately prior to 2008, were suitable for individuals in these populations to reproduce successfully and for substantial recruitment to have occurred. Given this, and *P*. *crucifer’s* aforementioned aptness for indicating suitable conditions for other amphibian species that dwelled in our study wetlands, our data do not suggest that recent climate conditions, or other global-change factors such as nearby land use, had affected the persistence of amphibian populations in our study areas. This is similar to results from research in these and other study areas in this region [117], but in contrast to pervasive declines reported recently for amphibian populations throughout the United States [11], [105], including on landscapes managed similarly for conservation.

### 4.4. Seasonal ET and NDVI in relation to temperature and precipitation

Results from several studies have described seasonal changes in ET and NDVI that were coincidental with changes in temperature and precipitation, but mostly for arid and semiarid landscapes and for cropland (e.g., [58], [76], [92], [118–121]). Outcomes from studies conducted in humid or sub-humid regions have shown fewer clear relations of ET and NDVI to weather dynamics (e.g., [122–125]. Results from these latter studies likely were related to combinations of factors, such as technical limitations imposed by satellite data that were descriptive only at relatively coarse spatial and/or temporal scales and the functional traits of plant species. For example, the presence of plants with root systems that access water deeper in the soil could mask the presence of less-resistant, water-stressed species on the same landscape at the scale of coarse pixels (or local landscape blocks in our case) [126]. Given these factors, we did not know beforehand if we would be able to detect any inter-annual associations of ET and NDVI with temperature and precipitation in our study areas, which were dominated by relatively humid temperate forests.

We learned that these ET and NDVI metrics did reflect inter-annual differences in temperature and precipitation dynamics that occurred from 2008 to 2012, but we needed to assess temperature and precipitation dynamics together to interpret these metrics meaningfully. For example, both ET and NDVI values changed relative to seasonal temperatures when precipitation was not limiting for transpiration and photosynthesis, but were depressed during drier periods, such as in 2009. Although both ET and NDVI were strongly associated with GDU at all three time intervals we analyzed, the strength of the relation increased with the length of the time step (cumulative to-date > four-week > weekly). This pattern was even more pronounced with the relation of ET and NDVI values to precipitation, as associations with cumulative-to-date precipitation were approximately twice as strong as associations with weekly precipitation (Table 3). Perhaps it is not surprising that cumulative measures of metabolic and physiological activity for the mostly woody plants in our landscape blocks reflected water availability more strongly than shorter-term measures, given the potential for high daily variance in precipitation, the complex dynamics of water movement and retention on the landscape, and, importantly, that plants can access ground water that resulted from past precipitation events. Thus, daily or weekly levels of transpiration and photosynthetic activity for trees and other woody plants in particular might not reflect short-term fluctuations in precipitation under non-extreme conditions. In contrast, fluctuations we observed in the calling activity of *P*. *crucifer* over the course of a few days at individual study wetlands often did indicate coincidental fluctuations in temperature, precipitation, and wetland surface-water levels (e.g., Figs 18 and 21–24), further illustrating differences in the information we obtained at the broader landscape versus the individual wetland scale [82].

Overall, our seasonal ET and NDVI values were aligned more often with GDU (thus, temperature) than with precipitation, but our results suggest the important interplay of temperature and precipitation as drivers of plant activity across the landscape, similar to what we observed for wetland water levels and amphibian calling activity/potential reproductive success in individual wetlands. Our ET and NDVI findings are consistent with results from Vicente-Serrano et al.’s [125] much coarser-scaled global analysis, which showed annual vegetation growth was more sensitive to temperatures in regions where water was not limiting, whereas it was more sensitive to precipitation in semiarid and arid regions.

### 4.5. The value of long-term field research as a complement to modeling climate-related ecological changes

Our satellite-based ET, NDVI, and snow data complemented the data we collected via ground-based sensors by providing coincidental, ecological information at a landscape scale that we could not have attained from the ground, yet a scale that clearly was important for the survival and persistence of amphibians and many other species that dwell on these wetland-upland landscapes. Integrating these broader-scale data with the finer-scaled data we collected at individual wetlands enabled us to describe the apparent primacy, as well as the nuance, of variable and interacting temperature and precipitation in driving environmental conditions and biological productivity across the landscapes we studied. This integrated information also illustrates how and why further changes in climate dynamics outside of recent historical norms could threaten productivity and biodiversity on these landscapes.

Importantly, our results also provide previously non-existent, much-needed scientific information regarding recent relations of temperature and precipitation to key ecological processes and conditions within each of our study areas. Continuing these efforts will allow us to report changes in the baseline conditions we have described as a part of ongoing due diligence to document actual climate-related changes in these areas. Our data also are highly relevant raw materials for creating and updating complementary models of future changes. Such a combination of near real-time understanding of ecological conditions and forecasts of future changes based upon pertinent data can help facilitate effective adaptive management [26].

Modeling future climate-related ecological changes in this region and elsewhere without robust field data could result in highly uncertain predictions. The reasons for this generally are twofold. First, sufficiently accurate predictions of even near-term weather, let alone future climate, at ecologically relevant spatial and temporal scales [83] can be difficult for the north-central United States, despite the availability of extensive climate data, because several, multidirectional, atmospheric forces can influence the weather in this region [19]. Added to this are universal uncertainties associated with modeling changes in climate for predicting ecological effects, such as non-stationarity and the unpredictability of the timing and extent of extreme weather events or ecological state changes [20], [21], [23], [24], [127]. Given that sufficiently accurate climate predictions are foundational to effective ecological forecasting, all such uncertainty is problematic.

Secondly, correctly predicting climate-related ecological effects also is hindered by a paucity of appropriate long-term data sets, i.e., data sets that contain measures of key ecological variables collected *in situ*, at relevant locations and scales, and over a range of weather/climate conditions. Without such data, modelers are left with modeling extant data sets likely produced for other areas or purposes unrelated to the questions at hand, which can result in errant predictions. For example, modelers might presume critical details of how such data were produced and about their reliability/quality that are not correct, possibly leading to violations of model or data assumptions [101], [128]. Interpreting such data correctly also can be difficult due to unknown or undescribed technical bounds or other methods constraints in effect when the data were collected, or to underappreciated scale mismatches that are essential to addressing ecological questions appropriately [129–134]. These potential liabilities overlay those due to typical model simplifications or specific disregard of the complex biology and ecological interactions of species when predicting vulnerabilities or effects [130], [131]. Sound and relevant long-term field studies are integral to reducing such data limitations, suggesting that relying solely on modeling without results from such research could be counterproductive to effectively assessing and managing for climate-related ecological risks and future changes.

## Supporting information

S1 Appendix (DOC)

S2 Appendix (DOC)

S3 Appendix (DOC)

S4 Appendix (DOC)

S5 Appendix (DOC)

S6 Appendix (DOC)

S7 Appendix (DOC)

S8 Appendix (DOC)

S9 Appendix (DOC)

S10 Appendix (DOC)

S1 Table The weather stations from which we obtained data to compare with data we collected via ground-based sensors at our individual study wetlands or to establish historical climate context for our four study areas. “Primary” in the first column identifies the weather station from which we obtained the majority of weather data for the associated specific study wetlands. “Secondary” in the first column refers to an alternative local weather station from which we obtained additional data, when necessary and appropriate, to replace missing or questionable data from primary stations. We did not use a secondary station when data sets from primary stations were sufficient. Tam = Tamarac National Wildlife Refuge. NWS = National Weather Service. ID = Identifier. RAWS = Remote automated weather station. T = air temperature. P = precipitation. NOAA = National Oceanic and Atmospheric Administration. GHCND = Global Historical Climatology Network Daily. COOP = National Weather Service Cooperative Observer Station; WS = Weather station. AWOS = Automated Weather Observing System. MN = Minnesota. SC = St. Croix National Scenic Riverway. WI = Wisconsin. NTL = North Temperate Lakes Long-term Ecological Research site. UMR = Upper Mississippi River. WS = Weather Underground station. R/WIS = Road and Weather Information System; KONA = the Municipal Airport in Winona, MN.
(DOCX)

S2 Table Missing or questionable data we identified in weather-station data sets and the substitutions we made for such data. The information in this table applies specifically to weather-station data (S1 Table) we used for comparing with data collected via ground-based sensors at individual study wetlands. We did not list any weather stations for which we did not identify missing or questionable data. ID = Identifier. Tam = Tamarac National Wildlife Refuge. MN = Minnesota. RAWS = Remote automated weather station. NWS = National Weather Service. P = Precipitation. GHCND = Global Historical Climatology Network Daily. SC = St. Croix National Scenic Riverway. WI = Wisconsin. T = air temperature. Tmean = mean daily air temperature. NTL = North Temperate Lakes Long-term Ecological Research site. UMR = Upper Mississippi River. COOP = National Weather Service Cooperative Observer Station. KONA = the Municipal Airport at Winona, MN.
(DOCX)

S3 Table Sources of recent and historical weather data we used for remote-sensing analyses. “Primary” in the first column identifies the weather station from which we obtained the majority of weather data for the associated specific study wetlands. “Secondary” in the first column refers to an alternative local weather station from which we obtained additional data, when necessary and appropriate, to replace missing or questionable data from primary stations. We did not use a secondary station when data sets from primary stations were sufficient. Tam = Tamarac National Wildlife Refuge. NWS = National Weather Service. ID = Identifier. RAWS = Remote automated weather station. MN = Minnesota. T = temperature. P = Precipitation. WS = Weather Underground station. KDTL = Detroit Lakes Airport-Wething Field. AWOS = Automated Weather Observing System; SC = St. Croix National Scenic Riverway. NOAA = National Oceanic and Atmospheric Administration. GHCND = Global Historical Climatology Network Daily. WI = Wisconsin. KRZN = Burnett County Airport. KHYR = Sawyer County Airport. ASOS = Automated Surface Observing System. NTL = North Temperate Lakes Long-term Ecological Research site. KARV = Lakeland Airport/Noble F. Lee Memorial Field. UMR = Upper Mississippi River.
(DOCX)

S4 Table Missing or questionable data we identified in weather-station data sets and the substitutions we made for such data. The information in this table applies specifically to weather-station data (S3 Table) we used for comparing with data collected via satellite sensors for individual landscape blocks in each study area. We did not list any weather stations for which we did not identify missing or questionable data. ID = Identifier. Tam = Tamarac National Wildlife Refuge. MN = Minnesota. RAWS = Remote automated weather station. NWS = National Weather Service. Tmin = daily minimum air temperature. Tmax = daily maximum air temperature. P = Precipitation. KDTL = Detroit Lakes Airport-Wething Field. SC = St. Croix National Scenic Riverway. WI = Wisconsin. GHCND = Global Historical Climatology Network Daily. KRZN = Burnett County Airport. KHYR = Hayward Municipal Airport. NTL = North Temperate Lakes Long-term Ecological Research site. UMR = Upper Mississippi River. KARV = Lakeland/Noble F. Lee Memorial Field Airport.
(DOCX)

S5 Table Crosswalk between week number versus days-of-year for seven-day and eight-day intervals for January–August. Source satellite data for the normalized vegetation index product were provided as seven-day composites on a rolling schedule across years. Source satellite data for snow and evapotranspiration products were provided as eight-day products beginning on January 1 each year. Air temperature and precipitation were daily weather-station data that we summarized at eight-day intervals for field-based analyses and seven-day intervals for remote sensing-based analyses. “Start of Month” is provided for context.
(DOCX)

S1 Fig Comparison of original and temporally smoothed normalized difference vegetation index (NDVI) data. (TIF)

S2 Fig Weekly normalized difference vegetation index (NDVI) for site SC12DAI1 in the St. Croix National Scenic Riverway. Automated algorithms for detecting the start-of-season for primary productivity can be influenced by apparent false green-up (increases in NDVI values during the first eight weeks of the year in this example) that results from snowmelt exposing underlying evergreen vegetation.
(TIF)

S3 Fig The daily total precipitation for days when precipitation was recorded at the remote automated weather station at Lind, Wisconsin, from 2008 to 2012. Data for each year cover the period when we deployed a pressure logger to measure surface-water depth at site SC4DAI2 in the St. Croix National Scenic Riverway. The total number of days we deployed a logger at SC4DAI2 and all sites varied across years due mostly to the timing of snowmelt and logistical constraints. These annual data profiles are similar to those we observed for other weather stations and study areas. The data are spread out around the grid lines on the x axis so that all data points for each year are at least partially visible.
(TIF)

S4 Fig The change in daily surface-water depth, for days when such changes were positive, at site SC4DAI2 in the St. Croix National Scenic Riverway from 2008 to 2012. The total number of days we deployed a pressure logger at SC4DAI2 and all sites varied across years due mostly to the timing of snowmelt and logistical constraints. These annual data profiles are similar to those we observed for other study sites across study areas. The data are spread out around the grid lines on the x axis so that all data points for each year are at least partially visible.
(TIF)

S5 Fig The relation of the number of days precipitation was recorded for each of our study sites (where we deployed pressure loggers to measure depth) in each study area to the number of days water depth increased at those sites from 2008 to 2012. Tam, SC, NTL, and UMR = the Tamarac National Wildlife Refuge, St. Croix National Scenic Riverway, North Temperate Lakes Long-term Ecological Research site, and the Upper Mississippi River study areas, respectively.
(TIF)

S6 Fig The same data as in S5 Fig with individual lines fitted only for the data specific to each study area. (TIF)

S7 Fig Boxplots, including the individual data points (filled circles), for the number of days we measured increases in water depth at each site with a pressure logger in each study area from 2008 to 2012. Tam, SC, NTL, and UMR = the Tamarac National Wildlife Refuge, the St. Croix National Scenic Riverway, and the Upper Mississippi River, respectively. The area within each box represents the span of the middle 50% of the data (25 to 75%), the line within each box represents the median value, and the whiskers extend out to the largest and smallest values for each box.
(TIF)

S8 Fig Climatograph for all sites in the Tamarac National Wildlife Refuge. (TIF)

S9 Fig Climatograph for the St. Croix National Scenic Riverway, site SC1DA3. (TIF)

S10 Fig Climatograph for the St. Croix National Scenic Riverway, sites SC4DA3, SC4DAI2, SC4DB9, and SC4DBI2. (TIF)

S11 Fig Climatograph for the St.Croix National Scenic Riverway, site SC8DAI1. (TIF)

S12 Fig Climatograph for the St. Croix National Scenic Riverway, sites SC10DB1 and SC10DD1. (TIF)

S13 Fig Climatograph for the St. Croix National Scenic Riverway, sites SC12DA4 and SC12DAI1. (TIF)

S14 Fig Climatograph for the North Temperate Lakes Long-term Ecological Research area, sites TRL1DA1, TRL1DB1, TRL2DA1, TRL2DB1, TRL3DA1, TRL3DB1, and TRL3DC1. (TIF)

S15 Fig Climatograph for the North Temperate Lakes Long-term Ecological Research area, sites TRL4DA1, TRL4DB1, and TRL4DC1. (TIF)

S16 Fig Climatograph for the Upper Mississippi River site, TrNWRDA1UMRP4. (TIF)

S17 Fig Climatograph for the Upper Mississippi River site, UMRP4. (TIF)

S18 Fig Climatograph for the Upper Mississippi River sites, PSP1 and UMRRP7. (TIF)

S19 Fig Climatograph for the Upper Mississippi River site, UMRRP10. (TIF)
