# Supplementary material for: Multi-year data from satellite- and ground-based sensors show details and scale matter in assessing climate’s effects on wetland surface water, amphibians, and landscape conditions
Source: PLoS One. 2018 Sep 7;13(9):e0201951. doi: 10.1371/journal.pone.0201951 (PMC6128473; doi:10.1371/journal.pone.0201951)
Supplement: S1 Appendix — (DOC) [file pone.0201951.s001.doc]

To produce the sun shield, we cut a 25.4-cm long section of 10.2-cm diameter plastic pipe in half longitudinally and used one of the halves. We drilled holes in the shield to allow air circulation and wired it, concave side down, to a tree adjacent to a study wetland at a point approximately two meters above the ground, such that the long axis of the shield was parallel with the ground along an east-west axis.
