# Supplementary material for: Multi-year data from satellite- and ground-based sensors show details and scale matter in assessing climate’s effects on wetland surface water, amphibians, and landscape conditions
Source: PLoS One. 2018 Sep 7;13(9):e0201951. doi: 10.1371/journal.pone.0201951 (PMC6128473; doi:10.1371/journal.pone.0201951)
Supplement: S2 Appendix — (DOC) [file pone.0201951.s002.doc]

Because Wildlife Acoustics produced recorder models sequentially across years and we purchased the model available when we initiated research in a specific study area or replaced units, we used SM1s exclusively in the SC, NTL, at two sites in the UMR, and throughout Tam in 2009 only. We used SM2s exclusively in the Tam after 2009 and at three sites in the UMR throughout. These models differed primarily in the placement of microphones, power consumption, data-storage capacity, and programmability. We did not observe any differences in terms of the quality of the recordings.
