# Supplementary material for: Multi-year data from satellite- and ground-based sensors show details and scale matter in assessing climate’s effects on wetland surface water, amphibians, and landscape conditions
Source: PLoS One. 2018 Sep 7;13(9):e0201951. doi: 10.1371/journal.pone.0201951 (PMC6128473; doi:10.1371/journal.pone.0201951)
Supplement: S3 Appendix — (DOC) [file pone.0201951.s003.doc]

We initially recorded in the .wav format, but switched to the compressed .wac format when Wildlife Acoustics made it available in 2009 to reduce requirements for storage space on data cards. We adjusted microphone gains at the start of each season when necessary based upon the manufacturer’s recommendations. We replaced batteries and data cards during the field season on regular rotations before power in the batteries (the most limiting factor) was depleted. Typically, this schedule resulted in visiting our study wetlands every 40 to 50 days to refurbish recorder models SM1 and SM2, respectively. This conservative schedule reduced the chances recorders would fail due to depleted batteries.
