# Supplementary material for: Multi-year data from satellite- and ground-based sensors show details and scale matter in assessing climate’s effects on wetland surface water, amphibians, and landscape conditions
Source: PLoS One. 2018 Sep 7;13(9):e0201951. doi: 10.1371/journal.pone.0201951 (PMC6128473; doi:10.1371/journal.pone.0201951)
Supplement: S4 Appendix — (DOC) [file pone.0201951.s004.doc]

This model estimated actual ET with an energy-balance approach that used the remotely sensed land-surface temperature to solve for the latent heat-energy component as a product of ET fraction (0−1.0) generated from land-surface temperature and atmospheric demand (potential ET) estimated using the Penman-Monteith equation. Main model inputs were eight-day average land-surface temperatures from the Collection-5 MODIS MOD11A product [1], air temperatures in the form of gridded surfaces interpolated and extrapolated from daily meteorological observations by the Daily Surface Weather and Climatological Summaries (“DAYMET”) model [2], [3], and potential ET [4] derived from weather fields produced by the Global Data Assimilation System [5].

**References**

1. Wan Z. Collection-5 MODIS land surface temperature products user’s guide. 2007. Available from: http://www.icess.ucsb.edu/modis/LstUsrGuide/MODIS_LST_products_Users_guide_C5.pdf. Accessed 13 February 2017.

2. Thornton PE, Running SW, White MA. Generating surfaces of daily meteorology variables over large regions of complex terrain. J Hydrol 1997; 190: 214−251.

3. Thornton PE, Thornton MM, Mayer BW, Wilhelmi N, Wei Y, Cook RB. Daymet: Daily surface weather on a 1 km grid for North America, 1980−2008. 2012. Available from: http://daymet.ornl.gov. Accessed 13 February 2017.

4. Senay GB, Verdin JP, Lietzow R, Melesse AM. Global reference evapotranspiration modeling and evaluation. J Am Water Resour Assoc 2008; 44(4): 969−979.

5. Kanamitsu M. Description of the NMC Global Data Assimilation and Forecast System. Weath Forecas 1989; 4: 334−342.
