# Supplementary material for: Multi-year data from satellite- and ground-based sensors show details and scale matter in assessing climate’s effects on wetland surface water, amphibians, and landscape conditions
Source: PLoS One. 2018 Sep 7;13(9):e0201951. doi: 10.1371/journal.pone.0201951 (PMC6128473; doi:10.1371/journal.pone.0201951)
Supplement: S5 Appendix — (DOC) [file pone.0201951.s005.doc]

This approach employed a moving window operating on the time-series data to calculate a regression line. The window moved one period at a time, resulting in a family of regression lines associated with each data point. This family of lines then was averaged at each point and interpolated between points to provide a continuous, smoothed signal over time. Because noisy environmental and instrumental phenomena usually reduce NDVI values, the smoothing process used a weighting factor that favored (and retained) peak points over sloping or valley points (S1 Fig). The resulting relations between raw and smoothed data were statistically based [1, 2] and provided a closer approximation of greenness than the original, noisy NDVI data series.

**References**

1. Swets, DL, Reed BC, Rowland JD, Marko SE. A weighted least-squares approach to temporal NDVI smoothing. In: Proceedings of the 1999 ASPRS annual conference. Portland, Oregon. 17−21 May 1999.

2. Brown JF. Temporally smoothed weekly AQUA Collect 6 Moderate Resolution Imaging Spectroradiometer (MODIS) Normalized Difference Vegetation Index (NDVI) at 250 meter: U.S. Geological Survey; 2018 [cited 2018 Jun 7]. Available from: https://phenology.cr.usgs.gov/get_data_smNDVI.php Unique ID: doi:10.5066/F7BR8RGQ.
