# Supplementary material for: Multi-year data from satellite- and ground-based sensors show details and scale matter in assessing climate’s effects on wetland surface water, amphibians, and landscape conditions
Source: PLoS One. 2018 Sep 7;13(9):e0201951. doi: 10.1371/journal.pone.0201951 (PMC6128473; doi:10.1371/journal.pone.0201951)
Supplement: S6 Appendix — (DOC) [file pone.0201951.s006.doc]

Whenever daily data values for temperature or precipitation appeared to be outside of likely potential ranges, we compared those data with observations from other weather stations in the area and with the temperature (2012 only) and precipitation values we measured in each of our study areas for this purpose. We did not use data for individual dates when we concluded data were errant. Instead we substituted data from another nearby weather station or an individual site, as appropriate, or excluded individual dates from our analyses when substitutions were not appropriate. We adjusted for any missing data in the same manner. We did not need to make changes very often (S1and S2 Tables). Importantly, substituting temperature and precipitation (for dates when precipitation actually occurred) data from non-automated stations could increase uncertainty regarding daily conditions because human observers at such stations typically recorded temperature values at the beginning of each work day based upon maximum and minimum temperatures recorded over the previous 24 hours and precipitation accumulated in rain gauges over the same period. They then ascribed those temperature and precipitation values to the date of the previous day. Thus, one could not be certain if the minimum temperatures or the precipitation totals reported for a date occurred on the date to which they were ascribed or during the hours from midnight to the time when the observer checked the gauges and recorded the data the next day. This was not an issue when precipitation did not occur during a given date, as confirmed via data from other nearby weather stations or rain gauges deployed in study areas.
