# Supplementary material for: Multi-year data from satellite- and ground-based sensors show details and scale matter in assessing climate’s effects on wetland surface water, amphibians, and landscape conditions
Source: PLoS One. 2018 Sep 7;13(9):e0201951. doi: 10.1371/journal.pone.0201951 (PMC6128473; doi:10.1371/journal.pone.0201951)
Supplement: S7 Appendix — (DOC) [file pone.0201951.s007.doc]

Ensuring that daily temperature and precipitation data pertained specifically to the date for which they were recorded was less critical for our analyses using remotely sensed data because they applied to coarser temporal scales (seven-day and eight-day products). Thus, we acquired data for relating NDVI and ET dynamics to temperature and precipitation from non-automated weather stations when they were closest to our field sites (S3 and S4 Tables). Otherwise, we followed the same procedures for assessing the quality of weather-station data as described in § 2.4.2. and S6 Appendix.
