# Supplementary material for: Multi-year data from satellite- and ground-based sensors show details and scale matter in assessing climate’s effects on wetland surface water, amphibians, and landscape conditions
Source: PLoS One. 2018 Sep 7;13(9):e0201951. doi: 10.1371/journal.pone.0201951 (PMC6128473; doi:10.1371/journal.pone.0201951)
Supplement: S8 Appendix — (DOC) [file pone.0201951.s008.doc]

Masking occurred when louder sounds (evidenced by the color scheme on the contour plot) from biotic and abiotic sources (birds, storms, and sound distortion, e.g.) occurred in the same bandwidths as calls for targeted amphibian species, effectively hiding any amphibian calls that might have occurred on the contour plot. Potential masking occurred on practically all contour plots, but it varied by the year, site, and bandwidth within which a targeted amphibian call occurred. Calls were masked especially when they were few in number, very low in dB levels, or occurred in low-frequency bandwidths, and most often for *Lithobates sylvaticus*.
