# Supplementary material for: Multi-year data from satellite- and ground-based sensors show details and scale matter in assessing climate’s effects on wetland surface water, amphibians, and landscape conditions
Source: PLoS One. 2018 Sep 7;13(9):e0201951. doi: 10.1371/journal.pone.0201951 (PMC6128473; doi:10.1371/journal.pone.0201951)
Supplement: S9 Appendix — (DOC) [file pone.0201951.s009.doc]

Using our integrated approach, we first examined the contour plot for each individual study wetland to see when call signatures of early calling species, such as *Pseudacris crucifer*, *P. maculata*, and *Lithobates sylvaticus*, were unequivocally visible initially on the plot. From that date, we then carefully assessed sound patterns for each preceding date and hour, especially but not exclusively from 0000 to 0400 h and 1200 to 2300 h (likely amphibian calling periods during late winter or early spring in our study areas), on the contour plot to determine when any call signatures might be visible, but were not unequivocal, or when louder sounds could have masked individual or quiet calls*.* For each acoustic sample (the original five-minute recording from a unique date-hour combination) where such uncertainty occurred on the contour plot, we assessed the original recording aurally and visually to determine if calls occurred. Except in rare extreme cases when exceptionally loud noises from clipping or heavy thunderstorms occurred, amphibian calls typically were visible and/or audible within the individual recording regardless of whether they were visible on the contour plot.
