# Supplementary material for: Multi-year data from satellite- and ground-based sensors show details and scale matter in assessing climate’s effects on wetland surface water, amphibians, and landscape conditions
Source: PLoS One. 2018 Sep 7;13(9):e0201951. doi: 10.1371/journal.pone.0201951 (PMC6128473; doi:10.1371/journal.pone.0201951)
Supplement: S10 Appendix — (DOC) [file pone.0201951.s010.doc]

The dB levels from 2900 to 3200 Hz could have been enhanced due to calls of *Pseudacris maculata* (Fig 2) when they called simultaneously with *P. crucifer*. The effects of any such enhancements on our assessments were limited by the substantially greater sound intensity in the calls of *P. crucifer* and the narrow extent of overlap between calls of the two species over 2900 to 3200 Hz (Fig 2), as well as our observations that the two species generally called during the same evening hours at our study sites. Given all this, and that separating all overlapping sound from the two species was impossible when they called simultaneously, we did not discard dates where such overlap occurred, but we did confirm that *P. crucifer* had called on all dates we ultimately described as calling dates for this species.

We dealt with other overlapping sounds differently. Sounds from rain, thunder, birds, and other sources contributed substantially to sound intensity on some dates. To address the potential problems of these sounds resulting in misleading dB levels when *P. crucifer* called or suggesting *P. crucifer* called when they did not, we removed dates from further analyses that we determined had overlapping sounds with unacceptable dB levels. To determine these dates, we screened each day throughout *P. crucifer’s* calling period by first examining the contour plot and scanning recordings in Songscope (Wildlife Acoustics; Maynard, MA) to identify when overlapping sounds occurred during the recordings at 2100, 2200, or 2300 hours and whether *P. crucifer* had called as well. If such calling had not occurred and the intensity of the overlapping sounds was > -72 dB, a level we chose as a conservative, low-intensity threshold, we removed those dates from further analyses. If *P. crucifer* had called, we removed any dates during which the dB levels of overlapping sounds were < 40 dB different than the levels of the *P. crucifer* calls. This equated to the sound intensity of the overlapping sounds being 1% or less of the sound intensity of coincidental *P. crucifer* calls. We considered this separation also to be a conservative threshold for removing dates from further analyses.
