# Supplementary material for: Multi-year data from satellite- and ground-based sensors show details and scale matter in assessing climate’s effects on wetland surface water, amphibians, and landscape conditions
Source: PLoS One. 2018 Sep 7;13(9):e0201951. doi: 10.1371/journal.pone.0201951 (PMC6128473; doi:10.1371/journal.pone.0201951)
Supplement: S1 Table — “Primary” in the first column identifies the weather station from which we obtained the majority of weather data for the associated specific study wetlands. “Secondary” in the first column refers to an alternative local weather station from which we obtained additional data, when necessary and appropriate, to replace missing or questionable data from primary stations. We did not use a secondary station when data sets from primary stations were sufficient. Tam = Tamarac National Wildlife Refuge. NWS = National Weather Service. ID = Identifier. RAWS = Remote automated weather station. T = air temperature. P = precipitation. NOAA = National Oceanic and Atmospheric Administration. GHCND = Global Historical Climatology Network Daily. COOP = National Weather Service Cooperative Observer Station; WS = Weather station. AWOS = Automated Weather Observing System. MN = Minnesota. SC = St. Croix National Scenic Riverway. WI = Wisconsin. NTL = North Temperate Lakes Long-term Ecological Research site. UMR = Upper Mississippi River. WS = Weather Underground station. R/WIS = Road and Weather Information System; KONA = the Municipal Airport in Winona, MN. (DOCX) [file pone.0201951.s011.docx]

| Study Area | Study Wetlands | Weather Station; Type; Location | Latitude  Longitude^1^ | Data Used | Relevant Figures and Tables |
| --- | --- | --- | --- | --- | --- |
| Tam  (primary) | All Tam sites | NWS ID 212201; RAWS^2^; Detroit Lakes, MN | 46.848889  -95.846389 | T and P | Figures 5, 6, 8, 9a  Table 1 |
| Tam  (secondary) | All Tam sites | NOAA^3^ ID USC00212142; GHCND; Detroit Lakes, MN | 46.83722  95.83722 | We used P data from this station to replace missing P data from the Tam primary station. | Figure 8  Table 1 |
| SC | SC1DA3 | NWS ID 214201; RAWS; Carlos Avery, MN | 45.302778  -93.101111 | T and P | Figures 9a  Table 1 |
| SC  (primary) | SC4DB9  SC4DBI2  SC4DA3  SC4DAI2 | NWS ID 470602; RAWS; Lind, WI | 45.739722  -92.795556 | T and P | Figures 5, 6, 8, 9a, 17, 20–23  Table 1 |
| SC  (secondary) | SC4DB9  SC4DBI2  SC4DA3  SC4DAI2 | NOAA ID USW00014995; GHCND; Grantsburg, WI | 45.772423  -92.689327 | We used T data from this station to replace missing T data from the SC primary station at Lind, WI. | Figures 5, 6, 9a, 17, 20–23  Table 1 |
| SC | SC8DAI1  SC10DB1  SC10DD1 | NWS ID 470703; RAWS; Minong, WI | 46.135833  -91.980833 | T and P | Figure 9a  Table 1 |
| SC  (primary) | SC12DA4  SC12DAI1 | NWS ID 470304; RAWS; Clam Lake, WI | 46.197778  -90.970278 | T and P | Figure 9a  Table 1 |
| SC  (secondary) | SC12DA4  SC12DAI1 | NWS ID 470804; RAWS; Hayward, WI | 46.031111  -91.449000 | We used T and P data from this station to replace missing T and P data from the SC primary station at Clam Lake, WI. | Figure 9a  Table 1 |
| NTL  (primary) | All NTL sites | NWS ID 471002; RAWS; Woodruff, WI | 45.889722  -89.652222 | T and P | Figures 5, 6, 8, 9a  Table 1 |
| NTL  (secondary) | All NTL sites | NOAA ID USC00475516; GHCND; Minocqua, WI | 45.8863  -89.7322 | We used P data from this station to replace missing P data from the NTL primary station. | Figures 5, 6, 8, 9a  Table 1 |
| UMR  (primary) | UMRP4 | NOAA ID USC00470124; GHCND; and NOAA ID = 470124; COOP; Alma, WI | 44.327220  -91.919440 | We used T data from the GHCND station and P data from the COOP station. | Figures 8, 9a  Table 1 |
| UMR  (secondary) | UMRP4 | WS^4^ ID MMN069; R/WIS; Kellogg, MN | 44.285  -91.990 | We used T data from this station to replace missing T data from the UMR primary station at Alma, WI. | Figure 9a |
| UMR  (primary) | UMRP7  PSP1  TrNWRDA1 | NOAA ID USC00478589; GHCND; and NOAA ID = 478589; COOP; Trempealeau, WI | 43.999400  -91.437800 | We used T data from the GHCND station and P data from the COOP station. | Figures 5, 6, 8, 9a  Table 1 |
| UMR  (secondary) | UMRP7  PSP1  TrNWRDA1 | WS ID KONA; AWOS; Winona, MN | 44.075540  -91.706521 | We used T and P data from this station to replace missing T and P data from the UMR primary station at Trempealeau, WI. | Figures 5, 6, 8, 9a  Table 1 |
| UMR  (secondary) | UMRP7  PSP1  TrNWRDA1 | NOAA ID 470124; COOP; Alma, WI | 44.327220  -91.919440 | We used P data from this station to replace missing P data from the UMR primary station at Trempealeau, WI, and, when necessary, from the UMR secondary station at Winona, MN. | Figures 8, 9a  Table 1 |
| UMR | UMRP10 | NOAA ID USC00476827; GHCND; Prairie du Chien, WI | 43.051500  -91.134900 | T | Figure 9a |
| Tam, SC, NTL, UMR | All study wetlands per each study area | Historical climate data summaries^5^ for Climate Divisions MN1, WI1, WI2, and WI 4, respectively | Multiple by climate division and year^5^. | Historical T and P | Figures 4, 7, 19 |

^1^ North American Datum 1983.

^2^ Data for these stations were from http://www.raws.dri.edu/index.html.

^3^ Data for these stations were from http://www.ncdc.noaa.gov/cdo-web/datasets.

^4^ Data for these stations were from https://www.wunderground.com/.

^5^ Data for these NOAA Climate Divisions were from http://www.esrl.noaa.gov/psd/cgi-bin/data/timeseries/timeseries1.pl.
