# Supplementary material for: Multi-year data from satellite- and ground-based sensors show details and scale matter in assessing climate’s effects on wetland surface water, amphibians, and landscape conditions
Source: PLoS One. 2018 Sep 7;13(9):e0201951. doi: 10.1371/journal.pone.0201951 (PMC6128473; doi:10.1371/journal.pone.0201951)
Supplement: S2 Table — The information in this table applies specifically to weather-station data (S1 Table) we used for comparing with data collected via ground-based sensors at individual study wetlands. We did not list any weather stations for which we did not identify missing or questionable data. ID = Identifier. Tam = Tamarac National Wildlife Refuge. MN = Minnesota. RAWS = Remote automated weather station. NWS = National Weather Service. P = Precipitation. GHCND = Global Historical Climatology Network Daily. SC = St. Croix National Scenic Riverway. WI = Wisconsin. T = air temperature. Tmean = mean daily air temperature. NTL = North Temperate Lakes Long-term Ecological Research site. UMR = Upper Mississippi River. COOP = National Weather Service Cooperative Observer Station. KONA = the Municipal Airport at Winona, MN. (DOCX) [file pone.0201951.s012.docx]

| Study Area | Study Wetlands | Weather Station; Type; Location | Missing Data^1^ | Questionable Data^1^ | Data Substituted for Missing or Questionable Data |
| --- | --- | --- | --- | --- | --- |
| Tam | All TAM sites | NWS ID 212201; RAWS; Detroit Lakes, MN | P for 8–16 Apr 2009 | None | P from station NOAA ID USC00212142 in Detroit Lakes, MN |
| SC | SC4DB9  SC4DBI2  SC4DA3  SC4DAI2 | NWS ID 470602; RAWS; Lind, WI | T for 9 Apr–-3 May 2008 | None | T from station NOAA ID USW00014995 in Grantsburg, WI |
| SC | SC12DA4  SC12DAI1 | NWS ID 470804; RAWS; Clam Lake, WI | T for 27 Jan–-15 March 2010 and for 11 Jan–-28 March 2011 | P of 36.83 cm for 8 July 2008 | T and P from station NWS ID 470804 in Hayward, WI |
| NTL | All NTL sites | NWS ID 471002; RAWS; Woodruff, WI | P for 12–-14 May 2011. | None | P from station NOAA ID USC00475516 in Minocqua, WI. |
| UMR | UMRP4 | NOAA ID USC00470124; GHCND; Alma, WI | T for 16 Mar 2012 | None | T from station WS ID MMN069 in Kellogg, MN. Because of differences in daily T between the Alma and Kellogg stations, we calculated the average difference in the mean daily T between the two sites for the five days before and the five days after the data gap. Prior to the data substitution, we then adjusted the Tmean value from the Kellogg station for 16 Mar by that average difference. |
| UMR | UMRP7  PSP1  TrNWRDA1 | NOAA ID 478589; COOP; Trempealeau, WI | P for 1–-31 Aug 2010, 1–30 Apr 2011, and 31 Aug–-1 Sept 2011. T for 12 March 2012 | None | P from station WS ID KONA Winona, MN, for 1–-31 Aug 2010 and 1–-30 Apr 2011. P from station NOAA ID 470124 in Alma, WI, for 31 Aug–-1 Sept 2011. T from station WS ID KONA in Winona, MN. |

^1^ We did not list dates with missing or questionable data if we did not use those dates in analyses.
