# Supplementary material for: Multi-year data from satellite- and ground-based sensors show details and scale matter in assessing climate’s effects on wetland surface water, amphibians, and landscape conditions
Source: PLoS One. 2018 Sep 7;13(9):e0201951. doi: 10.1371/journal.pone.0201951 (PMC6128473; doi:10.1371/journal.pone.0201951)
Supplement: S3 Table — “Primary” in the first column identifies the weather station from which we obtained the majority of weather data for the associated specific study wetlands. “Secondary” in the first column refers to an alternative local weather station from which we obtained additional data, when necessary and appropriate, to replace missing or questionable data from primary stations. We did not use a secondary station when data sets from primary stations were sufficient. Tam = Tamarac National Wildlife Refuge. NWS = National Weather Service. ID = Identifier. RAWS = Remote automated weather station. MN = Minnesota. T = temperature. P = Precipitation. WS = Weather Underground station. KDTL = Detroit Lakes Airport-Wething Field. AWOS = Automated Weather Observing System; SC = St. Croix National Scenic Riverway. NOAA = National Oceanic and Atmospheric Administration. GHCND = Global Historical Climatology Network Daily. WI = Wisconsin. KRZN = Burnett County Airport. KHYR = Sawyer County Airport. ASOS = Automated Surface Observing System. NTL = North Temperate Lakes Long-term Ecological Research site. KARV = Lakeland Airport/Noble F. Lee Memorial Field. UMR = Upper Mississippi River. (DOCX) [file pone.0201951.s013.docx]

| Study Area | Study Blocks | Weather Station; Type; Location | Latitude  Longitude^1^ | Data Used | Relevant Figures and Tables |
| --- | --- | --- | --- | --- | --- |
| Tam  (primary) | All Tam sites | NWS ID 212201, RAWS^2^; Detroit Lakes, MN | 46.848889  -95.846389 | T and P | Figures 9b, S3a  Table 3 |
| Tam  (secondary) | All Tam sites | WS^3^ ID KDTL; AWOS; Detroit Lakes, MN | 46.83  -95.89 | We used T and P data from this station to replace missing T and P data from the Detroit Lakes RAWS station. | Figures 9b, S3a  Table 3 |
| SC  (primary) | SC1DA3 | NOAA^4^ ID USC00212881; GHCND; Forest Lake, MN | 45.3397  -92.9125 | T and P | Figures 9b, S3b  Table 3 |
| SC  (primary) | SC4DA3  SC4DAI2  SC4DB9  SC4DBI2 | NWS ID 470602, RAWS; Lind, WI | 45.739722  -92.795556 | T and P | Figures 9b, S3c  Table 3 |
| SC  (primary) | SC8DAI1 | NWS ID 470703, RAWS; Minong, WI | 46.135833  -91.980833 | T and P | Figures 9b, S3d  Table 3 |
| SC  (primary) | SC10DB1  SC10DD1 | NOAA ID USC00478027; GHCND; Spooner, WI | 45.8236  -91.8761 | T and P | Figures 9b, S3e  Table 3 |
| SC  (secondary) | SC10DB1  SC10DD1 | WS ID KRZN; AWOS; Siren, WI | 45.823464  -92.373692 | We used T and P data from this station to replace missing T and P data from the Spooner GHCND station. | Figures 9b, S3e  Table 3 |
| SC  (primary) | SC12DA4  SC12DAI1 | NWS ID 470804; RAWS; Hayward, WI | 46.031111  -91.449000 | T and P | Figures 9b, S3f  Table 3 |
| SC  (secondary) | SC12DA4  SC12DAI1 | WS ID KHYR; ASOS; Hayward, WI | 46.0303  -91.4426 | We used T and P data from this station to replace missing T and P data from the Hayward RAWS station. | Figures 9b, S3f  Table 3 |
| NTL  (primary) | TRL1DA1  TRL1DB1  TRL2DA1  TRL2DB1  TRL3DA1  TRL3DB1  TRL3DC1 | NWS ID 471002, RAWS; Woodruff, WI | 45.889722  -89.652222 | T and P | Figures 9b, S3g  Table 3 |
| NTL  (secondary) | TRL1DA1  TRL1DB1  TRL2DA1  TRL2DB1  TRL3DA1  TRL3DB1  TRL3DC1 | NWS ID KARV; AWOS; Arbor Vitae, WI | 45.9264  -89.7307 | We averaged T and P data from this station and the Minocqua station to replace missing T and P data from the Woodruff RAWS station. | Figures 9b, S3g  Table 3 |
| NTL  (secondary) | TRL1DA1  TRL1DB1  TRL2DA1  TRL2DB1  TRL3DA1  TRL3DB1  TRL3DC1 | NOAA ID USC00475516; GHCND; Minocqua, WI | 45.8863  -89.7322 | We averaged T and P data from this station and the Arbor Vitae station to replace missing T and P data from the Woodruff RAWS station. | Figures 9b, S3g  Table 3 |
| NT  (primary) | TRL4DA1  TRL4DB1  TRL4DC1 | NWS ID 470302, RAWS; Glidden, WI | 46.14000  -90.00000 | T and P | Figures 9b, S3h  Table 3 |
| UMR  (primary) | TrNWRDA1 | NOAA ID USC00472165; GHCND; Dodge, WI | 44.1330  -91.5511 | T and P | Figures 9b, S3i  Table 3 |
| UMR  (primary) | UMRP4 | NOAA ID USC00470124; GHCND; Alma, WI | 44.327220  -91.919440 | T | Figures 9b, S3j  Table 3 |
| UMR  (primary) | PSP1  UMRP7 | NOAA ID USC00478589; GHCND; Trempealeau, WI | 43.9994  -91.4378 | T and P | Figures 9b, S3k  Table 3 |
| UMR  (primary) | UMRP10 | NOAA ID USC00476827; GHCND; Prairie du Chien, WI | 43.051500  -91.134900 | T | Figures 9b, S3l  Table 3 |

^1^ North American Datum 1983.

^2^ Data for these RAWS stations were from http://www.raws.dri.edu/index.html.

^3^ Data for these stations were from https://www.wunderground.com/.

^4^ Data for these NOAA stations were from http://www.ncdc.noaa.gov/cdo-web/datasets.

^5^ Data for these NOAA Climate Divisions were from http://www.esrl.noaa.gov/psd/cgi-bin/data/timeseries/timeseries1.pl.
