# Supplementary material for: Multi-year data from satellite- and ground-based sensors show details and scale matter in assessing climate’s effects on wetland surface water, amphibians, and landscape conditions
Source: PLoS One. 2018 Sep 7;13(9):e0201951. doi: 10.1371/journal.pone.0201951 (PMC6128473; doi:10.1371/journal.pone.0201951)
Supplement: S4 Table — The information in this table applies specifically to weather-station data (S3 Table) we used for comparing with data collected via satellite sensors for individual landscape blocks in each study area. We did not list any weather stations for which we did not identify missing or questionable data. ID = Identifier. Tam = Tamarac National Wildlife Refuge. MN = Minnesota. RAWS = Remote automated weather station. NWS = National Weather Service. Tmin = daily minimum air temperature. Tmax = daily maximum air temperature. P = Precipitation. KDTL = Detroit Lakes Airport-Wething Field. SC = St. Croix National Scenic Riverway. WI = Wisconsin. GHCND = Global Historical Climatology Network Daily. KRZN = Burnett County Airport. KHYR = Hayward Municipal Airport. NTL = North Temperate Lakes Long-term Ecological Research site. UMR = Upper Mississippi River. KARV = Lakeland/Noble F. Lee Memorial Field Airport. (DOCX) [file pone.0201951.s014.docx]

| Study Area | Study Blocks | Weather Station; Type; Location | Missing Data^1^ | Questionable Data^1^ | Data substituted for Missing or Questionable Data |
| --- | --- | --- | --- | --- | --- |
| Tam | All Tam sites | NWS ID 212201; RAWS; Detroit Lakes, MN | (1) Tmin, Tmax, and P for 8–16 Apr 2009. | None | We used P, Tmin, and Tmax from Detroit Lakes, MN (station KDTL), for 8–16 Apr 2009. |
| SC | SC10DB1  SC10DD1 | NOAA ID USC00478027; GHCND; Spooner, WI | (1) Tmin for 17 Mar 2009. (2) Tmin and Tmax for all of July 2009. (3) P for 23–25 Jan 2010. | None | We used Tmin and Tmax from the nearby station in Siren, WI (station KRZN), for all of July 2009. We did not fill other, small, data gaps. |
| SC | SC12DA4  SC12DAI1 | NWS ID 470804; RAWS; Hayward, WI | (1) P for 20–21 Feb 2012. (2) Tmin and Tmax for 20 Feb–4 Mar 2012, 30 Mar–1 Apr 2012, 8–9 Apr 2012, 11 Apr 2012, and 15 Apr 2012. (3) Tmin, Tmax, and P for 1–6 Jan 2011. |  | We used P, Tmin, and Tmax from Hayward, WI (station KHYR), for all data gaps. |
| SC | SC4DA3  SC4DAI2  SC4DB9  SC4DBI2 | NWS ID 470602; RAWS; Lind, WI | (1) Tmin, and Tmax for 9 Apr–3 May 2008. | None | We did not patch in data for missing temperature records. |
| NTL | TRL1DA1  TRL1DB1  TRL2DA1  TRL2DB1  TRL3DA1  TRL3DB1  TRL3DC1 | NWS ID 471002; RAWS; Woodruff, WI | (1) P from 12–14 May 2011. (2) Tmin and Tmax from 11–15 May 2011. (3) P, Tmin, and Tmax for 28–29 Mar 2011. | None | We used data averaged from Arbor Vitae, WI (station KARV), and Minocqua, WI (GHCND:USC00475516), for all data gaps as Woodruff is midway between these two stations. |
| UMR | TrNWRDA1 | NOAA ID USC00472165; GHCND; Dodge, WI | (1) P, Tmin, and Tmax for 31 May 2011. | Tmin for 16 Jan 2009 (-37.8 C) was noticeably lower for all other records during 2008–2012. | Tmin for 16 Jan 2009 for all stations in the surrounding area was higher than the outlier recorded for Dodge. We used the Tmin (31.1 C) recorded at the two nearest stations, which also was the lowest among the surrounding stations. (1)We did not patch in data for 31 May 2011. |
| UMR | UMRP4 | NOAA ID USC00470124; GHCND; Alma Dam 4, WI | (1) Tmin for 16 Mar 2012, 31 May 2012. (2) Tmax for 16 Mar 2012, 31 May 2012, and 19 July 2012. |  | We did not patch in data for missing temperature records because they were sufficiently isolated in time to have little effect on the summary statistics we calculated. |
| UMR | UMRP7  PSP1 | NOAA ID USC00478589; GHCND; Trempealeau, WI | (1) Tmax for 12 and 28 Mar 2012, 29 Apr 2012. (2) Tmin and Tmax for 30 May 2012. | Tmin for 16 Jan 2009 (-36.7 C) noticeably lower than in other years, plausible based on data from stations from the surrounding area. | We did not patch in data for missing temperature records because they were sufficiently isolated in time to have little effect on the summary statistics we calculated. |
| UMR | UMRP10 | NOAA ID USC00476827; GHCND; Prairie du Chien, WI | (1) Tmin for 2 Jan 2010. | None | We did not patch in data for missing temperature records because they were sufficiently isolated in time to have little effect on the summary statistics we calculated. |

^1^ We did not include dates for small data gaps unless we incorporated substitute data.
