# Supplementary material for: Multi-year data from satellite- and ground-based sensors show details and scale matter in assessing climate’s effects on wetland surface water, amphibians, and landscape conditions
Source: PLoS One. 2018 Sep 7;13(9):e0201951. doi: 10.1371/journal.pone.0201951 (PMC6128473; doi:10.1371/journal.pone.0201951)
Supplement: S5 Table — Source satellite data for the normalized vegetation index product were provided as seven-day composites on a rolling schedule across years. Source satellite data for snow and evapotranspiration products were provided as eight-day products beginning on January 1 each year. Air temperature and precipitation were daily weather-station data that we summarized at eight-day intervals for field-based analyses and seven-day intervals for remote sensing-based analyses. “Start of Month” is provided for context. (DOCX) [file pone.0201951.s015.docx]

| Week Number | Days of Year | Start of Month (non-leap years^1^) | 8-Day Interval | Days of Year | Week Assigned^2^ |
| --- | --- | --- | --- | --- | --- |
| 1 | 1–7 | Day 1 = January 1 | 1 | 1–8 | 1 |
| 2 | 8–14 |  | 2 | 9–16 | 2 |
| 3 | 15–21 |  | 3 | 17–24 | 3 |
| 4 | 22–28 |  | 4 | 25–32 | 4 |
| 5 | 29–35 | Day 32 = Feb 1 | 5 | 33–40 | 6 |
| 6 | 36–42 |  | 6 | 41–48 | 7 |
| 7 | 43–49 |  | 7 | 49–56 | 8 |
| 8 | 50–56 |  | 8 | 57–64 | 9 |
| 9 | 57–63 | Day 60 = March 1 | 9 | 65–72 | 10 |
| 10 | 64–70 |  | 10 | 73–80 | 11 |
| 11 | 71–77 |  | 11 | 81–88 | 12 |
| 12 | 78–84 |  | 12 | 89–96 | 14 |
| 13 | 85–91 | Day 91 = April 1 | 13 | 97–104 | 15 |
| 14 | 92–98 |  | 14 | 105–112 | 16 |
| 15 | 99–105 |  | 15 | 113–120 | 17 |
| 16 | 106–112 |  | 16 | 121–128 | 18 |
| 17 | 113–119 |  | 17 | 129–136 | 19 |
| 18 | 120–126 | Day 121 = May 1 | 18 | 137–144 | 20 |
| 19 | 127–133 |  | 19 | 145–152 | 22 |
| 20 | 134–140 |  | 20 | 153–160 | 23 |
| 21 | 141–147 |  | 21 | 161–168 | 24 |
| 22 | 148–154 | Day 152 = June 1 | 22 | 169–176 | 25 |
| 23 | 155–161 |  | 23 | 177–184 | 26 |
| 24 | 162–168 |  | 24 | 185–192 | 27 |
| 25 | 169–175 |  | 25 | 193–200 | 28 |
| 26 | 176–182 | Day 182 = July 1 | 26 | 201–208 | 30 |
| 27 | 183–189 |  | 27 | 209–216 | 31 |
| 28 | 190–196 |  | 28 | 217–224 | 32 |
| 29 | 197–203 |  | 29 | 225–232 | 33 |
| 30 | 204–210 |  | 30 | 233–240 | 34 |
| 31 | 211–217 | Day 213 = August 1 |  |  |  |
| 32 | 218–224 |  |  |  |  |
| 33 | 225–231 |  |  |  |  |
| 34 | 232–238 |  |  |  |  |

^1^The first two columns remain the same for leap years, but day number is incremented by 1 in the “Start of Month” column for March 1 and all subsequent months.

^2^No eight-day intervals were assigned to weeks 5, 13, 21, and 29 because four fewer eight-day intervals exist than weeks from January through August.
