# Supplementary figures and images for: Multi-year data from satellite- and ground-based sensors show details and scale matter in assessing climate’s effects on wetland surface water, amphibians, and landscape conditions
Source: PLoS One. 2018 Sep 7;13(9):e0201951. doi: 10.1371/journal.pone.0201951 (PMC6128473; doi:10.1371/journal.pone.0201951)

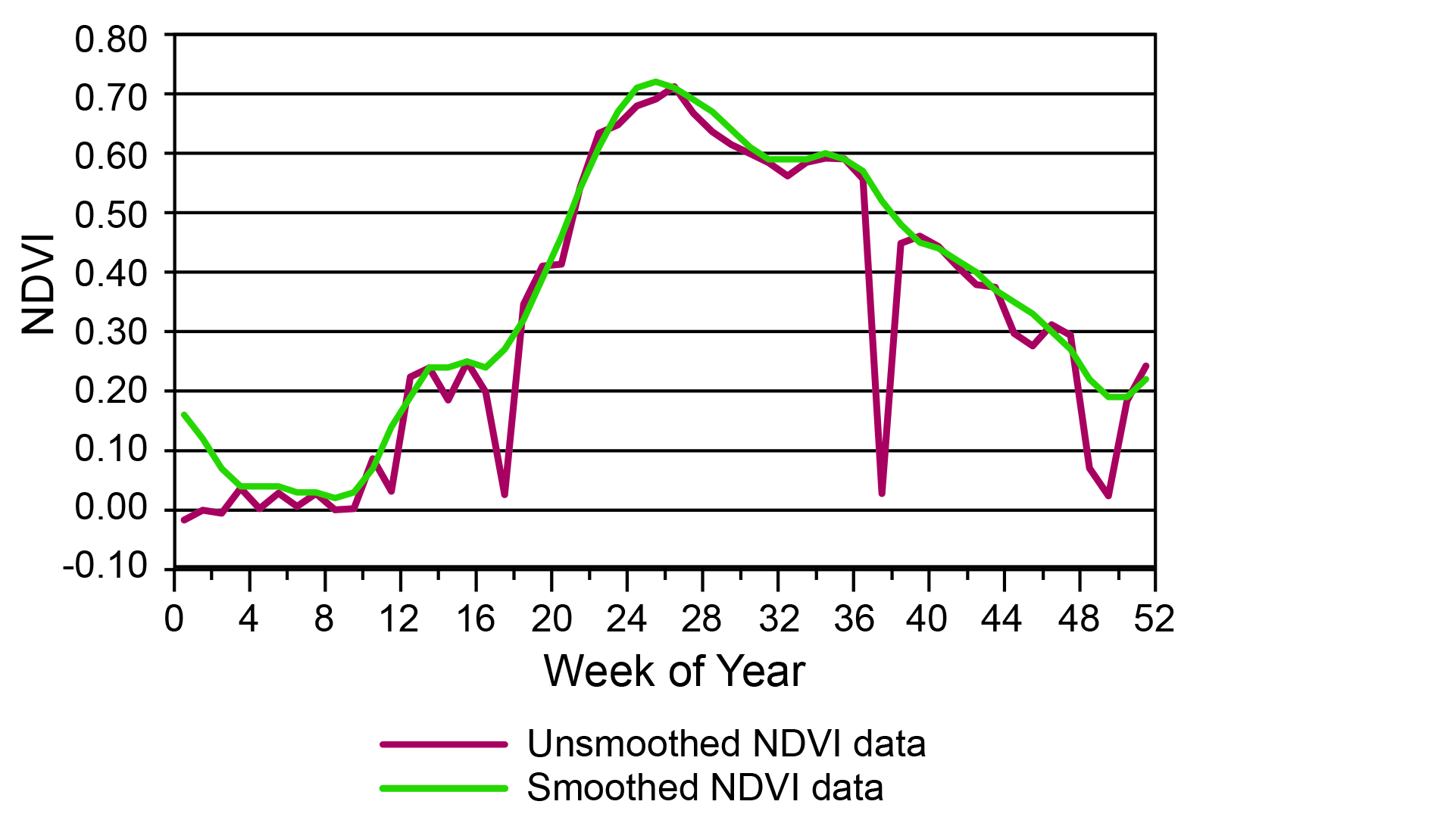

Supplement: S1 Fig — (TIF) [file pone.0201951.s016.tif]

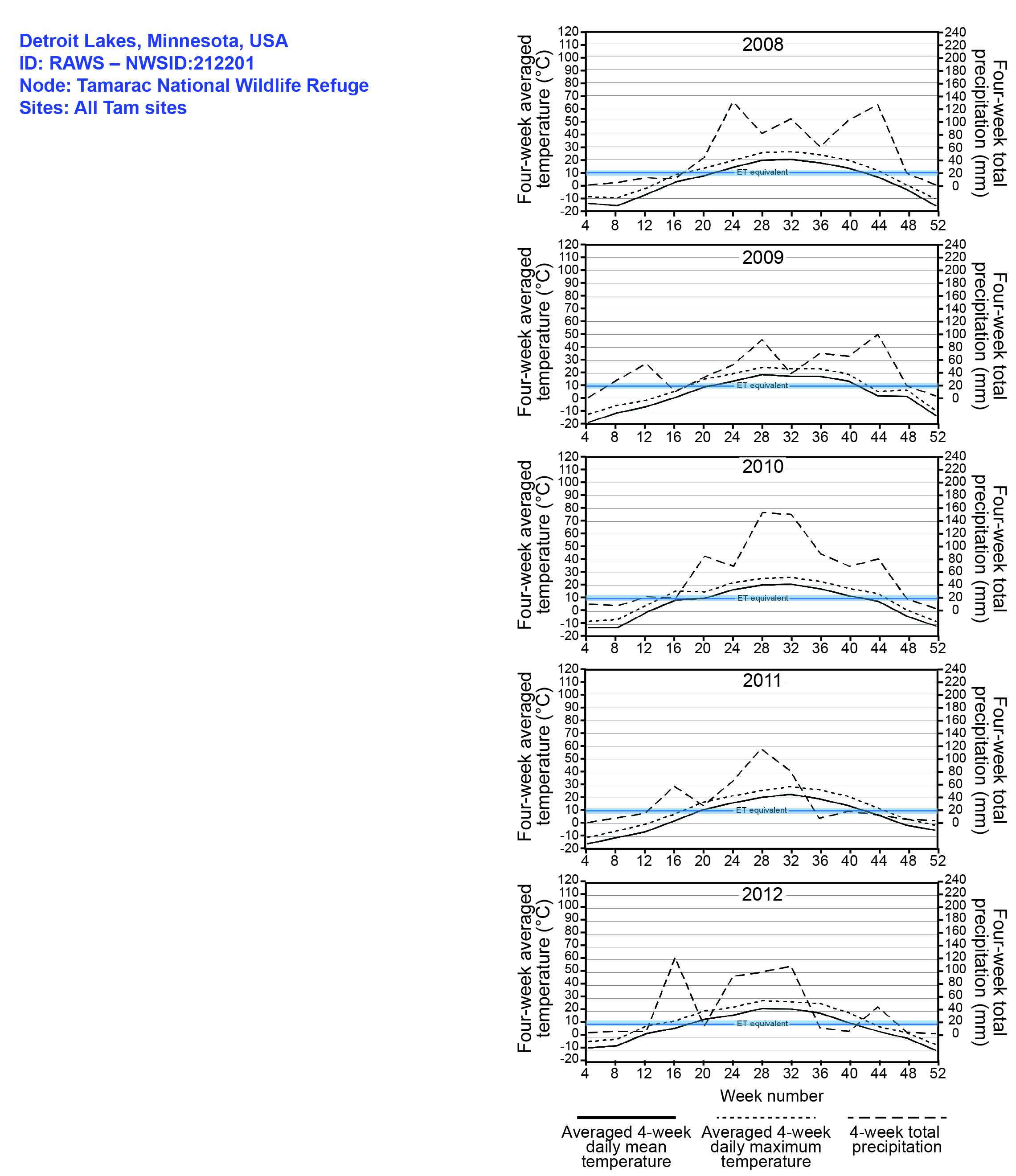

Supplement: S8 Fig — (TIF) [file pone.0201951.s023.tif]

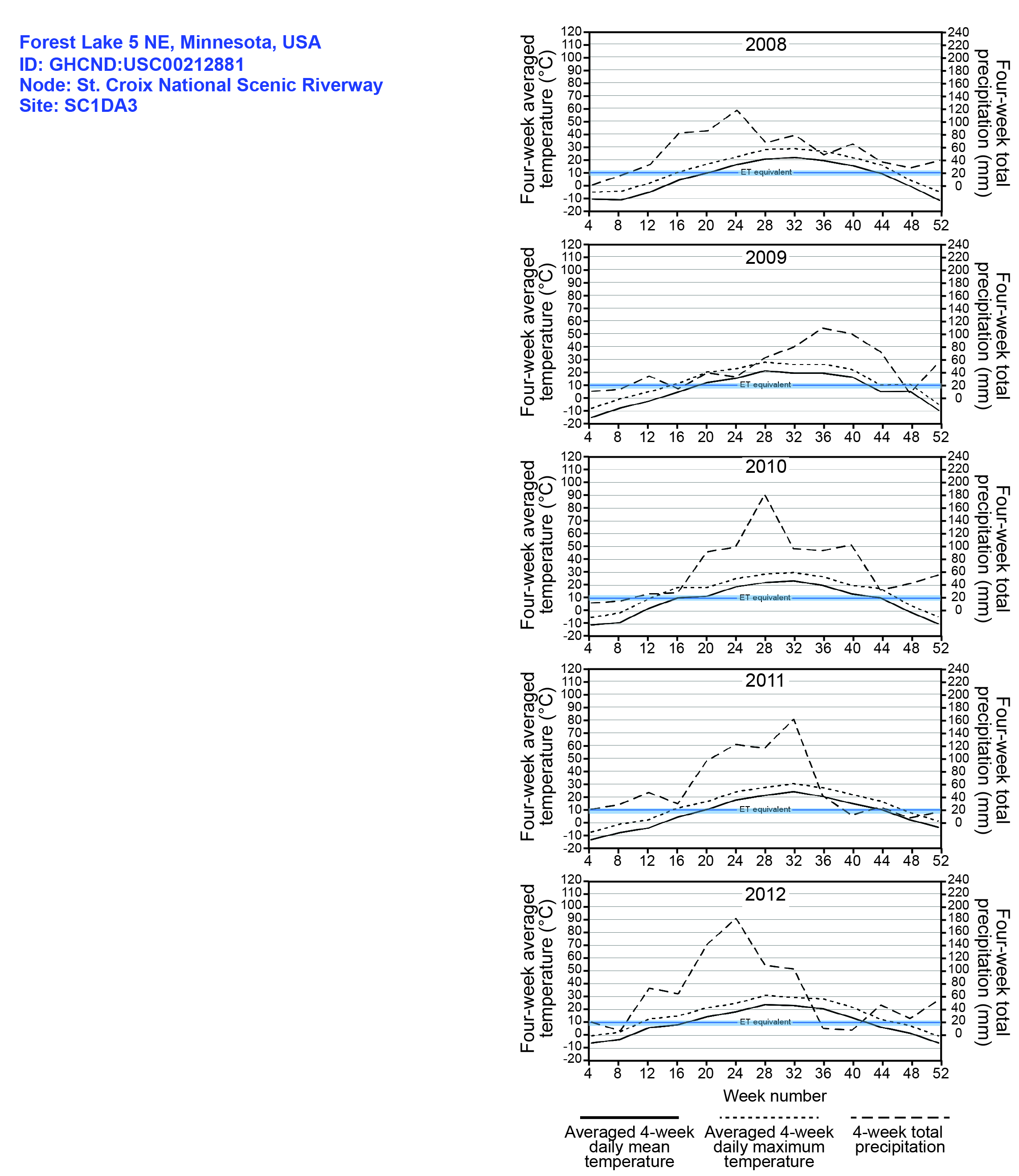

Supplement: S9 Fig — (TIF) [file pone.0201951.s024.tif]

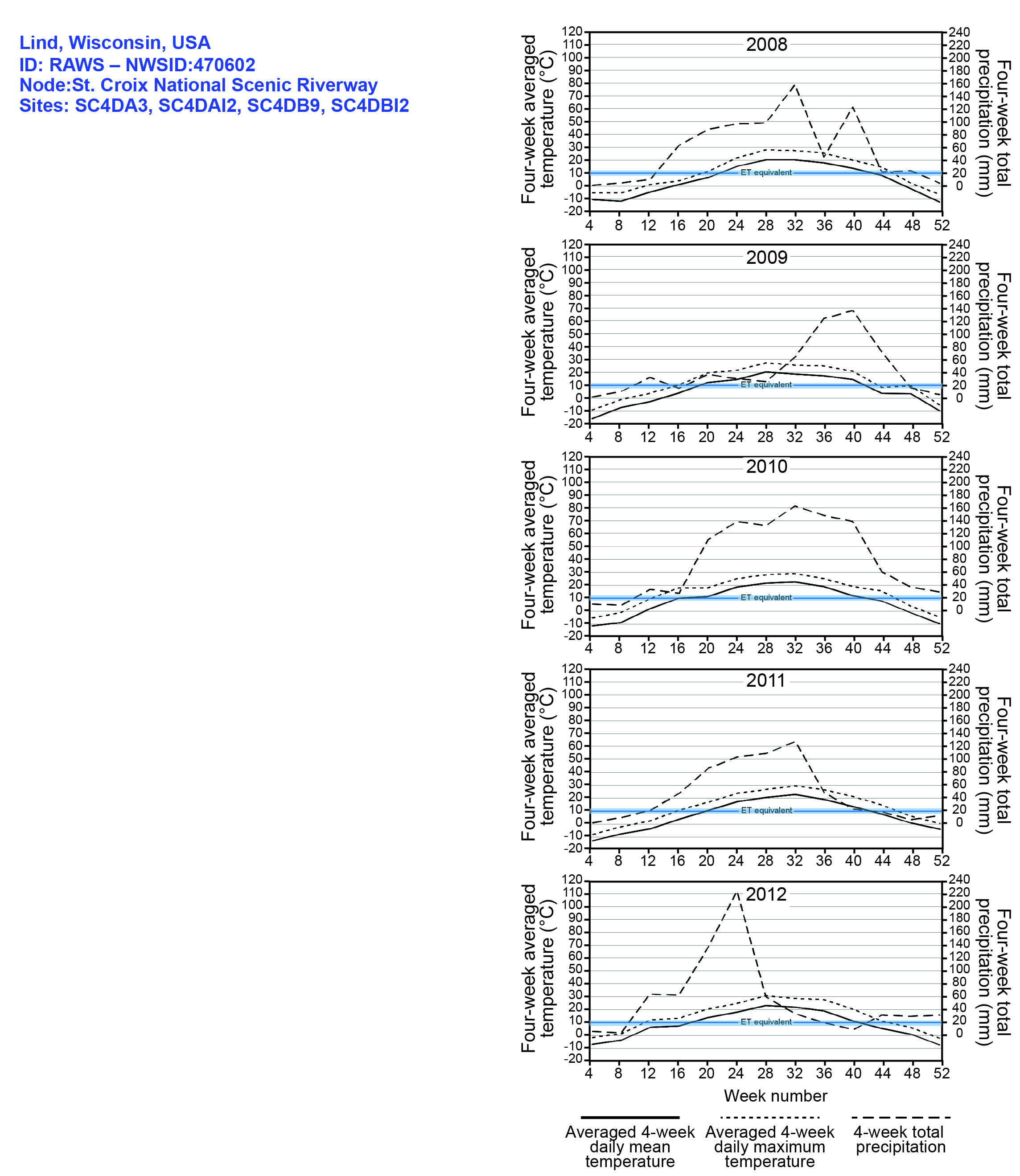

Supplement: S10 Fig — (TIF) [file pone.0201951.s025.tif]

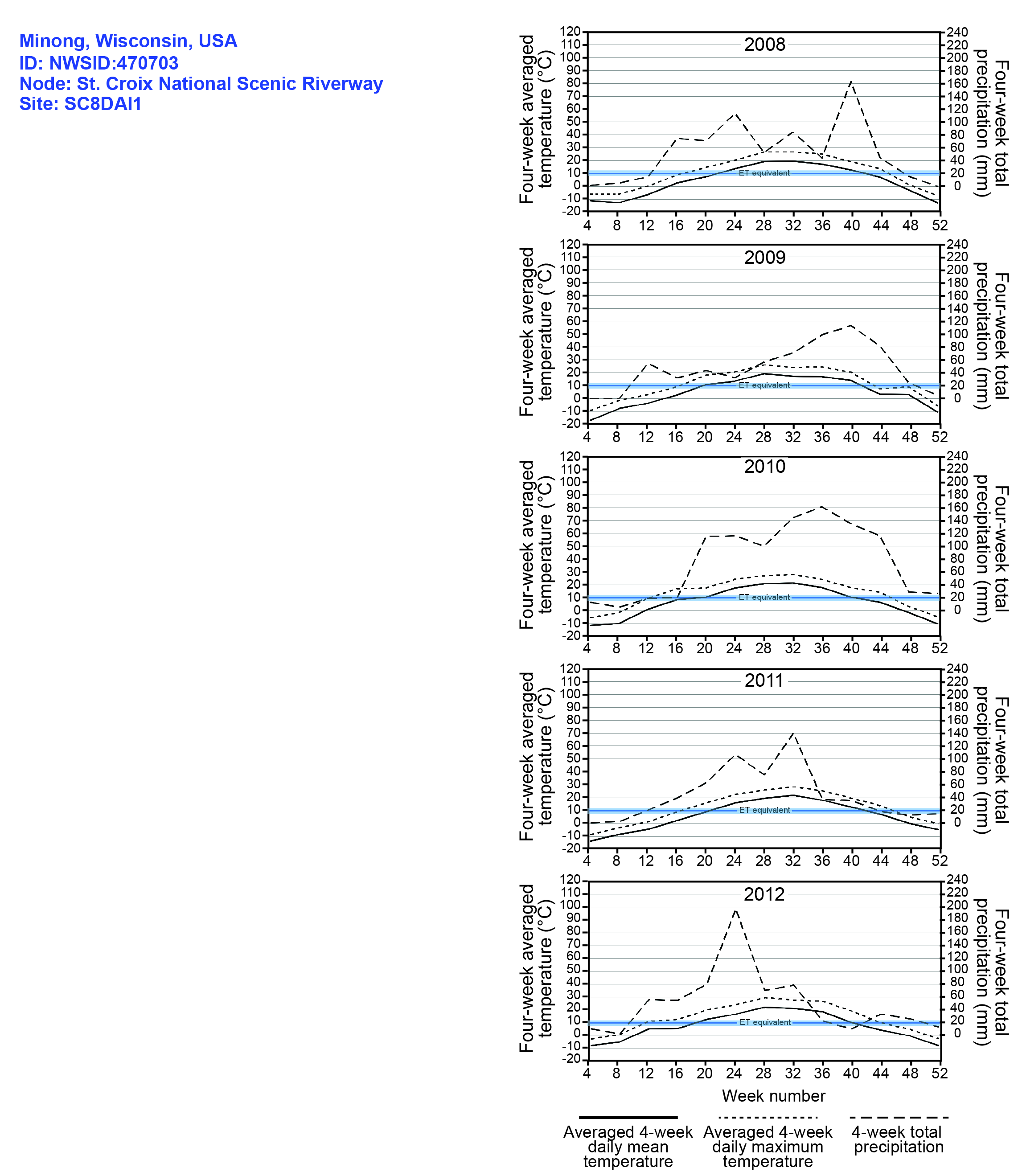

Supplement: S11 Fig — (TIF) [file pone.0201951.s026.tif]

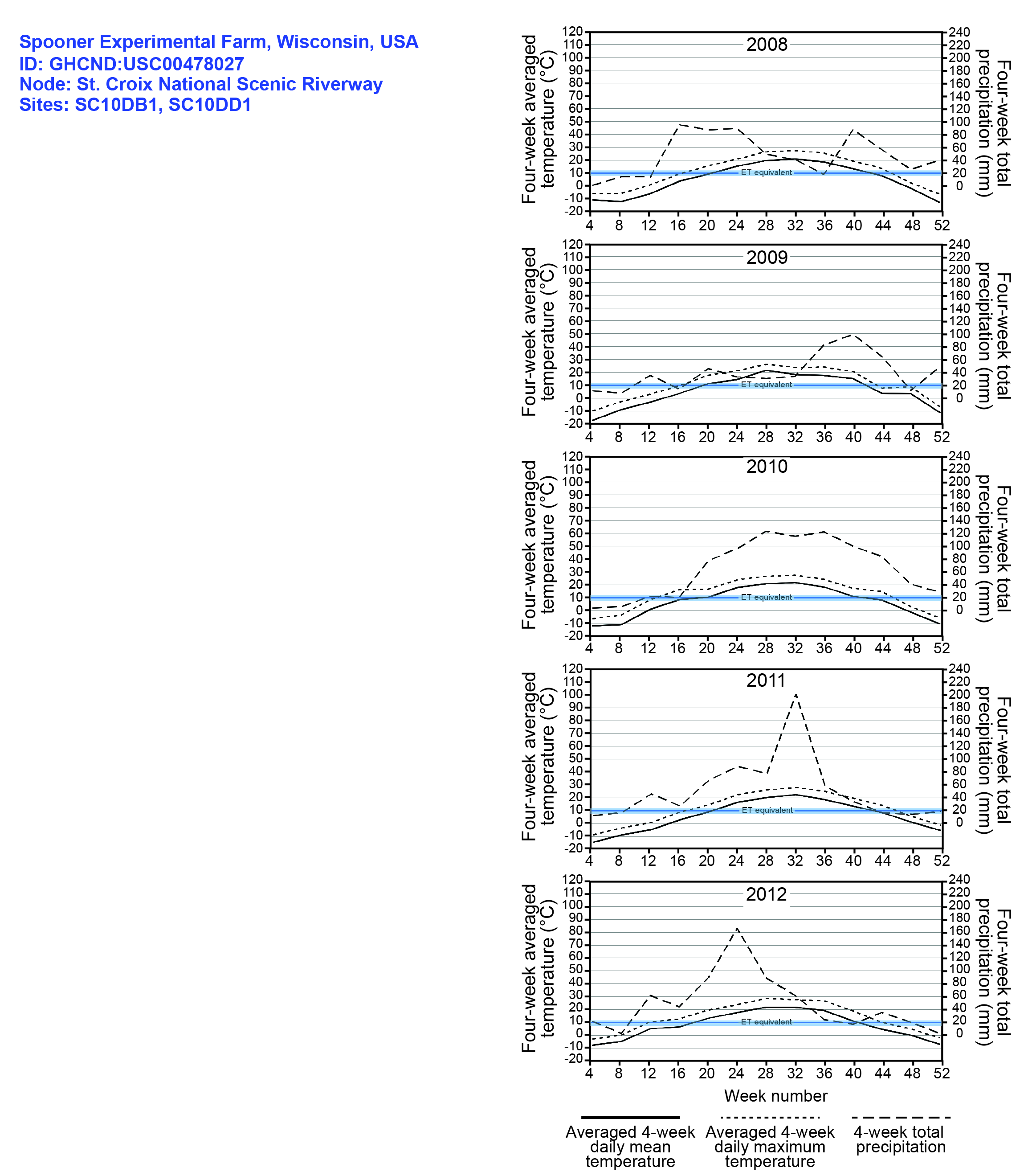

Supplement: S12 Fig — (TIF) [file pone.0201951.s027.tif]

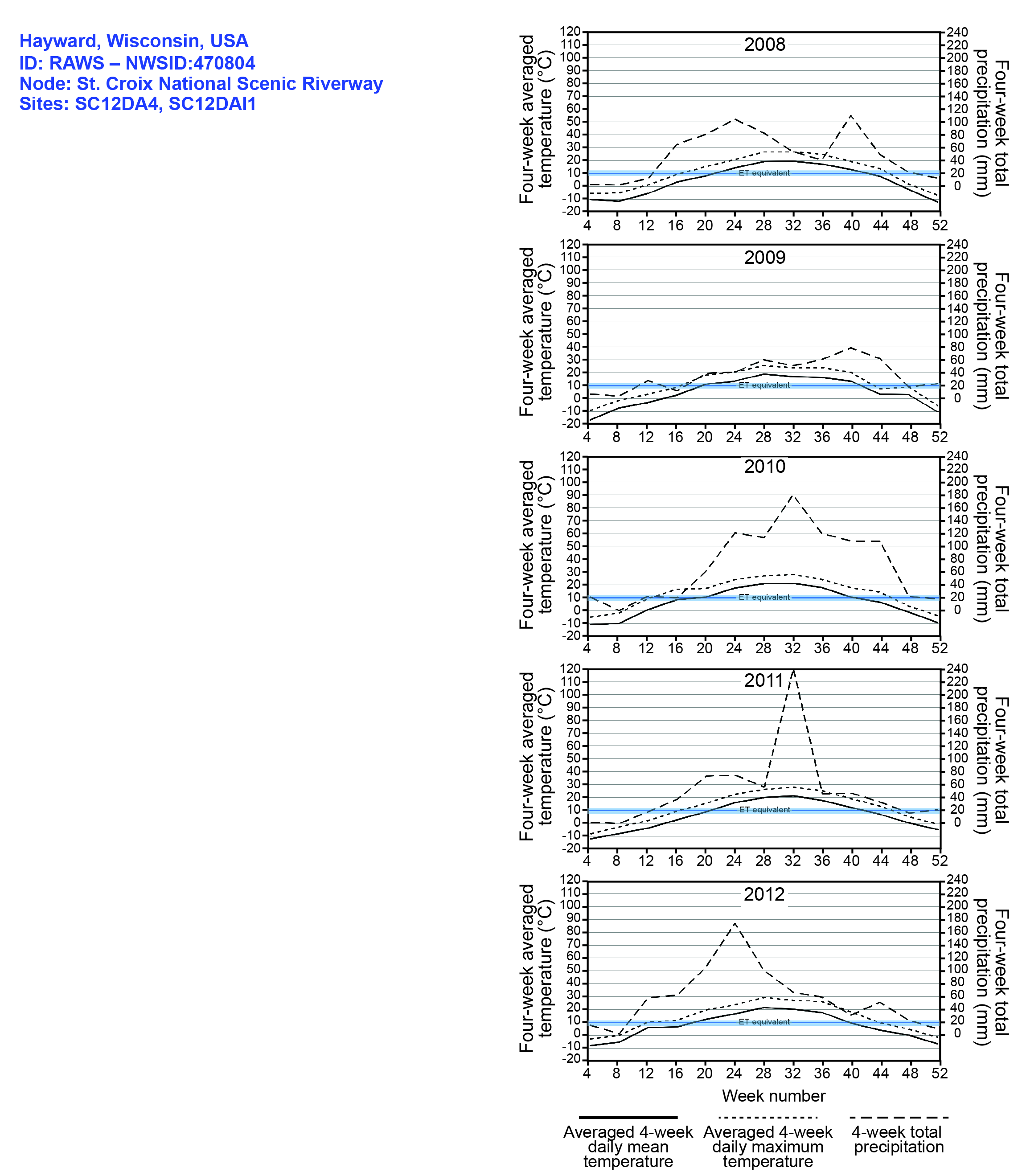

Supplement: S13 Fig — (TIF) [file pone.0201951.s028.tif]

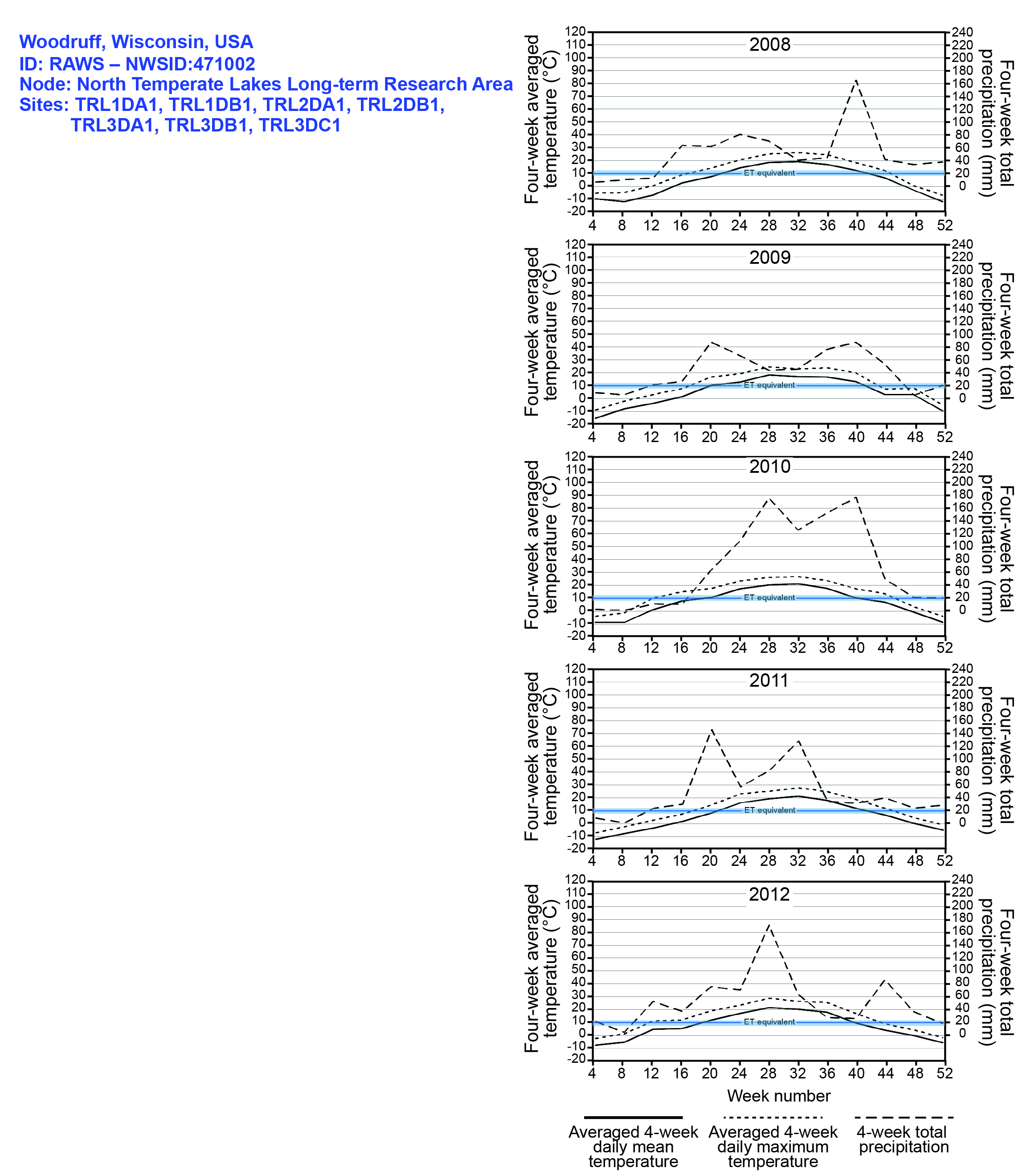

Supplement: S14 Fig — (TIF) [file pone.0201951.s029.tif]

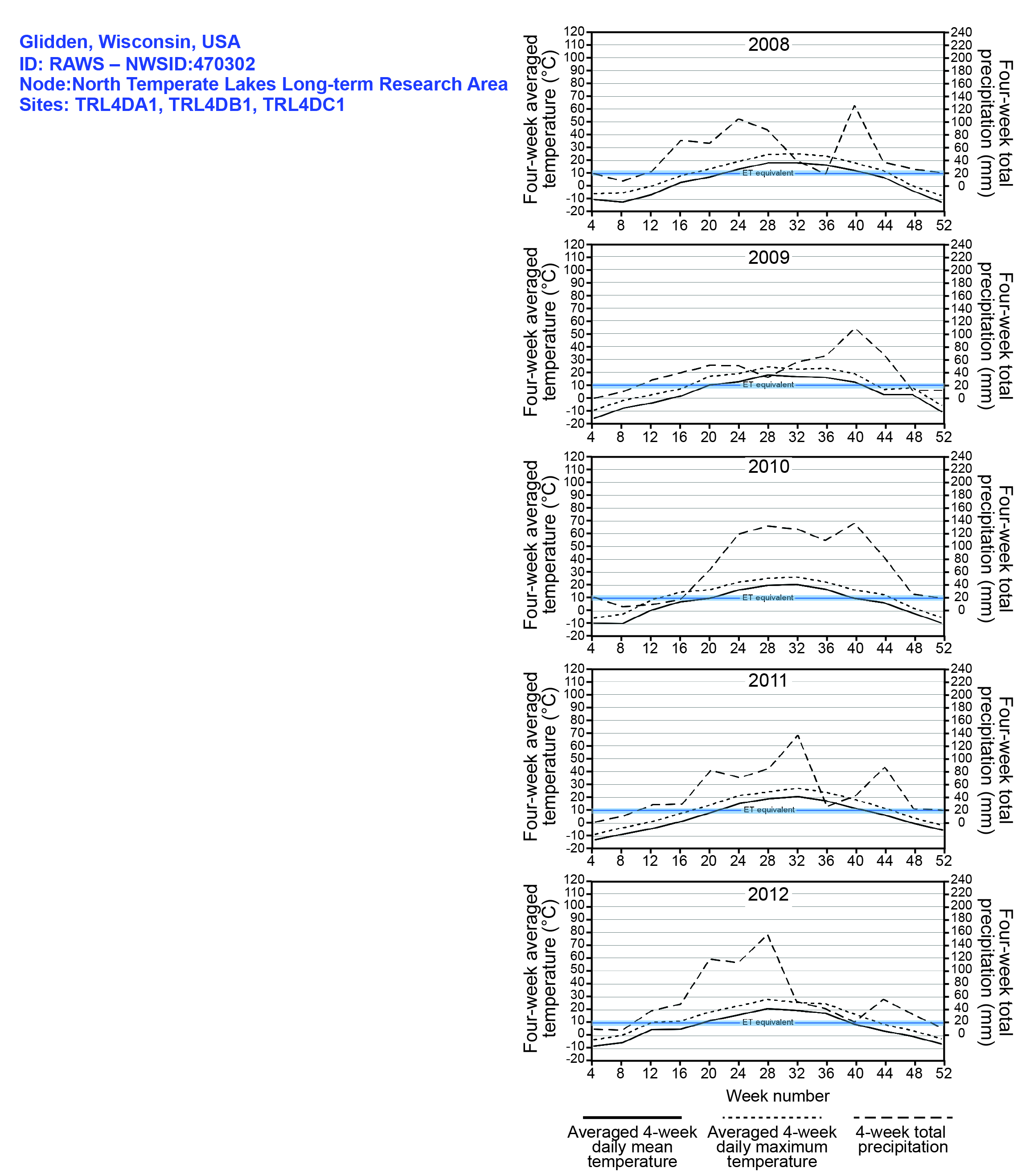

Supplement: S15 Fig — (TIF) [file pone.0201951.s030.tif]

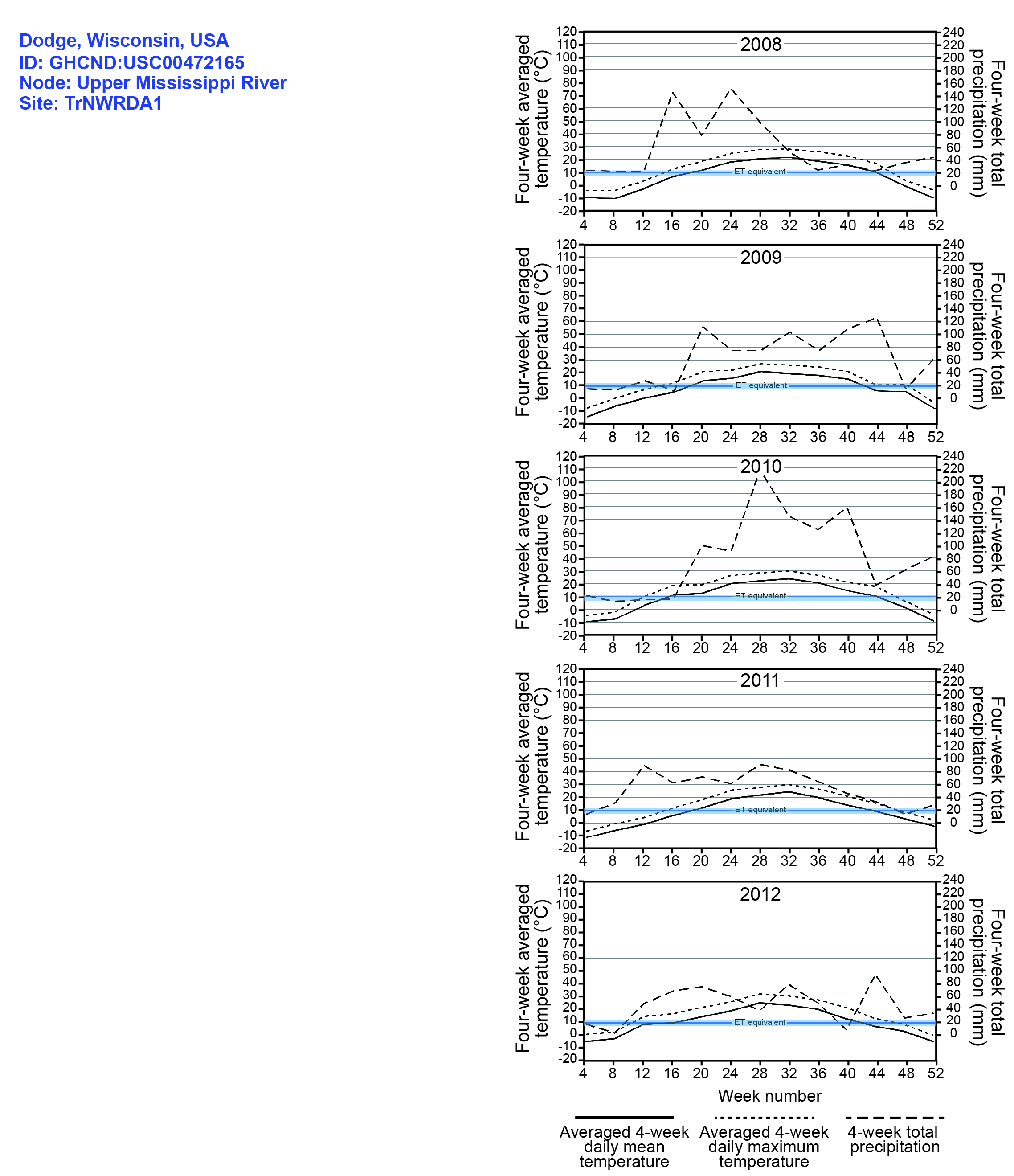

Supplement: S16 Fig — (TIF) [file pone.0201951.s031.tif]

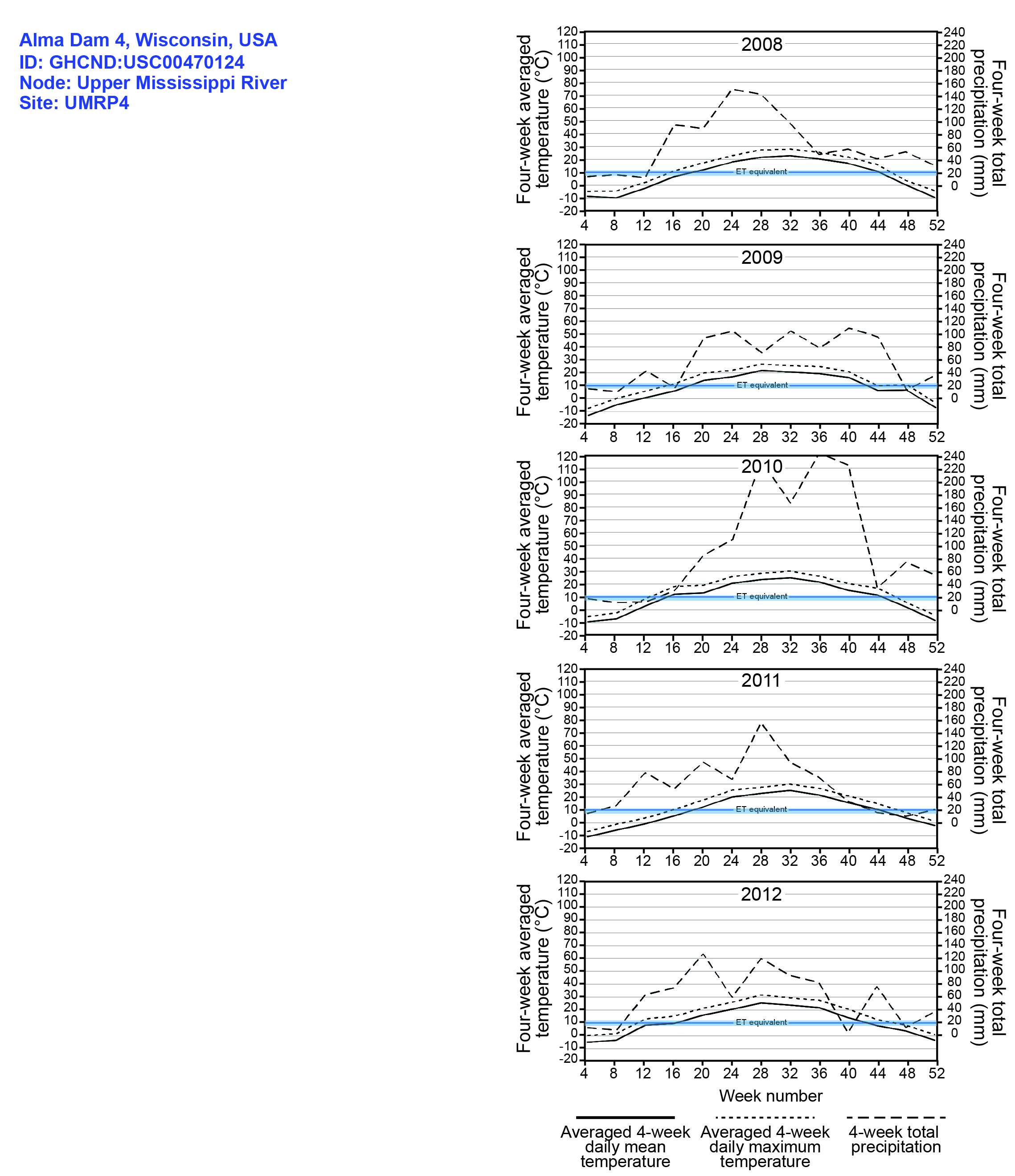

Supplement: S17 Fig — (TIF) [file pone.0201951.s032.tif]

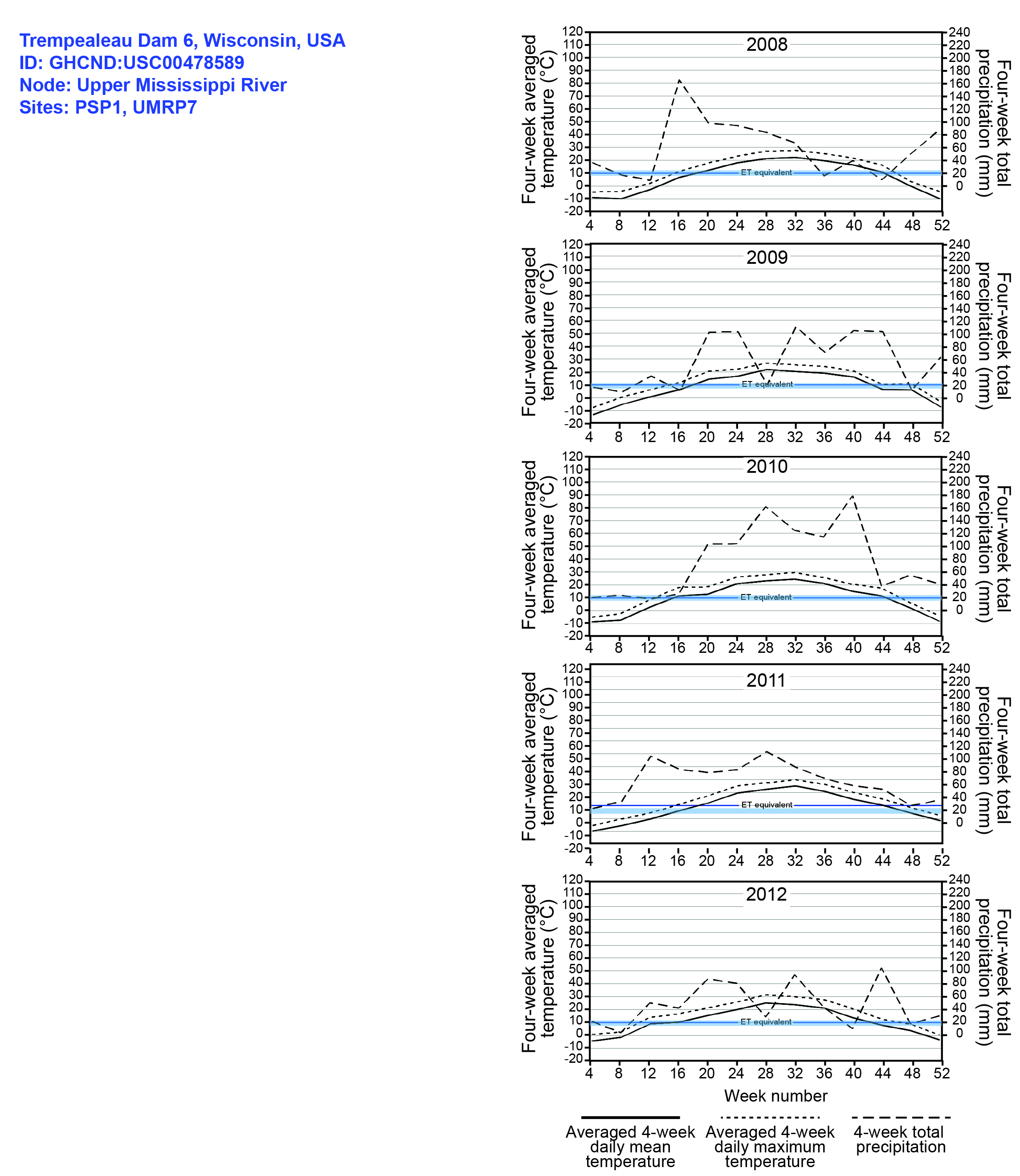

Supplement: S18 Fig — (TIF) [file pone.0201951.s033.tif]

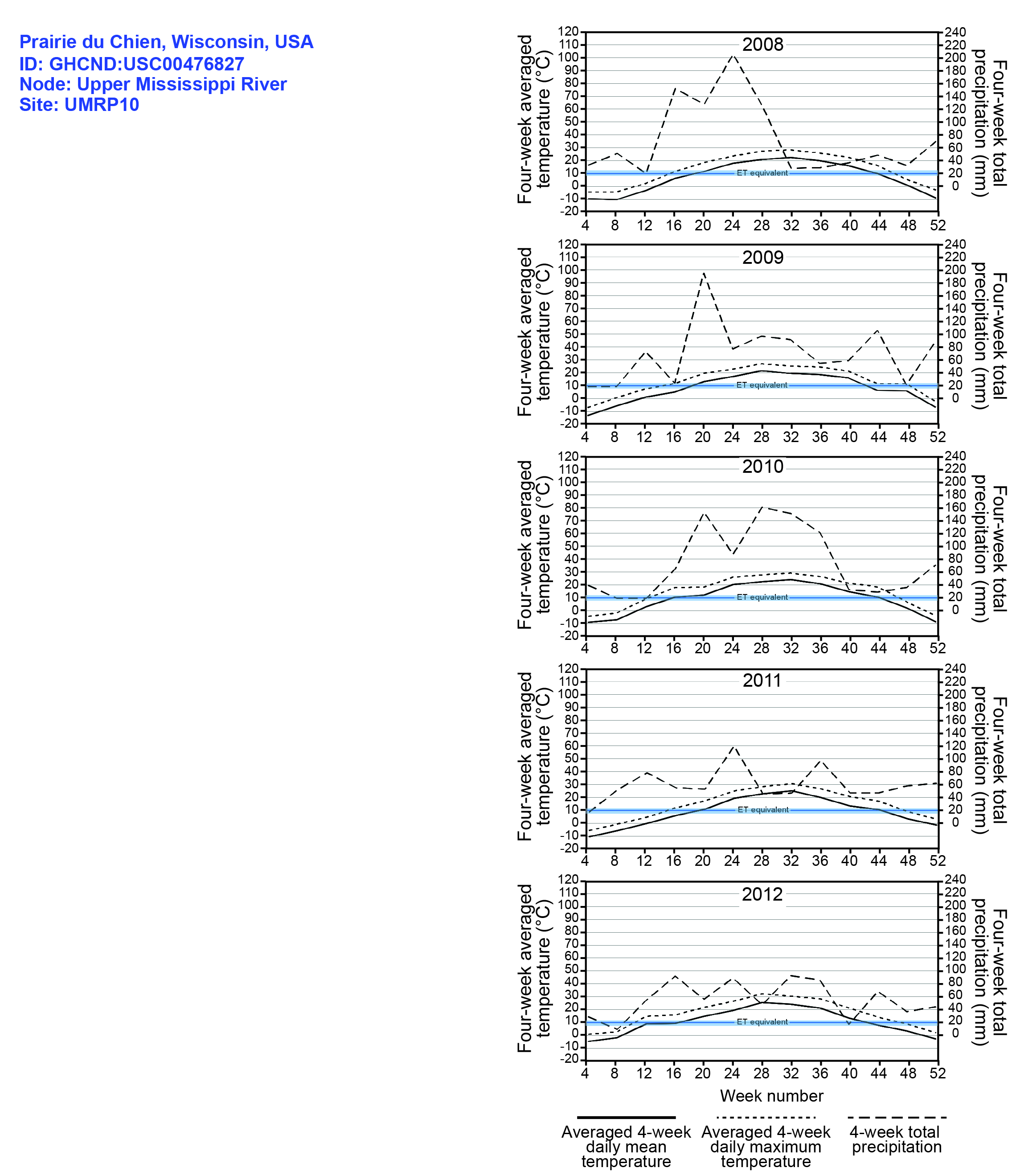

Supplement: S19 Fig — (TIF) [file pone.0201951.s034.tif]
